# Inhibition of CDK9 enhances AML cell death induced by combined venetoclax and azacitidine

**DOI:** 10.1002/1878-0261.70124

**Published:** 2025-09-16

**Authors:** Shuangshuang Wu, Jianlei Zhao, Aaban Asfar Azmi, Avanti Gupte, Jenna Thibodeau, Shuang Liu, Jinli Yang, Guan Wang, Holly Edwards, Lisa A. Polin, Juiwanna Kushner, Sijana H. Dzinic, Kathryn White, Julie Boerner, Maik Hüttemann, Jay Yang, Yue Wang, Jeffrey W. Taub, Yubin Ge

**Affiliations:** ^1^ Department of Pediatric Hematology Children's Medical Center, The First Hospital of Jilin University Changchun China; ^2^ Department of Oncology Wayne State University School of Medicine Detroit MI USA; ^3^ Molecular Therapeutics Program, Barbara Ann Karmanos Cancer Institute Wayne State University School of Medicine Detroit MI USA; ^4^ Division of Pediatric Hematology/Oncology Children's Hospital of Michigan Detroit MI USA; ^5^ Department of Pediatrics Central Michigan University College of Medicine Mt. Pleasant MI USA; ^6^ Cancer Biology Graduate Program Wayne State University School of Medicine Detroit MI USA; ^7^ National Engineering Laboratory for AIDS Vaccine, Key Laboratory for Molecular Enzymology and Engineering, the Ministry of Education, School of Life Sciences Jilin University Changchun China; ^8^ Center for Molecular Medicine and Genetics Wayne State University School of Medicine Detroit MI USA; ^9^ Department of Pediatrics Wayne State University School of Medicine Detroit MI USA

**Keywords:** acute myeloid leukemia, azacitidine, AZD4573, venetoclax

## Abstract

Relapsed/refractory (R/R) disease is a major hurdle to long‐term survival of acute myeloid leukemia (AML) patients treated with intensive cytarabine (AraC)‐based chemotherapy. R/R AML salvage treatment with venetoclax (VEN) + azacitidine (AZA) results in overall response rates between 20% and 60%, and responses are not durable, highlighting the need for new therapies. Here, we report elevated mTORC1 signaling in AraC‐resistant AML cell lines, primary AML patient samples, and patient‐derived xenograft (PDX) AML cells derived from patients at relapse postchemotherapy. The CDK9 inhibitor AZD4573 suppresses mTORC1 signaling and downregulates c‐MYC and MCL‐1, inducing AraC‐resistant AML cell death. AZD4573 in combination with VEN + AZA significantly increases AML cell death compared to any of the two‐drug combinations and suppresses AML progenitor cells but spares normal hematopoietic progenitor cells. The efficacy of this triple combination remains even with a 10‐fold reduction of VEN concentration. The roles of MCL‐1 and c‐MYC in the three‐drug combination were confirmed by knockdown. This study demonstrates that AZD4573 enhances the activity of VEN + AZA against AraC‐resistant AML by downregulating c‐MYC and MCL‐1 and to a lesser extent cellular respiration.

## Graphical abstract

The CDK9 inhibitor AZD4573 downregulates c‐MYC and MCL‐1 to induce death of cytarabine (AraC)‐resistant AML cells. This enhances VEN + AZA‐induced cell death significantly more than any combination of two of the three drugs in AraC‐resistant AML cells.

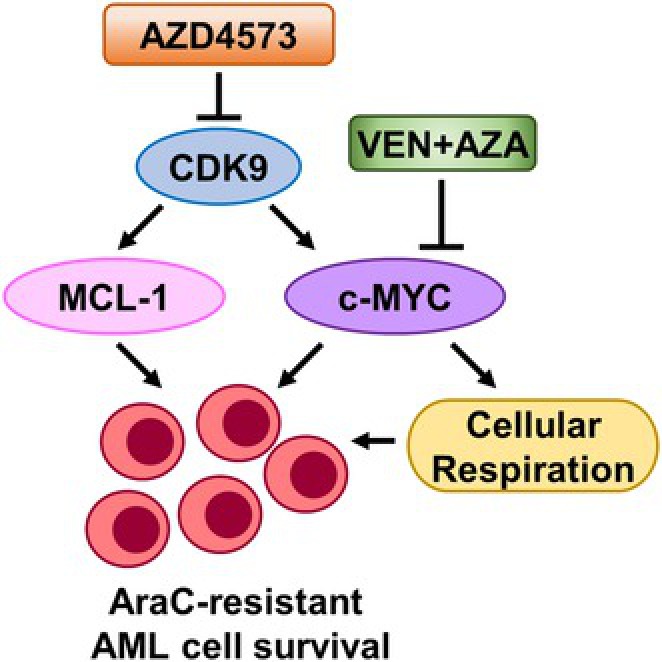

**Abbreviations**
AML: acute myeloid leukemia
AraC: cytarabine
AZA: azacitidine
CDK9: cyclin‐dependent kinase 9
CFUs: colony formation units
CMST: cellular mito stress test
FITC: fluorescein isothiocyanate
NTC: nontarget‐negative control
OCR: oxygen consumption rate
PI: propidium iodide
P‐TEFb: positive transcription elongation factor b
R/R: relapsed/refractory
RFP: red fluorescent protein
SRC: spare respiratory capacity
VEN: venetoclax

## 1 Introduction

For several decades, the standard treatment for acute myeloid leukemia (AML) patients has been standard intensive chemotherapy with cytarabine (AraC) + anthracycline. Up to 80% of patients achieve complete remission poststandard intensive chemotherapy, though most patients develop relapsed and refractory (R/R) disease which is associated with a dismal prognosis. The combination of venetoclax (VEN) + azacitidine (AZA) is now considered a standard salvage option for patients previously treated with cytarabine‐based intensive chemotherapy. However, at best, only about 60% respond and responses tend to be short‐lived [1, 2, 3, 4, 5, 6], highlighting the need of strategies to enhance the antileukemic activity of VEN + AZA against R/R AML postchemotherapy.

c‐MYC overexpression is associated with chemoresistance in AML [7, 8]. Targeting c‐MYC has been demonstrated to enhance the activity of VEN as well as VEN + AZA [9, 10, 11]. At relapse, MCL‐1 is upregulated in about half of AML patients [12] and its upregulation is a mechanism of resistance to VEN [13]. We previously demonstrated that combined inhibition of c‐MYC and MCL‐1 has antileukemic activity against AraC‐resistant (AraC‐R) AML cells [14]. mTOR plays an important role in leukemia stem cell function [15]. It has been recently reported that dual mTORC1/2 inhibition synergistically enhances AML cell death induced by VEN [16]. Furthermore, a previous clinical study demonstrates that mTOR inhibitor everolimus in combination with azacitidine is tolerable, with promising clinical activity in advanced AML [17]. Direct targeting of c‐MYC has been a challenge due to its lack of an enzymatically active site, partial redundancy of the different MYC family members, and nuclear localization, among others [18]. As such, indirect methods of targeting c‐MYC have been the most commonly used approach. Targeting of MCL‐1 has also recently taken a similar approach due to the cardiotoxicity of direct inhibition of MCL‐1. Thus, an approach to simultaneously target c‐MYC, MCL‐1, and mTOR may potently enhance the antileukemic activity of VEN + AZA against AraC‐resistant AML cells.

Cyclin‐dependent kinase 9 (CDK9) is a protein kinase and a key component of positive transcription elongation factor b (P‐TEFb). CDK9 plays a critical role in gene transcription and inhibition of CDK9 leads to temporary shutdown of gene transcription, leading to quick degradation of proteins with short half‐lives, such as MCL‐1 and c‐MYC [19]. Additionally, it was recently reported that in AML cells, CDK9 regulates mTORC1 targets [20]. These findings indicate that targeting CDK9 may represent a promising approach to simultaneously suppress c‐MYC, MCL‐1, and mTOR signaling in AML cells. AZD4573 is a selective CDK9 inhibitor that has preclinical antileukemic activity [21, 22]. In this study, we investigate the integration of AZD4573 to the combination of VEN + AZA in AraC‐resistant AML cell lines and primary patient samples.

## 2 Methods

### 2.1 Drugs

AraC and VEN were purchased from AbMole (Shanghai, China). AZD5991 and LDC000067 were purchased from MedChemExpress (Monmouth Junction, NJ, USA). AZD4573, 10058‐F4, IACS‐010759, and AZA were purchased from Selleck Chemicals (Houston, TX, USA). FCCP (Carbonyl cyanide 4‐(trifluoromethoxy) phenylhydrazone), oligomycin, rotenone, rapamycin, 2‐deoxy‐d‐glucose (2‐DG), and antimycin A were purchased from Sigma‐Aldrich (St. Louis, MO, USA).

### 2.2 Cell lines

MV4‐11 (RRID: CVCL_0064), U937 (RRID: CVCL_0007), THP‐1 (RRID: CVCL_0006), and HL‐60 (RRID: CVCL_0002) were purchased from American Type Culture Collection (Manassas, VA, USA). MOLM‐13 (RRID: CVCL_2119) cells were purchased from AddexBio (San Diego, CA, USA). Cell lines were cultured in RPMI 1640 supplemented with 10–20% fetal bovine serum (CLARK Bioscience, Claymont, DE, USA), 2 mm l‐glutamine, 100 U·mL^−1^ penicillin, and 100 μg·mL^−1^ streptomycin, in a humidified 5% CO_2_/95% environment at 37 °C. Cell lines were authenticated in the past 3 years at the Karmanos Cancer Institute's Genomics Core via the PowerPlex® 16 System (Promega, Madison, WI, USA). Cell lines were tested for the presence of mycoplasma, using the PCR method described by Uphoff and Drexler [23], every 2 weeks. All experiments were performed with mycoplasma‐free cells.

AML cell lines with acquired AraC resistance, designated MV4‐11/AraC‐R, MOLM‐13/AraC‐R, and U937/AraC‐R, were generated by exposing MV4‐11, MOLM‐13, and U937 cells to stepwise increasing concentrations of AraC, as previously described [10, 14]. MV4‐11/AraC‐R, MOLM‐13/AraC‐R, and U937/AraC‐R were cultured in the presence of 1100, 1000, and 350 nm AraC, respectively.

### 2.3 Patient samples

Diagnostic blast samples, labeled ‘AML#’, and human umbilical cord blood cells were obtained from the First Hospital of Jilin University, Changchun, China. Samples were collected from January 2023 to March 2024. Written informed consent was provided according to the Declaration of Helsinki. The study was approved by the Human Ethics Committee of the First Hospital of Jilin University (Ethical Code #2019‐128). AML patient samples were screened for gene mutations via PCR amplification and automated DNA sequencing, and cytogenetics and detection of fusion genes via real‐time PCR were performed as previously described [10, 24]. Primary patient samples were purified with Ficoll–Hypaque density centrifugation and then cultured in RPMI 1640 media plus 20% fetal bovine serum, ITS Solution (Sigma‐Aldrich), and 20% supernatant of the 5637 bladder cancer cell line (source of granulocyte‐macrophage colony‐stimulating factor, granulocyte colony‐stimulating factor, interleukin‐1 beta, macrophage colony‐stimulating factor, and stem cell factor) [24, 25, 26]. Characteristics of the individual AML patients are listed in Table S1. *In vitro* sensitivities to AraC were determined using MTT assays and the data are shown in Fig. S1.

Primary AML patient sample KCI48595 (derived from an AML patient at relapse postinduction chemotherapy and allogenic hematopoietic stem cell transplant) was retrieved from the Karmanos Cancer Institute Biobanking and Correlative Sciences Core. Written informed consent was provided according to the Declaration of Helsinki. KCI48595 cells were purified by Ficoll–Hypaque density centrifugation and then used for western blotting without *in vitro* culture.

PDX model J000106565 was purchased from Jackson Laboratory (Bar Harbor, ME, USA). This model was derived from an M4/M5 AML patient (*FLT3*‐ITD, *FLT3*‐TKD, and *NPM1* mutation) that had undergone induction chemotherapy with consolidation high‐dose cytarabine and allogenic hematopoietic stem cell transplant. J000106565 PDX cells were passaged in 8‐week‐old NSG‐SGM3 (JAX#013062, nonobese diabetic SCID gamma (NOD.Cg‐*Prkdc* ^*scid*^ *Il2rg* ^*tm1Wjl*^ Tg (CMV‐IL3, CSF2, KITLG) 1Eav/MloySzJ, Jackson Laboratory)) female mice and isolated from splenic tissue at the time of euthanasia. All mice were provided food and water *ad libitum*, given supportive fluids and supplements as needed, and housed within an AAALAC‐accredited animal facility with 24/7 veterinary care. *In vivo* cell passage was approved by the Institutional Animal Care and Use Committee at Wayne State University.

### 2.4 GSEA

Gene set enrichment analysis (GSEA) was performed using gsea software (https://www.gsea‐misgdb.org/gsea/index.jsp) [27, 28], comparing previously published proteomics data (MV4‐11/AraC‐R cells compared to parental MV4‐11 [11]) to the Hallmark gene set. The Hallmark gene set was downloaded from Molecular Signatures Database (MSigDB, https://www.gsea‐msigdb.org/gsea/msigdb/collections.jsp#H) [29, 30]. The number of permutations was set to be 1000 times. *P* < 0.05 was considered as significantly enriched.

### 2.5 Western blot

Cells were sonicated in the presence of protease and phosphatase inhibitors (Roche Diagnostics, Indianapolis, IN, USA), as previously described [31, 32]. Whole cell lysates were subjected to SDS polyacrylamide gel electrophoresis, electrophoretically transferred onto polyvinylidene difluoride membranes (Thermo Fisher Scientific, Rockford, IL, USA), and immunoblotted using anti‐c‐MYC (A5011) (Bimake.cn, Shanghai, China), ‐β‐actin (66009‐1‐Ig), ‐MCL‐1 (16225‐1‐AP) (Proteintech Group, Chicago, IL, USA), ‐cf‐Caspase 3 (9661S), ‐p‐S6 (5364S), and ‐mLST8 (3274 T) (Cell Signaling Technologies, Danvers, MA, USA). Full‐length uncropped blots are shown in Data S2 and are labeled with the corresponding figure and panel number as shown in the main manuscript.

### 2.6 Co‐immunoprecipitation

AML cells were treated with or without 40 nm AZD4573 for 12 h and then lysed using lysis buffer (1% CHAPS, 5 mm MgCl_2_, 150 mm NaCl, 1 mm EDTA, 20 mm Tris, and 0.05% Tween‐20) in the presence of protease inhibitors. Co‐immunoprecipitation of CDK9 and mLST8 was performed as previously described [33] using 2 μg of anti‐CDK9 antibody (2316S; Cell Signaling Technologies), 1 mg protein lysate, and protein A agarose beads (Roche Diagnostics). Proteins were eluted using 50 mm glycine (pH 2.0) and then analyzed by western blotting.

### 2.7 Annexin V/PI staining and flow cytometry analysis

AML cell lines and primary patient samples were treated as indicated for up to 24 h. Apoptosis was performed utilizing the annexin V‐fluorescein isothiocyanate (FITC)/propidium iodide (PI) apoptosis kit (Beckman Coulter, Brea, CA, USA), as previously described [34]. Apoptosis Positive Control Solution was purchased from Sungene Biotech Ltd. (Tianjin, China). Results are displayed as the percentage of annexin V positive cells (annexin V+/PI− cells are defined as early apoptotic cells, while annexin V+/PI+ are defined as late apoptotic or dead cells) from one experiment in triplicate. The extent and direction of the antileukemic interaction between two agents were determined by calculating the combination index (CI) using compusyn software (Combosyn Inc., Paramus, NJ, USA), in which CI < 1, CI = 1, and CI > 1 are, respectively, indicative of synergistic, additive, and antagonistic effects [34, 35].

### 2.8 Colony formation assay

Colony formation assays were carried out using methocult (catalog number 04434; STEMCELL Technologies, Vancouver, BC, Canada), as previously described [36, 37, 38]. Colony‐forming units (CFUs) were visualized utilizing an inverted microscope. Colonies containing over 50 cells were counted.

### 2.9 Lentiviral shRNA knockdown

The pMD‐VSV‐G and delta 8.2 plasmids were gifts from Dr. Dong at Tulane University. *Bak*, *Bax*, and nontarget‐negative control (NTC) shRNA lentiviral constructs were purchased from Sigma‐Aldrich. *Red fluorescent protein* (*RFP*) and *MCL‐1* cDNA lentiviral constructs were purchased from Horizon Discovery (Cambridge, UK). Lentivirus production and transduction were carried out as previously described [32]. The *Bak*/*Bax* dual knockdown (*Bak*/*Bax* KD) and NTC MV4‐11 cells were generated as previously reported [9, 10, 39].

### 2.10 CRISPR knockdown

The lentiCRISPRv2 plasmid was a gift from Feng Zhang at the Broad Institute of MIT and Harvard (Addgene plasmid 52961). CRISPR knockdown of c‐MYC and MCL‐1 was carried out as previously described [14]. Briefly, guide RNAs were designed using the CRISPR design tool (http://crispr.mit.edu). The nontarget control (NTC; 5′‐GCACTACCAGAGCTAACTCA‐3′), c‐MYC (5′‐GTATTTCTACTGCGACGAGG‐3′), and MCL‐1 (5′‐GCTTCCGCCAATCACCGCGC‐3′) vectors were generated using Feng Zhang's protocol, which is available on Addgene's website (www.addgene.org). Lentivirus production and transduction were carried out as described above, except psPAX2 (a gift from Didier Trono at the Swiss Institute of Technology, Addgene plasmid #12260) was used instead of delta 8.2.

### 2.11 Oxygen consumption rate

Cellular Mito Stress Test (CMST) was conducted using a Seahorse XFe24 flux analyzer (Agilent Technologies, Santa Clara, CA, USA) following the manufacturer's instructions and as previously described [40]. Briefly, 50 μL of 22.4 μg·mL^−1^ Cell‐Tak solution (pH 8.0; Corning Inc., Corning, NY, USA) was added to XF24 cell culture plates and incubated for 30 min at room temperature. Following incubation, Cell‐Tak was aspirated, and the wells were washed with 200 μL of sterile water. To remove water from the wells, the plates were air‐dried under the circulating sterile hood. XFe24 extracellular flux assay kit cartridge was hydrated with 1 mL of XF calibrant per well of the utility plate for at least 4 h. Cells were treated with vehicle control, AZD4573, VEN + AZA, or AZD4573 + VEN + AZA for 1 or 2 h, as indicated. Cells were seeded into the prepared XF24 plate (80 000 cells/well) with XF RPMI assay medium containing 11 mm glucose, 2 mm glutamine, and 1 mm sodium pyruvate. The plate was placed in a CO_2_‐free incubator at 37 °C for 60 min. Oxygen consumption rate (OCR) was measured in real time with the Cell Mito Stress Test Kit (Oligomycin 1.0 μm; FCCP 1.5 μm; Rotenone + Antimycin A 0.5 μm). Basal respiration, maximal respiration, spare respiratory capacity (SRC), and OCR associated with ATP production were calculated per the manufacturer's instructions.

### 2.12 Statistical analysis

The unpaired *t*‐test was used to compare differences between two groups, while one‐way ANOVA followed by Bonferroni's *post hoc* test was used when comparing more than two groups. Statistical analyses were performed utilizing graphpad prism 9.0 (GraphPad Software, Boston, MA, USA). Error bars are representative of standard error of the mean (SEM); significance was set at *P* < 0.05.

## 3 Results

### 3.1 mTOR, c‐MYC, and MCL‐1 contribute to AraC‐resistant AML cell survival

**Fig. 1:**
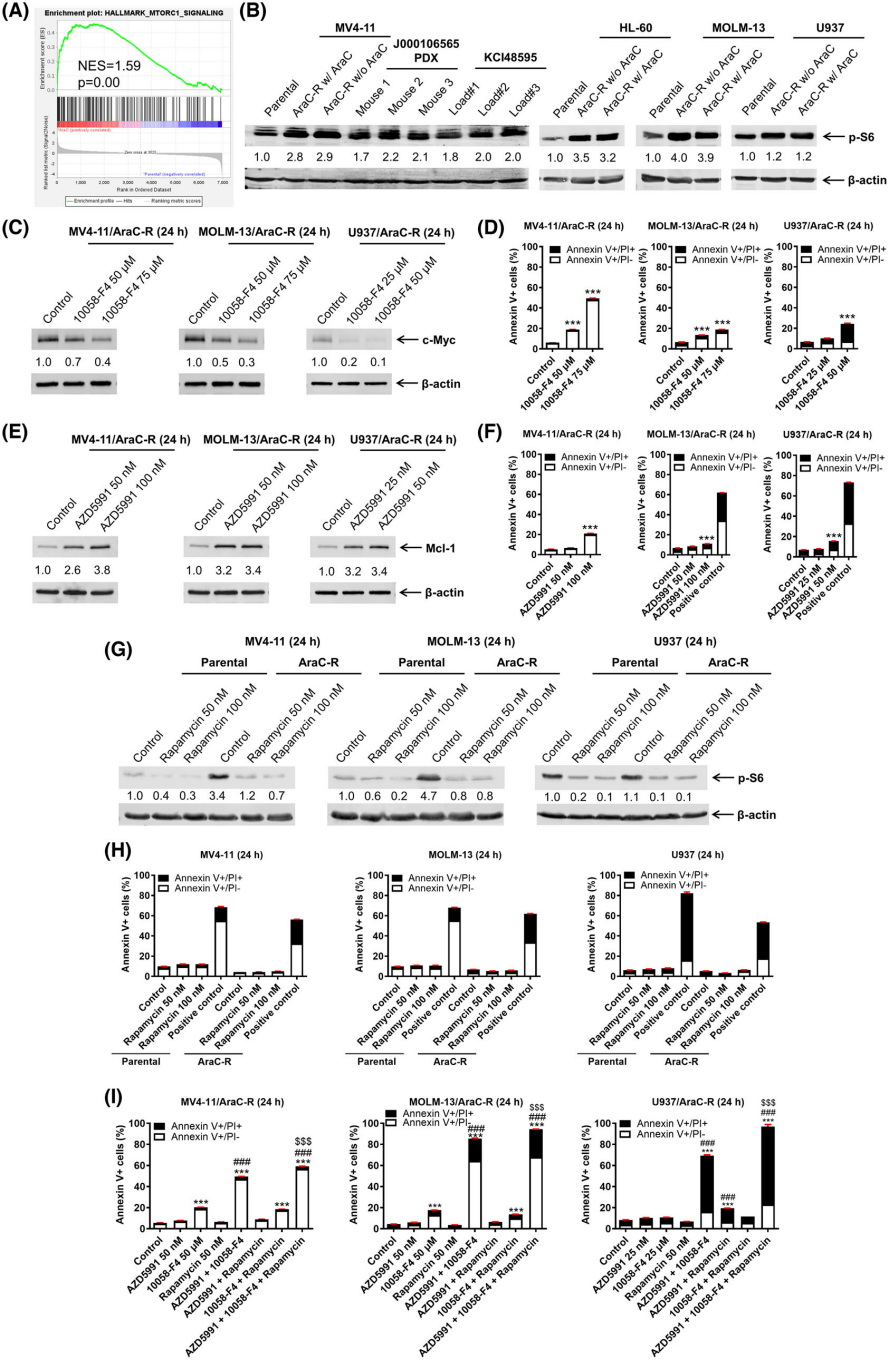
mTOR, c‐MYC, and MCL‐1 contribute to AraC‐resistant AML cell survival. (A) gsea comparing proteomics results of MV4‐11 and MV4‐11/AraC‐R cells (published in reference [11]) to the Hallmark gene set. (B) Whole cell lysates from AML cell lines, J000106565 PDX cells, and KCI48595 primary blasts were subjected to western blot and probed with anti‐p‐S6 and anti‐β‐actin antibodies. Densitometry measurements normalized to β‐actin and then compared to parental cell lines are shown below the corresponding blot. (C–H) AraC‐resistant AML cell lines were treated with 10058‐F4 or AZD5991 for 24 h; parental and AraC‐resistant AML cell lines were treated with rapamycin for 24 h. c‐MYC, MCL‐1, or p‐S6 protein levels, from whole cell lysates, were analyzed by western blot (panels C, E, and G). Densitometry measurements normalized to β‐actin and then compared to the control are shown below the corresponding blot. Annexin V‐fluorescein isothiocyanate (FITC)/propidium iodide (PI)‐stained cells were analyzed by flow cytometry (panels D, F, and H). Results are graphed as mean ± SEM from one experiment in triplicate. ***P < 0.001 compared to the control. The unpaired *t*‐test was used to determine statistical significance. (I) AraC‐resistant AML cell lines were treated with AZD5991, 10058‐F4, or rapamycin, alone or in combination, for 24 h, then stained with annexin V‐FITC/PI and analyzed by flow cytometry. Results are graphed as mean ± SEM. ***P < 0.001 compared to the control. ^###^ *P* < 0.001 compared to all corresponding single‐drug treatments. ^$$$^ *P* < 0.001 compared to all corresponding 2‐drug combinations. One‐way ANOVA followed by Bonferroni's *post hoc* test was used to determine statistical significance. Western blotting and annexin V‐FITC/PI staining and flow cytometry analysis experiments were repeated in biological replicates.

We previously published proteomics data comparing AraC‐resistant MV4‐11/AraC‐R cells to parental MV4‐11 cells [11]. We reported that proteins upregulated in the MV4‐11/AraC‐R cells showed significant enrichment among the Hallmark MYC target gene set [11]. In addition to MYC target genes, proteins upregulated in the MV4‐11/AraC‐R cells showed a significant enrichment score of 1.59 (*P* = 0.00) among the Hallmark MTORC1 target gene set (Fig. 1A). Accordingly, we assessed p‐S6 protein levels in the AraC‐resistant AML cell lines and found that compared to parental lines, p‐S6 is notably higher (Fig. 1B). Additionally, PDX cells show p‐S6 levels similar to the AraC‐resistant cell lines. These results indicate relatively increased mTOR activity in the AraC‐resistant cells.

To determine if c‐MYC plays a role in the survival of AraC‐resistant cells, AraC‐resistant cells were treated with the c‐MYC inhibitor 10058‐F4. 10058‐F4 treatment resulted in decreased c‐MYC protein levels and induction of AML cell death, though the magnitude varies from < 20% to ~ 50% when treated with 75 μm 10058‐F4 for 24 h (Fig. 1C,D). AML cell survival has been shown to be dependent on MCL‐1 and elevated levels have been associated with AML relapse [41, 42]. Thus, we treated AraC‐resistant cells with the MCL‐1 inhibitor, AZD5991. Treatment with AZD5991 increased MCL‐1 levels and resulted in significant, though minimal, induction of cell death (Fig. 1E,F). To confirm the role of mTORC1, parental and AraC‐resistant cells were treated with rapamycin, which resulted in downregulation of p‐S6, though no significant induction of AML cell death was detected (Fig. 1G,H). While targeting of c‐MYC, MCL‐1, or mTORC1 alone resulted in minimal induction of cell death, combined treatment with AZD5991 and 10058‐F4 or 10085‐F4 and rapamycin significantly induced AML cell death (Fig. 1I). Furthermore, simultaneous treatment with all three inhibitors resulted in significantly more AML cell death than either two‐drug combination or single‐drug treatment, demonstrating that simultaneous targeting of c‐MYC, MCL‐1, and mTOR may be a promising strategy to target AraC‐resistant AML cells.

### 3.2 AZD4573 downregulates c‐MYC, MCL‐1, and p‐S6 in AraC‐resistant AML cells and induces cell death

**Fig. 2:**
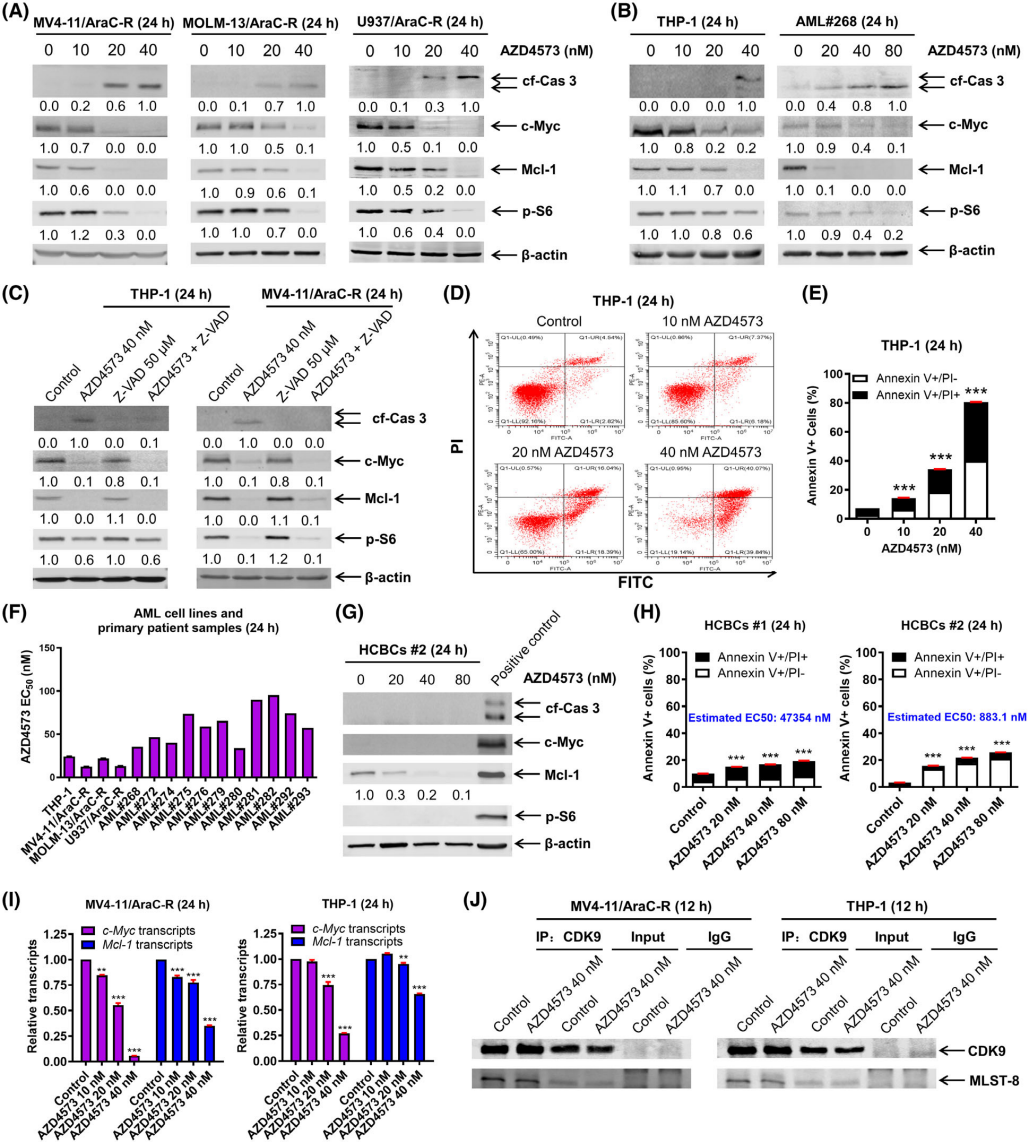
AZD4573 downregulates c‐MYC, MCL‐1, and p‐S6 in AraC‐resistant AML cells and induces cell death. (A, B) AraC‐resistant AML cell lines and primary patient sample AML#268 were treated with AZD4573 for 24 h. Cleaved caspase 3 (cf‐Cas 3), c‐MYC, MCL‐1, p‐S6, and β‐actin protein levels were determined by western blot analyses of whole cell lysates. Densitometry measurements normalized to β‐actin and then compared to control are shown below the corresponding blot. (C) THP‐1 and MV4‐11/AraC‐R cells were treated with vehicle control, AZD4573, Z‐VAD‐FMK (Z‐VAD), or in combination for 24 h and whole cell lysates were subjected to western blot analyses. Densitometry measurements normalized to β‐actin and then compared to control are shown below the corresponding blot. (D, E) THP‐1 cells were treated with AZD4573 for 24 h and then subjected to annexin V‐FITC/PI staining and flow cytometry analyses. Representative dot plots are shown in panel D. Results are graphed as mean ± SEM from one experiment in triplicate. ***P < 0.001. One‐way ANOVA followed by Bonferroni's *post hoc* test was used to determine statistical significance. (F) AML cell lines and primary AML patient samples were treated with AZD4573 for 24 h and then subjected to annexin V‐FITC/PI staining and flow cytometry analyses. EC_50_s from one experiment in triplicate are plotted. (G) Human cord blood cells (HCBCs) were treated with AZD4573 or apoptosis‐inducing positive control for 24 h. Whole cell lysates were subjected to western blot analyses. Densitometry measurements of MCL‐1 protein were normalized to β‐actin and then compared to control. (H) Two different HCBCs were treated with AZD4573 for 24 h and then subjected to annexin V‐FITC/PI staining and flow cytometry analyses. Results from one experiment in triplicate are graphed as mean ± SEM. ***P < 0.001 compared to control (one‐way ANOVA followed by Bonferroni's *post hoc* test). (I) MV4‐11/AraC‐R and THP‐1 cells were treated with AZD4573 for 24 h. Total RNA was isolated, and then, *c‐MYC* and *MCL‐1* transcripts were measured by real‐time RT‐PCR. These experiments were performed once in quadruplicate; displayed data are graphed as mean ± SEM. **P < 0.01 and ***P < 0.001 compared to control (one‐way ANOVA followed by Bonferroni's *post hoc* test). (J) MV4‐11/AraC‐R and THP‐1 cells were treated with AZD4573 for 12 h. CDK9 was immunoprecipitated from whole cell lysates. Western blots were probed with anti‐CDK9 and ‐mLST8 antibodies. This experiment was performed two independent times; representative blots are shown.

CDK9 plays a key role in the transcription of *Mcl‐1* and *c‐MYC*. AZD4573 is a novel CDK9 inhibitor that has shown promising activity in AML cells. Thus, we tested AZD4573 against our AraC‐resistant AML cells. AZD4573 treatment increased cleaved caspase 3 and downregulated c‐MYC, MCL‐1, and p‐S6 in cells with acquired resistance to AraC (Fig. 2A), and in the inherently AraC‐resistant AML cell line THP‐1 and primary patient sample AML#268 (Fig. 2B, inherent resistance to AraC was reported in reference [11]). Treatment with the pan‐caspase inhibitor Z‐VAD‐FMK could not rescue c‐MYC, MCL‐1, and p‐S6, demonstrating that downregulation was not due to caspase activation (Fig. 2C). AZD4573 treatment induced concentration‐dependent induction of cell death in THP‐1 (Fig. 2D,E). EC_50_s for THP‐1, MV4‐11/AraC‐R, MOLM‐13/AraC‐R, and U937/AraC‐R ranged from 12.5 to 24.4 nm, while those for the primary patient samples ranged from 33.9 to 95.4 nm (Fig. 2F). Importantly, normal human cord blood cells treated with AZD4573 showed no obvious increase in caspase 3 cleavage and no detectable c‐MYC or p‐S6. While there was a decrease in MCL‐1 protein levels, the lack of increased caspase 3 cleavage and minimal induction of cell death (Fig. 2G,H) indicates a potential therapeutic window. Relative transcript levels of *c‐MYC* and *MCL‐1* decreased with increasing concentrations of AZD4573 (Fig. 2I). Further, c‐MYC and MCL‐1 protein half‐life were unaffected by AZD4573 treatment (Fig. S2). CDK9 has been reported to bind to the mTOR complex scaffold protein mLST8 and control mRNA translation through phosphorylation of S6 [20]. Thus, we performed immunoprecipitation of CDK9 and probed for mLST8. However, AZD4573 treatment had no obvious effect on CDK9 binding to mLST8 (Fig. 2J). Taken together, these results suggest that the downregulation of c‐MYC and MCL‐1 is due to transcriptional regulation, and suppression of p‐S6 was not due to dissociation of CDK9 from the mTORC1 complex.

### 3.3 AZD4573 enhances the antileukemic activity of VEN + AZA against AraC‐resistant AML cells

**Fig. 3:**
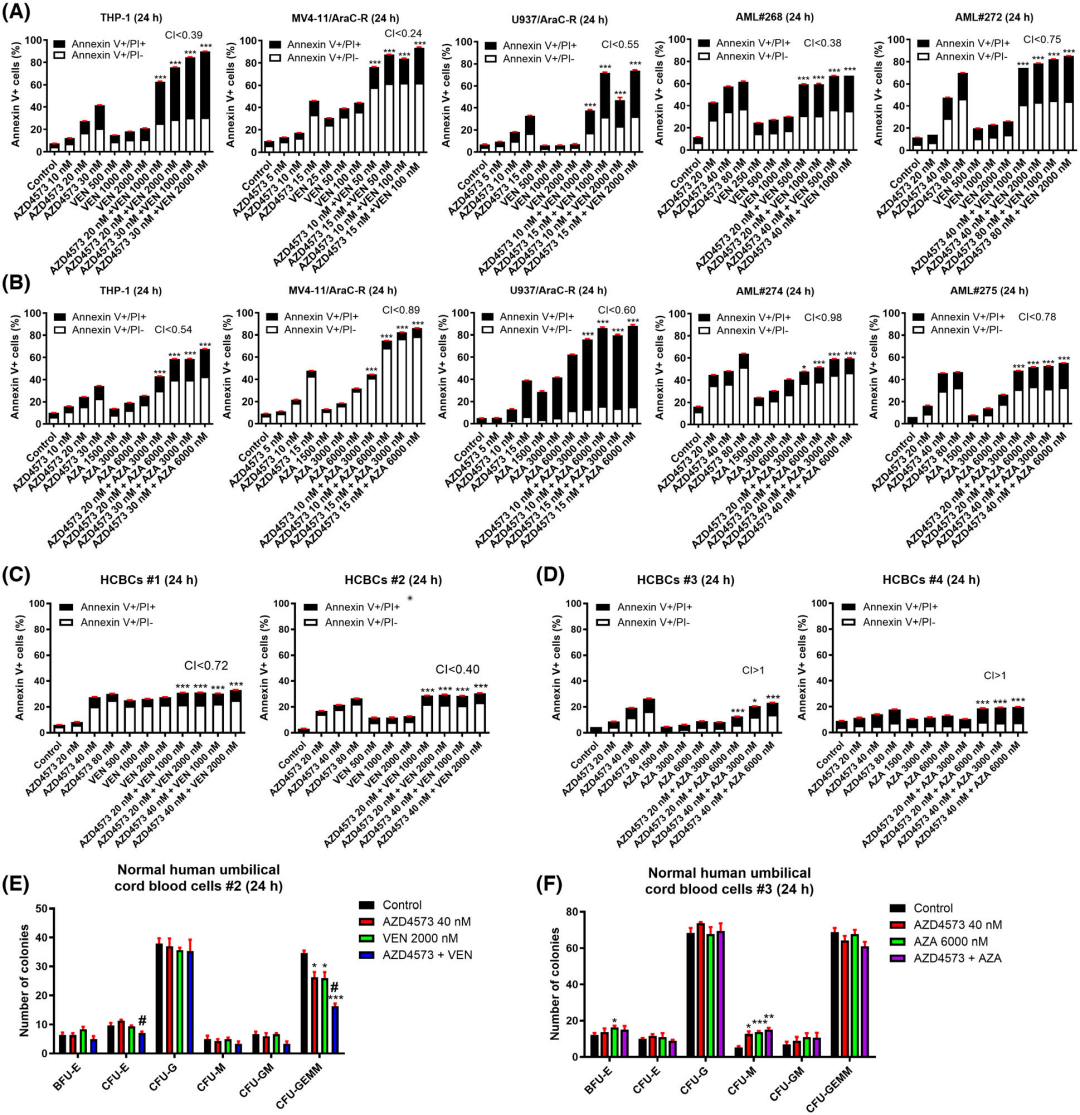
AZD4573 enhances the antileukemic activity of VEN and AZA against AraC‐resistant AML cells. (A, B) AML cell lines were treated with AZD4573, VEN, AZD4573 + VEN, AZA, or AZD4573 + AZA for 24 h and then subjected to annexin V‐FITC/PI staining and flow cytometry analyses. Combination index (CI) values were calculated using compusyn software. CI < 1.00 indicates synergy, CI > 1.00 indicates antagonism, and CI = 1.00 indicates additivity. These experiments were performed once in triplicate. Results are graphed as mean ± SEM. *P < 0.05 and ***P < 0.001 compared to control and all corresponding single‐drug treatments (one‐way ANOVA followed by Bonferroni's *post hoc* test). (C, D) Human cord blood cells (HCBCs) were treated with AZD4573, VEN, AZA, AZD4573 + VEN, or AZD4573 + AZA for 24 h and then subjected to annexin V‐FITC/PI staining and flow cytometry analyses. CI values were calculated using compusyn software. This experiment was performed once in triplicate. Results are graphed as mean ± SEM. *P < 0.05 and ***P < 0.001 compared to control and all corresponding single‐drug treatments (one‐way ANOVA followed by Bonferroni's *post hoc* test). (E, F) HCBCs were treated with vehicle control, AZD4573, VEN, AZA, AZD4573 + VEN, or AZD4573 + AZA for 24 h, washed, and then plated in methylcellulose. The number of erythroid and myeloid colonies was counted 10–14 days later. This experiment was performed once in triplicate. Data are presented as mean ± SEM. *P < 0.05, **P < 0.01, and ***P < 0.001 compared to vehicle control, while ^#^ *P* < 0.05 compared to all corresponding single‐drug treatments (one‐way ANOVA followed by Bonferroni's *post hoc* test).

Downregulation of c‐MYC and MCL‐1 has been shown to enhance the activity of VEN [9, 43, 44]. Thus, we next tested the combination of AZD4573 and VEN. AZD4573 synergizes with VEN to induce cell death in AraC‐resistant AML cell lines and primary patient samples (Fig. 3A). Reduction of c‐MYC has been shown to enhance the antileukemic activity of AZA; thus, we treated AraC‐resistant cells with AZA + AZD4573, which resulted in synergistic induction of cell death (Fig. 3B). Importantly, while AZD4573 synergizes with VEN in normal cord blood cells, the magnitude is substantially lower than in AML cells (Fig. 3C). Further, the combination of AZD4573 and AZA showed antagonism in these normal blood cells (CI > 1.0, Fig. 3D). Normal human umbilical cord blood cells treated with AZD4573 and VEN showed a small yet significant decrease of CFU‐E (erythroid colony‐forming units) and CFU‐GEMM (granulocyte, erythrocyte, macrophage, megakaryocyte colony‐forming units; Fig. 3E), while those treated with AZD4573 and AZA showed no significant decrease in colonies (Fig. 3F).

**Fig. 4:**
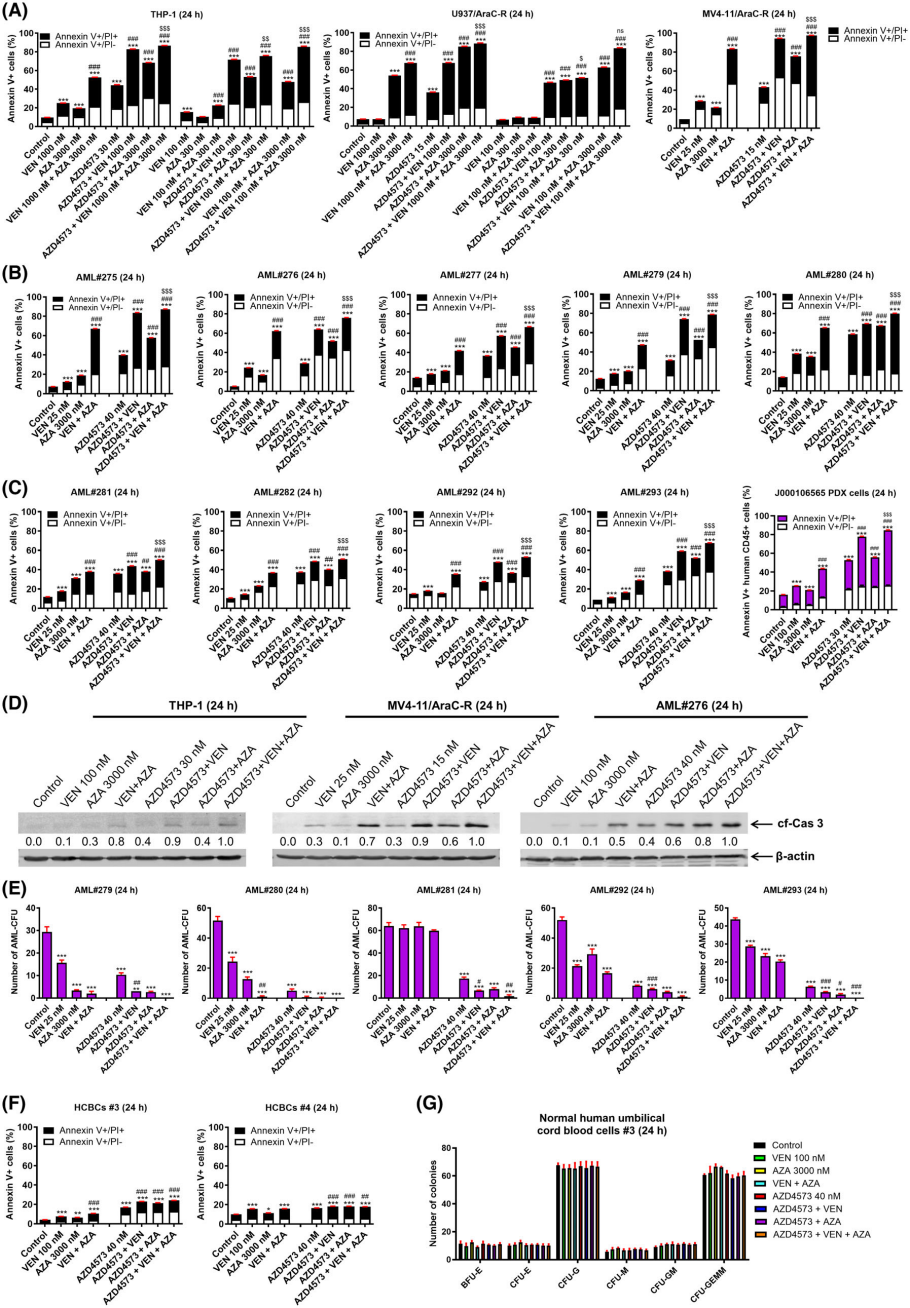
AZD4573 enhances the antileukemic activity of the combination of VEN and AZA against AraC‐resistant AML cells. (A–C) AML cell lines, primary AML patient samples, or J000106565 PDX cells were treated with vehicle control, VEN, AZA, AZD4573, or in the indicated combinations for 24 h and then subjected to annexin V‐FITC/PI staining and flow cytometry analyses. These experiments were performed once in triplicate. Results are graphed as mean ± SEM. ***P < 0.001 compared to vehicle control, ^##^ *P* < 0.01 and ^###^ *P* < 0.001 compared to all corresponding single‐drug treatments, ^$^ *P* < 0.05, ^$$^ *P* < 0.01, ^$$$^ *P* < 0.001, and ns indicates not significant compared to all corresponding two‐drug combinations (one‐way ANOVA followed by Bonferroni's *post hoc* test). (D) THP‐1, MV4‐11/AraC‐R, and AML#276 cells were treated with vehicle control, VEN, AZA, VEN + AZA, AZD4573, AZD4573 + VEN, AZD4573 + AZA, or AZD4573 + VEN + AZA for 24 h. Whole cell lysates were subjected to western blot analyses. Densitometry measurements of cleaved caspase 3 (cf‐Cas 3) were normalized to β‐actin and then compared to control. These experiments were performed once. (E) Primary AML patient samples were treated with vehicle control, VEN, AZA, VEN + AZA, AZD4573, AZD4573 + VEN, AZD4573 + AZA, or AZD4573 + VEN + AZA for 24 h, washed, and then plated in methylcellulose. The number of leukemic colonies (AML‐CFUs) were counted 10–14 days later. Experiment was performed once in triplicate. Data are presented as mean ± SEM. *** indicates *P* < 0.001 compared to vehicle control, while #, ##, and ### indicate *P* < 0.05, *P* < 0.01, and *P* < 0.001, respectively, compared to all corresponding single‐drug treatments (one‐way ANOVA followed by Bonferroni's *post hoc* test). (F) Human cord blood cells (HCBCs) were treated with vehicle control, VEN, AZA, VEN + AZA, AZD4573, AZD4573 + VEN, AZD4573 + AZA, or AZD4573 + VEN + AZA for 24 h and then subjected to annexin V‐FITC/PI staining and flow cytometry analyses. This experiment was performed once in triplicate. Data are presented as mean ± SEM. *, **, and *** indicate *P* < 0.05, *P* < 0.01, and *P* < 0.001, respectively, compared to vehicle control (one‐way ANOVA followed by Bonferroni's *post hoc* test). ## and ### indicate *P* < 0.01 and *P* < 0.001, respectively, compared to all corresponding single‐drug treatments (one‐way ANOVA followed by Bonferroni's *post hoc* test). (G) HCBCs were treated with vehicle control, VEN, AZA, VEN + AZA, AZD4573, AZD4573 + VEN, or AZD4573 + AZA or AZD4573 + VEN + AZA for 24 h, washed, and then plated in methylcellulose. The number of erythroid and myeloid colonies was counted 10–14 days later. Experiment was performed once in triplicate, and data are presented as mean ± SEM (one‐way ANOVA followed by Bonferroni's *post hoc* test).

Next, we tested the combination of AZD4573, VEN, and AZA in two AraC‐resistant AML cell lines, both relatively resistant to VEN. The three‐drug combination resulted in significant induction of cell death compared to single‐ and two‐drug combinations (Fig. 4A). Reducing both the VEN and AZA concentrations 10‐fold resulted in significant induction of cell death compared to the matching single‐drug treatments. However, the magnitude of the three‐drug combinations was notably lower. Interestingly, reduction of VEN by 10‐fold (100 nm), but not AZA, resulted in similar levels of cell death (85.4% vs 86.5% for THP‐1 cells and 83.3% vs 88.4% for U937/AraC‐R cells). In MV4‐11/AraC‐R, which is relatively sensitive to VEN, the three‐drug combination showed significantly increased cell death compared to all the two‐drug combinations. The three‐drug combination also showed a significant increase in cell death of primary AML patient samples from both newly diagnosed and relapsed AML patients, as well PDX cells from a relapsed AML patient (Fig. 4B,C). Induction of annexin V positivity was accompanied by cleavage of caspase 3 (Fig. 4D), indicating induction of apoptosis. Colony formation assays revealed a significant reduction of colonies following treatment with single‐drug treatments and two‐drug treatments compared to control in 4 of the 5 patient samples (Fig. 4E). Colonies from two‐drug treatments were equivalent or significantly reduced compared to single‐drug treatments. For AML#292 and AML#293, the three‐drug combination significantly reduced the number of colonies compared to control and single‐drug treatments. While the three‐drug combinations were not found to be significantly lower than the two‐drug combinations, patient samples AML#279, AML#280, and AML#293 had zero colonies on all three replicate plates. Importantly, treatment of human cord blood cells resulted in minimal induction of cell death and did not significantly affect colony formation (Fig. 4F,G), indicating a potential therapeutic window.

### 3.4 Downregulation of c‐MYC and MCL‐1 by AZD4573 enhances VEN + AZA‐induced AML cell death

**Fig. 5:**
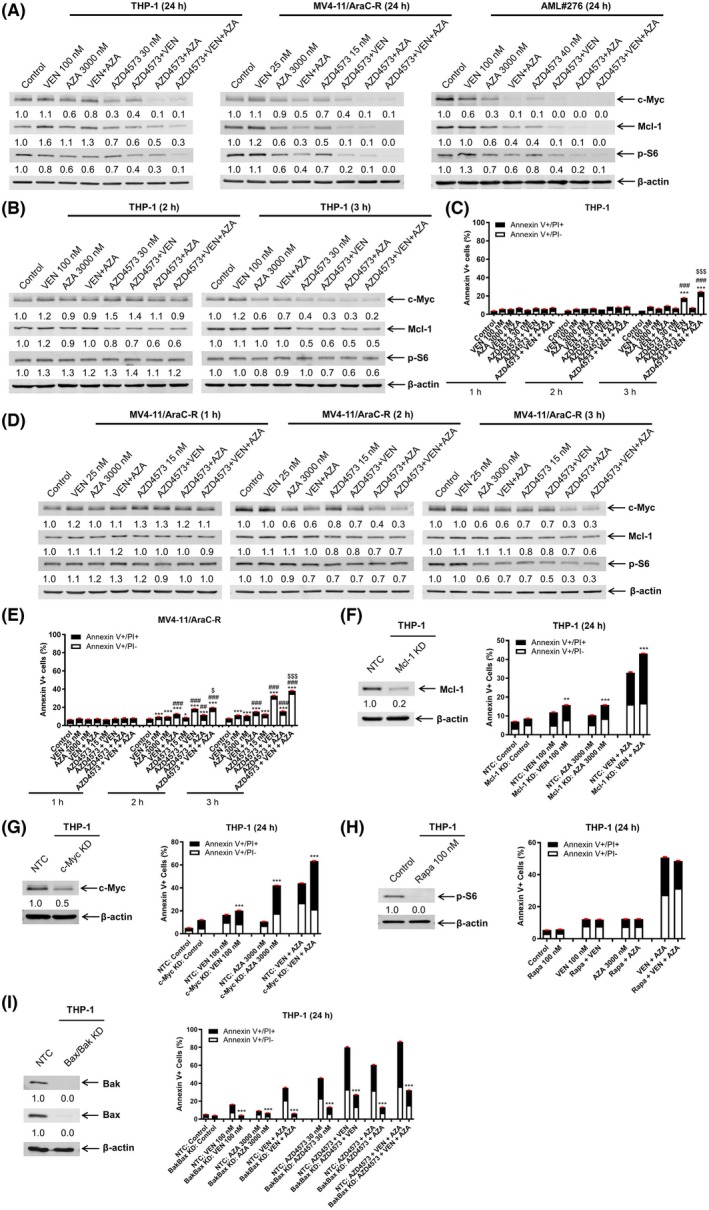
Downregulation of c‐MYC and MCL‐1 by AZD4573 enhances VEN + AZA‐induced AML cell death. (A) AML cells were treated with vehicle control, VEN, AZA, VEN + AZA, AZD4573, AZD4573 + VEN, AZD4573 + AZA, or AZD4573 + VEN + AZA for 24 h and then subjected to western blot analyses. Densitometry measurements normalized to β‐actin and then compared to control are shown below the corresponding blot. These experiments were performed once. (B, C) THP‐1 cells were treated with vehicle control, VEN, AZA, VEN + AZA, AZD4573, AZD4573 + VEN, AZD4573 + AZA, or AZD4573 + VEN + AZA for 1, 2, or 3 h. Whole cell lysates were subjected to western blot analyses (panel B). Densitometry measurements normalized to β‐Actin and then compared to control are shown below the corresponding blot (this experiment was performed once). Cells were subjected to annexin V‐FITC/PI staining and flow cytometry analyses (panel C). ***P < 0.001 compared to control, ^###^ *P* < 0.001 compared to all corresponding single‐drug treatments, ^$$$^ *P* < 0.001 compared to all corresponding two‐drug combinations (one‐way ANOVA followed by Bonferroni's *post hoc* test). Flow cytometry analysis was performed once in triplicate. (D, E) MV4‐11/AraC‐R cells were treated with vehicle control, VEN, AZA, VEN + AZA, AZD4573, AZD4573 + VEN, AZD4573 + AZA, or AZD4573 + VEN + AZA for 1, 2, or 3 h. Whole cell lysates were subjected to western blot analyses (panel D). Densitometry measurements normalized to β‐actin and then compared to control are shown below the corresponding blot (this experiment was performed once). Cells were subjected to annexin V‐FITC/PI staining and flow cytometry analyses (panel E). *P < 0.05 and ***P < 0.001 compared to control, ^##^ *P* < 0.01 and ^###^ *P* < 0.001 compared to all corresponding single‐drug treatments, and ^$^ *P* < 0.05 and ^$$$^ *P* < 0.001 compared to all corresponding two‐drug combinations (one‐way ANOVA followed by Bonferroni's *post hoc* test). Flow cytometry analysis was performed once in triplicate. (F, G) CRISPR knockdown (KD) of MCL‐1, c‐MYC, or nontarget control (NTC) was performed in THP‐1 cells. Whole cell lysates were subjected to western blotting and probed with anti‐MCL‐1, anti‐c‐MYC and anti‐β‐actin antibodies. Relative densitometry measurements, normalized to β‐actin and compared to the control, are shown (experiment repeated in triplicate independently, displayed data are from one representative experiment). Cells were treated with vehicle control, VEN, AZA, or VEN + AZA for 24 h and then subjected to annexin V‐FITC/PI staining and flow cytometry analyses. **P < 0.01 and ***P < 0.001 compared to NTC under the same treatment condition (unpaired *t*‐test). Flow cytometry analysis was performed once in triplicate. (H) THP‐1 cells were treated with rapamycin, VEN, AZA, VEN + AZA, or rapamycin + VEN + AZA for 24 h and then subjected to annexin V‐FITC/PI staining and flow cytometry analyses (western blot was repeated in triplicate independently, displayed data are from one representative experiment). Vehicle control and rapamycin‐treated cells were subjected to western blotting and probed with anti‐p‐S6 and ‐β‐actin antibodies. Relative densitometry measurements, normalized to β‐actin and compared to the control, are shown. Flow cytometry analysis was performed once in triplicate. Unpaired *t*‐test was used to determine statistical significance. (I) Double knockdown of *Bak* and *Bax* was performed in THP‐1 cells. Nontarget control (NTC) shRNA was used as the control. Whole cell lysates were subjected to western blotting (western blot was repeated in triplicate independently (*n* = 3), displayed data are from one representative experiment). The fold changes for the densitometry measurements, normalized to β‐actin and compared to NTC, are indicated below the corresponding blot. The knockdown cells were treated with vehicle control, VEN, AZA, VEN + AZA, AZD4573, AZD4573 + VEN, AZD4573 + AZA, or AZD4573 + VEN + AZA for 24 h and then subjected to annexin V/PI staining and flow cytometry analysis. Flow cytometry analysis was performed once in triplicate. ***P < 0.001 compared to the NTC under the same treatment conditions (unpaired *t*‐test). Panels C, E–I, error bars are representative of standard error of the mean.

Next, we determined the effects of VEN, AZA, and AZD4573, alone and in combination, on c‐MYC, MCL‐1, and p‐S6 protein levels. Treatment with VEN alone (24 h) largely did not alter MCL‐1 levels in MV4‐11/AraC‐R and AML#276 cells but did increase it in THP‐1, consistent with our previous publications [9, 45] (Fig. 5A). Treatment with AZA for 24 h resulted in a decrease of p‐S6, which was maintained when VEN was added to the treatment (Fig. 5A). VEN + AZA treatment reduced c‐MYC, MCL‐1, and p‐S6 in MV4‐11/AraC‐R and AML#276 and c‐MYC and p‐S6 in THP‐1 cells. AZD4573 treatment substantially reduced c‐MYC, MCL‐1, and p‐S6 in the cell lines, while in AML#276 only c‐MYC and MCL‐1 decreased. AZD4573 combined with VEN or AZA showed similar or slightly more reduction of c‐MYC, MCL‐1, and p‐S6 compared to AZD4573 treatment alone. The three‐drug combination treatment resulted in very little to no detection of c‐MYC, MCL‐1, and p‐S6.

To determine if downregulation of these proteins occurs prior to cell death, THP‐1 cells were treated for a shorter time and protein level changes as well as annexin V positivity were determined. AZD4573 in combination with VEN, AZA, or VEN + AZA reduced MCL‐1 levels after 2 h of treatment, which was prior to induction of cell death (Fig. 5B,C). AZA treatment for 3 h resulted in decreased c‐MYC levels, which was maintained with the addition of VEN and occurred prior to induction of cell death. Combined AZD4573 and AZA treatment for 3 h decreased c‐MYC, MCL‐1, and p‐S6, which occurred prior to induction of cell death. AZD4573 in combination with VEN or in combination with VEN + AZA substantially decreased c‐MYC, MCL‐1, and p‐S6 after 3‐h treatment compared to AZA alone and was accompanied by significant induction of cell death, though the magnitude was small at 24.7% annexin V positivity. In MV4‐11/AraC‐R cells, 1‐h treatment had no substantial effect on c‐MYC, MCL‐1, or p‐S6 for any of the drug treatments (Fig. 5D). After 2‐h treatment, AZA decreased c‐MYC levels, while in combination with VEN it downregulated both c‐MYC and p‐S6. AZD4573 + VEN treatment (2 h) downregulated c‐MYC and p‐S6. AZD4573 + AZA treatment (2 h) reduced c‐MYC, MCL‐1, and p‐S6. AZD4573 in combination with VEN + AZA showed similar downregulation of c‐MYC, MCL‐1, and p‐S6 as treatment with AZD4573 + AZA, though more so when compared to AZA alone. While significant induction of cell death was detected with all 2‐h drug treatments, the magnitude was < 20%. Three‐hour treatment resulted in similar reduction of c‐MYC, MCL‐1, and p‐S6, and increased induction of cell death (Fig. 5E).

To determine if downregulation of MCL‐1 and c‐MYC by AZD4573 plays an important role in enhancing cell death induced by VEN + AZA, knockdown was performed in THP‐1 cells. Knockdown of MCL‐1 or c‐MYC significantly enhanced cell death induced by VEN, AZA, and VEN + AZA (Fig. 5F,G). However, inhibition of mTOR had no significant effect on cell death induced by VEN, AZA, or VEN + AZA (Fig. 5H). Bax/Bak double knockdown significantly reduced cell death induced by VEN, AZA, VEN + AZA, AZD4573, AZD4573 + VEN, AZD4573 + AZA, and AZD4573 + VEN + AZA demonstrating that these drugs induce cell death at least partially through induction of intrinsic apoptosis (Fig. 5I). Taken together, these results show the important role c‐MYC and MCL‐1 play in AZD4573 + VEN + AZA induced cell death, which occurs at least partially through the intrinsic apoptosis pathway.

### 3.5 Downregulation of mitochondrial metabolism plays a role in AZD4573 + venetoclax + azacitidine‐induced AML cell death

**Fig. 6:**
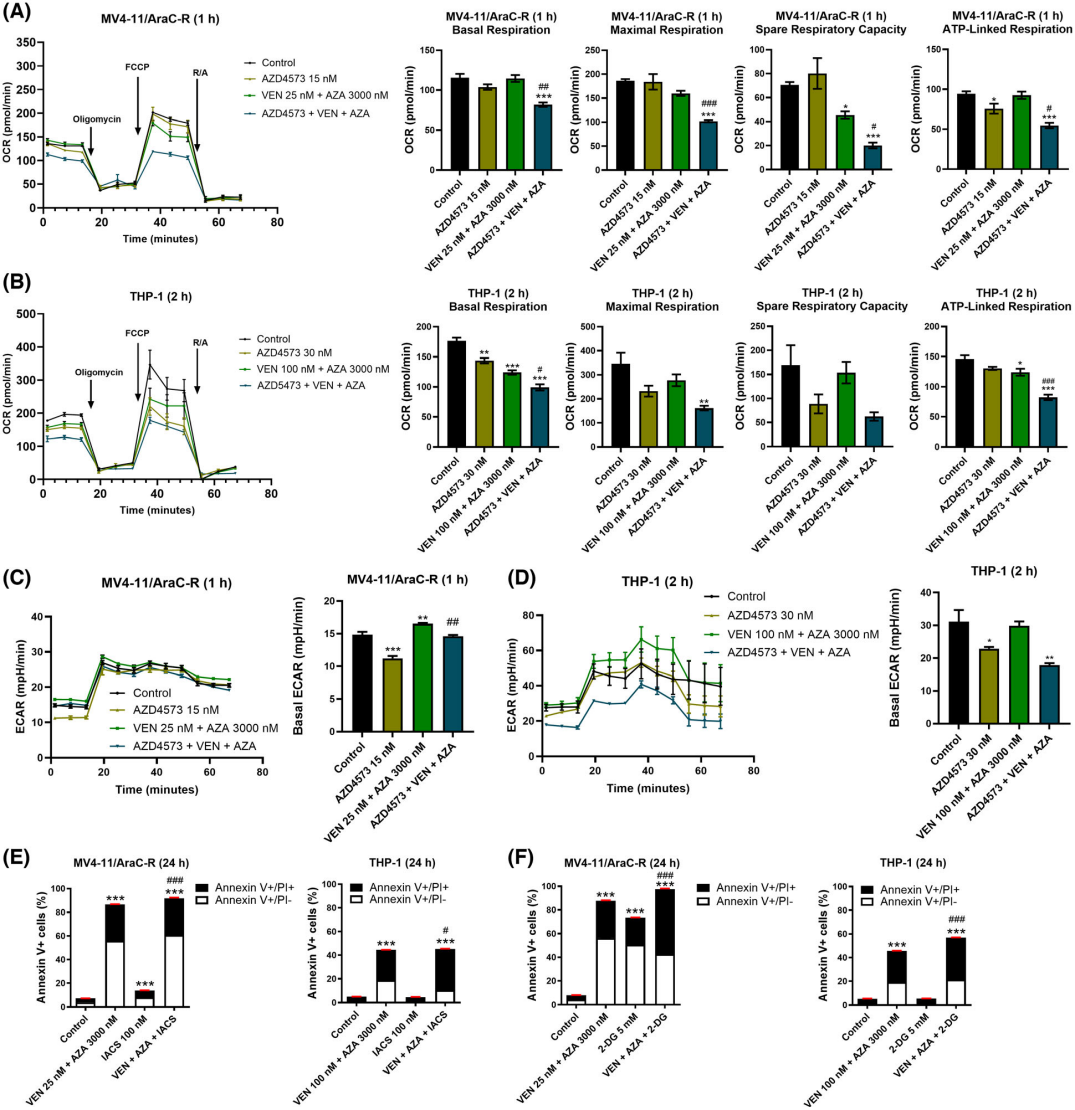
Suppression of cellular respiration plays a role in AZD4573 + VEN + AZA‐induced AML cell death. (A, B) MV4‐11/AraC‐R and THP‐1 cells were treated with AZD4573, VEN + AZA, AZD4573 + VEN + AZA for 1 or 2 h. Cells were then subjected to the Cell Mito Stress Test. The oxygen consumption rate (OCR) was measured under basal conditions, following inhibition of ATP synthase (with oligomycin), uncoupling of the electron transport chain (with FCCP), and inhibition of complexes I and III (with rotenone and antimycin A (R/A)). OCR plots are shown on the left, while results of basal respiration, maximal respiration, spare respiratory capacity, and ATP‐linked respiration are shown on the right. This experiment was performed once in quadruplicate. *P < 0.05, **P < 0.01, and ***P < 0.001 compared to vehicle control, while ^#^ *P* < 0.05, ^##^ *P* < 0.01, and ^###^ *P* < 0.001 compared to AZD4573 or VEN + AZA (one‐way ANOVA followed by Bonferroni's *post hoc* test). (C, D) MV4‐11/AraC‐R and THP‐1 cells were treated with AZD4573, VEN + AZA, AZD4573 + VEN + AZA for 1 or 2 h. Glycolysis was measured via extracellular acidification rate (ECAR; left panels) using a Seahorse Bioanalyzer. Basal ECAR is graphed on the right. This experiment was performed once in quadruplicate. *P < 0.05, **P < 0.01, ***P < 0.001 compared to vehicle control, while ^##^ *P* < 0.01 compared to AZD4573 and VEN + AZA (one‐way ANOVA followed by Bonferroni's *post hoc* test). (E) MV4‐11/AraC‐R and THP‐1 cells were treated with vehicle control, IACS‐010759 (IACS), VEN + AZA, or IACS + VEN + AZA for 24 h and then subjected to annexin V/PI staining and flow cytometry analysis. ***P < 0.001 compared to vehicle control, while ^#^ *P* < 0.05 and ^###^ *P* < 0.001 compared to IACS and VEN + AZA (one‐way ANOVA followed by Bonferroni's *post hoc* test). Flow cytometry analysis was performed once in triplicate. (F) MV4‐11/AraC‐R and THP‐1 cells were treated with vehicle control, 2‐deoxy‐d‐glucose (2‐DG), VEN + AZA, or 2‐DG + VEN + AZA for 24 h and then subjected to annexin V/PI staining and flow cytometry analysis. *** indicates *P* < 0.001 compared to vehicle control, while ### indicates *P* < 0.001 compared to 2‐DG and VEN + AZA (one‐way ANOVA followed by Bonferroni's *post hoc* test). Flow cytometry analysis was performed once in triplicate. For all panels, error bars are representative of standard error of the mean.

c‐MYC is an important regulator of oxidative phosphorylation and glycolysis. Thus, we measured the effects of AZD4573 alone and in combination with VEN + AZA on oxygen consumption rate (OCR) and extracellular acidification rate (ECAR), indicative of OXPHOS and glycolysis, respectively. The three‐drug combination significantly decreased basal respiration, maximal respiration, spare respiratory capacity, and ATP‐linked respiration in MV4‐11/AraC‐R cells when cells were treated for only 1 h (Fig. 6A). Similar results were obtained in THP‐1 cells, though the cells were treated for 2 h (Fig. 6B). In MV4‐11/AraC‐R cells, basal ECAR was significantly decreased following AZD4573 treatment, while VEN + AZA treatment significantly increased basal ECAR, and the addition of AZD4573 reverted basal ECAR levels to baseline (Fig. 6C). In THP‐1 cells, AZD4573 treatment significantly decreased basal ECAR, VEN + AZA had no significant effect on basal ECAR, and the three‐drug combination reduced basal ECAR to a similar level as AZD4573 treatment alone (Fig. 6D). To assess the role of OXPHOS suppression by AZD4573 in cell death induced by VEN + AZA, MV4‐11/AraC‐R, and THP‐1 cells were treated with mitochondrial complex 1 inhibitor IACS‐010759 alone and in combination with VEN + AZA. IACS‐010759 significantly enhanced cell death induced by VEN + AZA, though the enhancement was minimal (Fig. 6E). Treatment with glycolysis inhibitor 2‐DG significantly enhanced cell death induced by VEN + AZA (Fig. 6F). These results suggest that suppression of OXPHOS and glycolysis by AZD4573 also contributes to cell death induced by VEN + AZA in AraC‐resistant AML, though only to a small extent.

## 4 Discussion

In this study, we show that the CDK9 selective inhibitor, AZD4573, enhances the *in vitro* antileukemic activity of VEN, AZA, and VEN + AZA against AML cells with inherent or acquired AraC resistance but spares normal hematopoietic cells. Additionally, another CDK9 inhibitor, LDC000067, enhances the *in vitro* antileukemic activity of VEN, AZA, and VEN + AZA against AraC‐resistant AML cells (Fig. S3). These results are consistent with reports of cooperative antileukemic activity between CDK9 inhibition and VEN [9, 46, 47]. Further, we confirm results from our previous report [11], that MCL‐1 knockdown enhances VEN + AZA‐induced AML cell death (Fig. 5F). Carter et al [47] reported that AZD4573 downregulates MCL‐1 and synergizes with VEN in AML cells, including those resistant to VEN and VEN + decitabine. Importantly, our results build upon these findings and show that AZD4573 synergizes with VEN + AZA against AML cells resistant to AraC, suggesting that the combination may show efficacy against R/R AML postchemotherapy.

An interesting finding that we previously reported was that CDK9 inhibitors, voruciclib and flavopiridol, transiently downregulate MCL‐1 [9]. Furthermore, intermittent inhibition of CDK9 resumed downregulation of MCL‐1 and showed better enhancement when combined with VEN [9]. In this study, we did not directly investigate if AZD4573 also induces a transient downregulation of MCL‐1, though our results show that substantial downregulation of MCL‐1 protein is still detectable at 24 h. Additionally, Cidado et al showed downregulation of MCL‐1 protein after 2‐h treatment with AZD4573, which was further reduced at 4 h and maintained through 24 h [22], suggesting that MCL‐1 rebound may not occur with AZD4573 treatment or perhaps to a much lesser extent. Based on the important role MCL‐1 plays in enhancing VEN + AZA‐induced AML cell death, AZD4573 may have greater potential in combination with VEN + AZA than other CDK9 inhibitors that transiently downregulate MCL‐1, though further studies are warranted.

In addition to MCL‐1, AZD4573 treatment also downregulates c‐MYC and enhances VEN + AZA‐induced AML cell death (Fig. 5), which is consistent with our previous report [11]. In that study, we demonstrated that inhibition of c‐MYC significantly decreases both OXPHOS and glycolysis in AraC‐resistant AML cells [11]. Our results here also show a significant decrease in basal glycolysis (Fig. 6C,D), though AZD4573 treatment only significantly reduced OXPHOS in one of the two cell lines tested (Fig. 6A,B). While inhibition of mitochondrial complex I and glycolysis significantly enhanced AML cell death induced by VEN + AZA, the magnitude of the enhancement over VEN + AZA was minimal, suggesting a minor role in the mechanism of action against bulk AML cells. However, we speculate that the inhibition of OXPHOS could play a more substantial role in the antileukemic activity against chemotherapy‐resistant and persistent cells as they reportedly rely on OXPHOS for survival [48].

Recently, it was reported that reduction of the VEN dosage and duration in newly diagnosed AML patients treated with the combination of VEN + AZA maintained similar response and overall survival [49]. Our *in vitro* studies show that reduction of VEN concentration results in similar levels of cell death induced by the combination of VEN + AZA in AraC‐resistant AML cell lines (Fig. 4A). Additionally, our results show reduction of VEN when used in combination with AZD4573 + AZA may also be taken into consideration, supporting further preclinical studies to determine *in vivo* efficacy of the three‐drug combination and potential dose reduction of VEN. However, for the two‐drug combination of AZD4573 and VEN, our data indicate that reduction of VEN concentration may not result in as favorable results.

## 5 Conclusions

In conclusion, AZD4573 in combination with VEN + AZA shows promising *in vitro* antileukemic activity against AML cells resistant to AraC. While *in vivo* data are needed to support further development, interim results of a Phase 2a clinical trial suggest that the strategy of combining a CDK9 inhibitor (SLS009) with VEN + AZA is safe and feasible in AML patients R/R to VEN‐based combinations [50]. Our results support further investigation of the combination of AZD4573 with VEN + AZA for the treatment of AraC‐resistant AML, though preclinical *in vivo* studies are warranted to establish efficacy and address toxicity.

## Conflict of interest

The authors declare no conflict of interest.

## Author contributions

YW, JWT, and YG conceived and designed the project. SW, JZ, AAA, AG, JT, SL, JiY, and HE performed experiments. SW, JZ, AG, JT, SL, JiY, GW, HE, MH, LAP, JK, SHD, KW, JaY, YW, JWT, and YG analyzed the data. SW, JZ, AG, JT, SL, JiY, GW, HE, LAP, JK, SHD, KW, and YG visualized the data. HE wrote the original draft. SW, JZ, AAA, AG, JT, SL, JiY, GW, HE, MH, LAP, JK, SHD, KW, JiY, JaY, YW, JWT, and YG reviewed and edited the manuscript. All authors have read and agreed to the published version of the manuscript.

## Supporting information


**Fig. S1.** *In vitro* AraC sensitivity of primary AML patient samples.
**Fig. S2.** AZA4573 treatment does not affect c‐MYC and MCL‐1 protein half‐life.
**Fig. S3.** LDC000067 enhances the antileukemic activity of the combination of VEN and AZA against AraC‐resistant AML.
**Table S1.** Patient characteristics of the primary AML patient samples used in this study.

**Data S1.** Full‐length western blots.

## Data Availability

The data that support the findings of this study are available from the corresponding author (Yubin Ge, gey@karmanos.org) upon reasonable request.
