# Supplementary material for: Inhibition of CDK9 enhances AML cell death induced by combined venetoclax and azacitidine
Source: Mol Oncol. 2025 Sep 16;20(2):555–72. doi: 10.1002/1878-0261.70124 (PMC12936417; doi:10.1002/1878-0261.70124)
Supplement: Supplementary file 1 — Fig. S1. In vitro AraC sensitivity of primary AML patient samples. Fig. S2. AZA4573 treatment does not affect c‐MYC and MCL‐1 protein half‐life. Fig. S3. LDC000067 enhances the antileukemic activity of the combination of VEN and AZA against AraC‐resistant AML. Table S1. Patient characteristics of the primary AML patient samples used in this study. [file MOL2-20-555-s001.docx]

**Inhibition of CDK9 enhances AML cell death induced by combined venetoclax and azacitidine**

Shuangshuang Wu^1^, Jianlei Zhao^2,3^, Aaban Asfar Azmi^3^, Avanti Gupte^4,5^, Jenna Thibodeau^6^, Shuang Liu^7^, Jinli Yang^7^, Guan Wang^7^, Holly Edwards^2,3^, Lisa A. Polin^2,3^, Juiwanna Kushner^2,3^, Sijana H. Dzinic^2,3^, Kathryn White^2,3^, Julie Boerner^2,3^, Maik Hüttemann^6,8^, Jay Yang^2,3^, Yue Wang^1,^*, Jeffrey W. Taub^4,5,6,9,^*, and Yubin Ge^2,3,6,^*

^1^Department of Pediatric Hematology, Children’s Medical Center, The First Hospital of Jilin University, Changchun, P.R. China

^2^Department of Oncology, Wayne State University School of Medicine, Detroit, MI, USA

^3^Molecular Therapeutics Program, Barbara Ann Karmanos Cancer Institute, Wayne State University School of Medicine, Detroit, MI, USA

^4^Division of Pediatric Hematology/Oncology, Children’s Hospital of Michigan, Detroit, MI, USA

^5^Department of Pediatrics, Central Michigan University College of Medicine, Mt. Pleasant, MI, USA

^6^Cancer Biology Graduate Program, Wayne State University School of Medicine, Detroit, MI, USA

^7^National Engineering Laboratory for AIDS Vaccine, Key Laboratory for Molecular Enzymology and Engineering, the Ministry of Education, School of Life Sciences, Jilin University, Changchun, P. R. China

^8^Center for Molecular Medicine and Genetics, Wayne State University School of Medicine, Detroit, MI, USA

^9^Department of Pediatrics, Wayne State University School of Medicine, Detroit, MI, USA

*Corresponding authors:

Yubin Ge

Department of Oncology

Wayne State University School of Medicine

421 E. Canfield, Detroit, MI 48201

Email: [gey@karmanos.org](mailto:gey@karmanos.org)

Jeffrey Taub

Division of Pediatric Hematology and Oncology

Children’s Hospital of Michigan

3901 Beaubien St., Detroit, MI 48201

Email: [jtaub@med.wayne.edu](mailto:jtaub@med.wayne.edu)

Yue Wang

Department of Pediatric Hematology

Children’s Medical Center

The First Hospital of Jilin University

2699 Qianjin Street, Changchun, Jilin, P.R. China 130021

Email: [wang_yue@jlu.edu.cn](mailto:wang_yue@jlu.edu.cn)


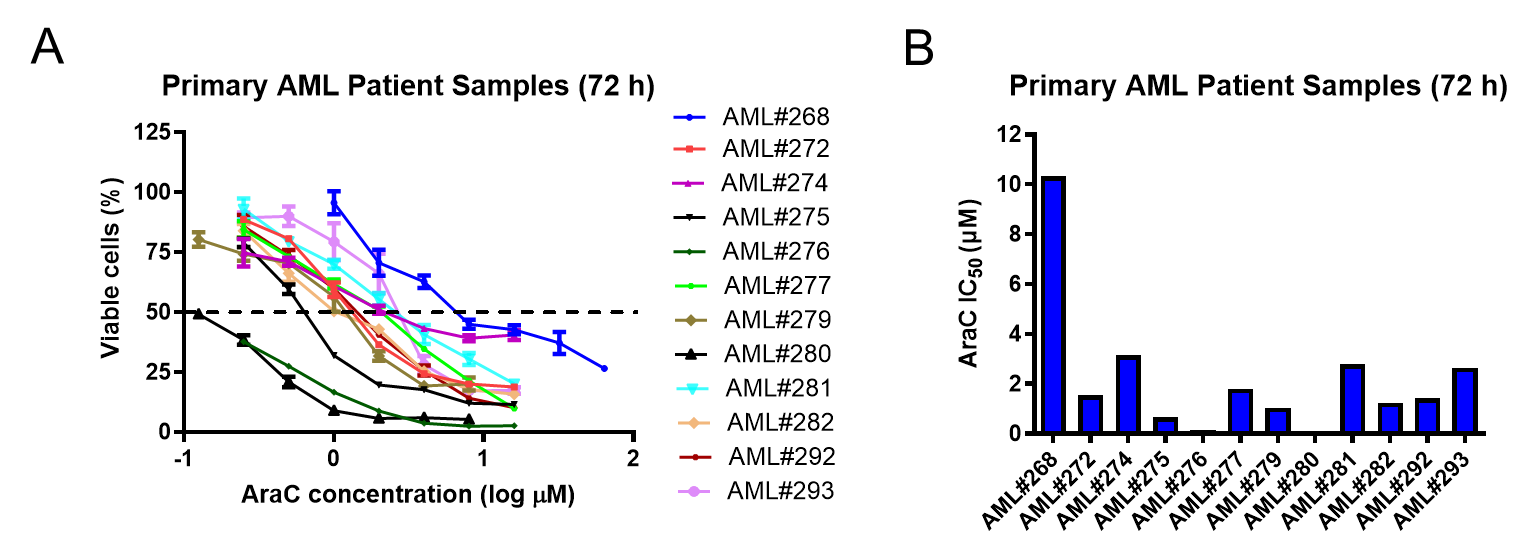


**Figure S1.** ***In vitro* AraC sensitivity of primary AML patient samples.** Primary AML patient samples were treated with variable concentrations of cytarabine (AraC) for 72 h and MTT reagent was used to determine viable cells. This experiment was performed once in triplicate due to limited sample. Percent of viable cells relative to no drug treatment control is shown in panel A. The AraC IC_50_ values were calculated as the concentration of drugs that inhibit 50 % of viable cells compared to no drug treatment control (panel B). Error bars are representative of standard error of the mean.


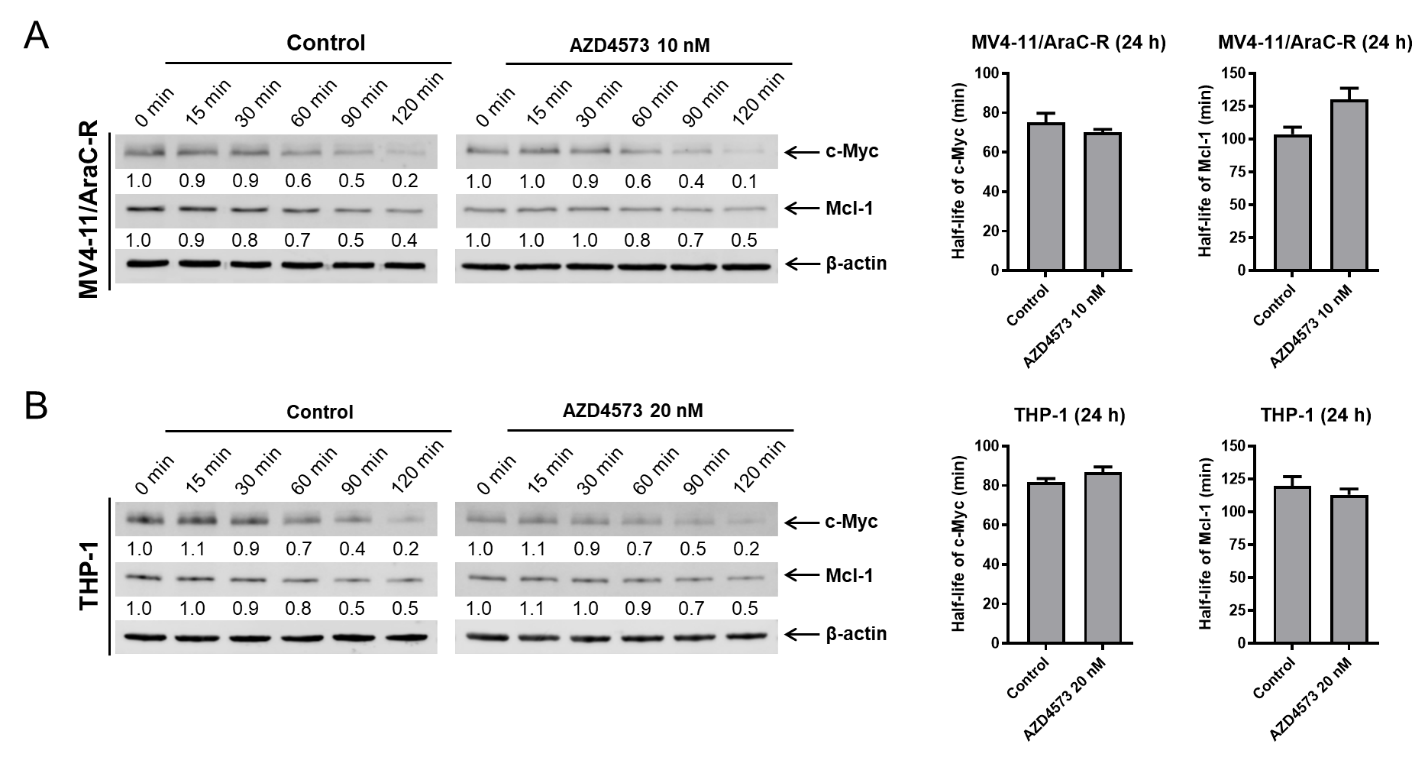


**Figure S2.** **AZD4573 treatment does not affect c-MYC and MCL-1 protein half-life.** AML cells were treated with vehicle control or AZD4573 for 24 h, washed and then treated with 10 μg/mL cycloheximide for up to 2 h. Western blots were probed with anti-c-Myc, -Mcl-1 or -β-actin antibody. Relative densitometry measurements, normalized to β-actin and compared to 0 min, are shown. Representative blots are shown in the left panels. c-Myc and Mcl-1 protein half-life were determined using the densitometry measurements from 3 independent experiments and are shown in the right panels. Error bars are representative of standard error of the mean.


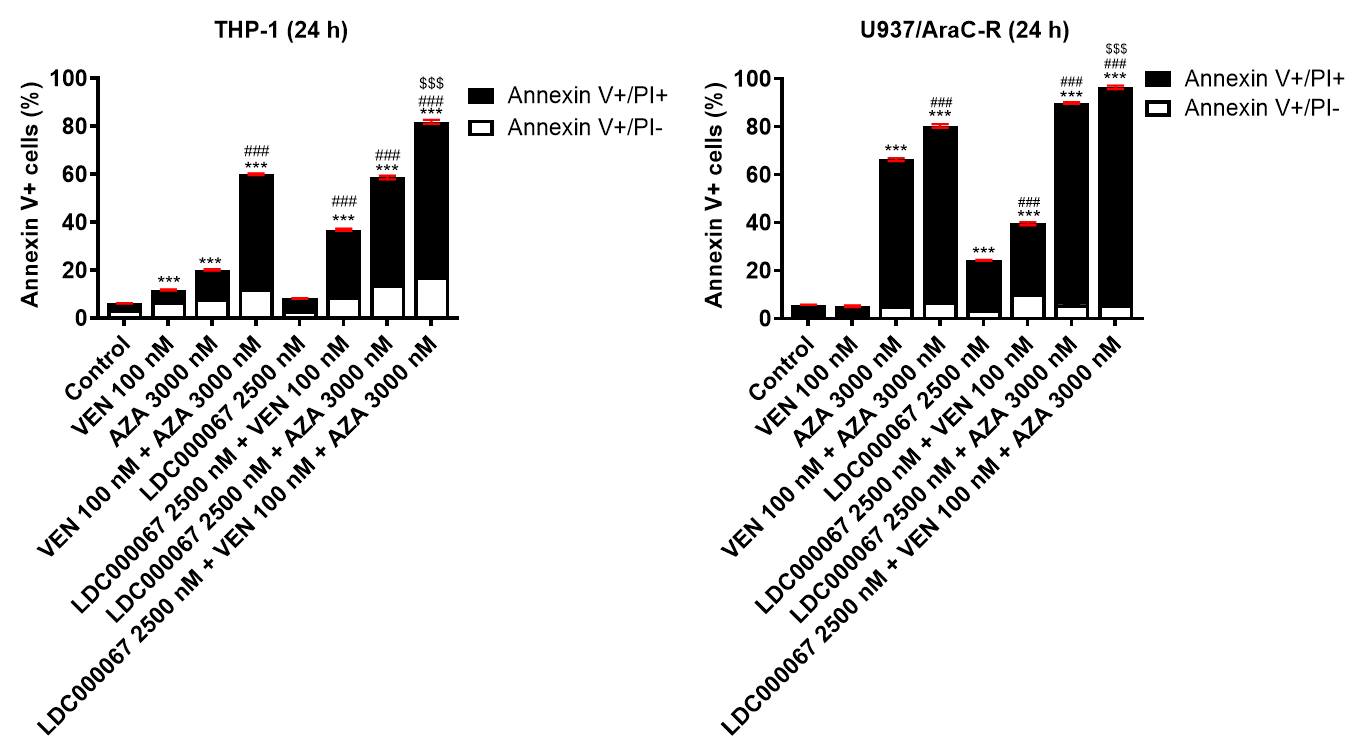


**Figure S3. LDC000067 enhances the antileukemic activity of the combination of VEN and AZA against AraC-resistant AML cells.** THP-1 and U937/AraC-R cells were treated with vehicle control, VEN, AZA, LDC000067, or in the indicated combinations for 24 h and then subjected to annexin V-FITC/PI staining and flow cytometry analyses. Results are graphed as mean ± SEM. *** indicates p<0.001 compared to vehicle control, ### indicates p<0.001 compared to all corresponding single drug treatments, $$$ indicates p<0.001 compared to all corresponding two-drug combinations (one-way ANOVA followed by Bonferroni’s post hoc test). Experiment was performed once in triplicate. Error bars are representative of standard error of the mean.

**Table S1. Patient characteristics of the primary AML patient samples used in this study.**

| **Patient** | **Gender** | **Age (year)** | **Disease status** | **FAB subtype** | **Cytogenetics** | **Blast purity (%)** | **Gene mutation/Fusion gene** |
| --- | --- | --- | --- | --- | --- | --- | --- |
| AML#268 | Female | 55 | Newly diagnosed | AML | 46,XX,t(9;22)(q34;q11.2)/46,XX | 67.5 | *BCR/ABL1(P190), CSMD1, DNMT3A* |
| AML#272 | Male | 49 | Newly diagnosed | AML | 45, X,-Y,t(8;21)(q22;q22)del(11)(q13q23)/  46, XY | 64.5 | *AML1-ETO, RUNX1-RUNX1T1,IDH, KMT2C, ZBTB7A, POT1* |
| AML#274 | Female | 43 | Newly diagnosed | AML | 47,XX，t(8:21)(q22:q22),+15 | 90.5 | *c-KIT* |
| AML#275 | Female | 35 | Newly diagnosed | M3 | 46,XX,t(15;17)(q24;q21)/46,XX | 90 | *PML/RARa, FLT3-*ITD |
| AML#276 | Male | 56 | Newly diagnosed | AML | 46, XY | 77.5 | *FLT3-*ITD*, CEBPA* |
| AML#277 | Male | 57 | Newly diagnosed | M4/M5 | 46,XY,inv(16)(p13q22)/46,XY | 75.5 | *CBFβ-MYH11, KIT, NRAS, DNMT3A* |
| AML#279 | Male | 48 | Newly diagnosed | M4/M5 | 46,XY | 97 | *FLT3-*ITD*, TET2* |
| AML#280 | Male | 18 | Newly diagnosed | AML | 46,XY,del(9)(q13q22)/46,XY | 80.5 | *CEBPA* |
| AML#281 | Female | 57 | Relapsed | M2 | 46,XX | 66.5 | *WT1* |
| AML#282 | Male | 39 | Relapsed | M4/M5 | 46,XY | 84.5 | *NPM1, NRAS* |
| AML#292 | Male | 28 | Relapsed | AML | 46,XY | 56.5 | *KMT2A-MLLT4, SF3B1, WT1* |
| AML#293 | Female | 54 | Relapsed | AML | 46,XX,del(11)(p13) | 82.5 | *FLT3-ITD, NPM1, WT1* |
