# Supplementary material for: Inhibition of CDK9 enhances AML cell death induced by combined venetoclax and azacitidine
Source: Mol Oncol. 2025 Sep 16;20(2):555–72. doi: 10.1002/1878-0261.70124 (PMC12936417; doi:10.1002/1878-0261.70124)
Supplement: Supplementary file 2 — Data S1. Full‐length western blots. [file MOL2-20-555-s002.pdf]

Inhibition of CDK9 enhances AML cell death induced by combined venetoclax and azacitidine

Shuangshuang Wu[1], Jianlei Zhao[2,3], Aaban Asfar Azmi[3], Avanti Gupte[4,5], Jenna Thibodeau[6], Shuang Liu[7], Jinli Yang[7], Guan Wang[7], Holly Edwards[2,3], Lisa A. Polin[2,3], Juiwanna Kushner[2,3], Sijana H. Dzinic[2,3], Kathryn White[2,3], Julie Boerner[2,3], Maik Hüttemann[6,8], Jay Yang[2,3], Yue Wang[1,*], Jeffrey W. Taub[4,5,6,9,*], and Yubin Ge[2,3,6,*]

[1]Department of Pediatric Hematology, Children's Medical Center, The First Hospital of Jilin University, Changchun, P.R. China
[2]Department of Oncology, Wayne State University School of Medicine, Detroit, MI, USA
[3]Molecular Therapeutics Program, Barbara Ann Karmanos Cancer Institute, Wayne State University School of Medicine, Detroit, MI, USA
[4]Division of Pediatric Hematology/Oncology, Children's Hospital of Michigan, Detroit, MI, USA
[5]Department of Pediatrics, Central Michigan University College of Medicine, Mt. Pleasant, MI, USA
[6]Cancer Biology Graduate Program, Wayne State University School of Medicine, Detroit, MI, USA
[7]National Engineering Laboratory for AIDS Vaccine, Key Laboratory for Molecular Enzymology and Engineering, the Ministry of Education, School of Life Sciences, Jilin University, Changchun, P. R. China
[8]Center for Molecular Medicine and Genetics, Wayne State University School of Medicine, Detroit, MI, USA
[9]Department of Pediatrics, Wayne State University School of Medicine, Detroit, MI, USA

*Corresponding authors:
Yubin Ge
Department of Oncology
Wayne State University School of Medicine
421 E. Canfield, Detroit, MI 48201
Email: gey@karmanos.org

Jeffrey Taub
Division of Pediatric Hematology and Oncology
Children's Hospital of Michigan
3901 Beaubien St., Detroit, MI 48201
Email: jtaub@med.wayne.edu

Yue Wang
Department of Pediatric Hematology
Children's Medical Center
The First Hospital of Jilin University
2699 Qianjin Street, Changchun, Jilin, P.R. China 130021
Email: wang_yue@jlu.edu.cn

Figure 1B

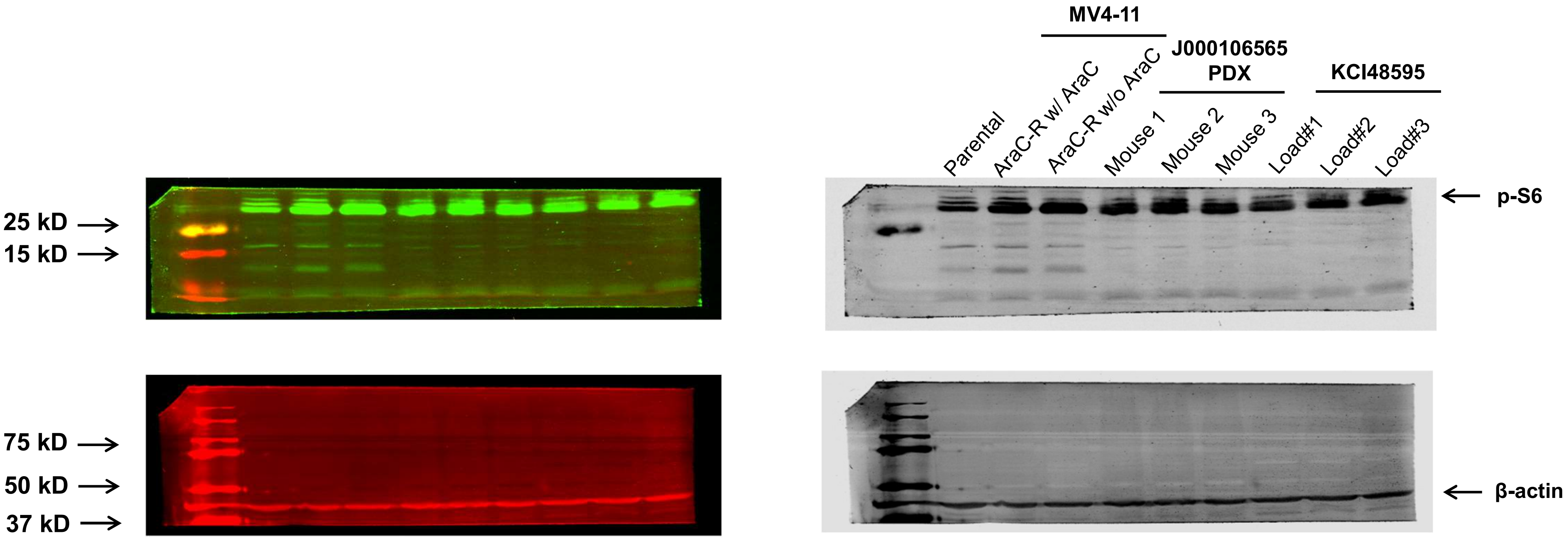

25 kD
15 kD
75 kD
50 kD
37 kD
MV4-11
J000106565
PDX
KCI48595
Parental
AraC-R w/ AraC
AraC-R w/o AraC
Mouse 1
Mouse 2
Mouse 3
Load#1
Load#2
Load#3
p-S6
β-actin

Figure 1B

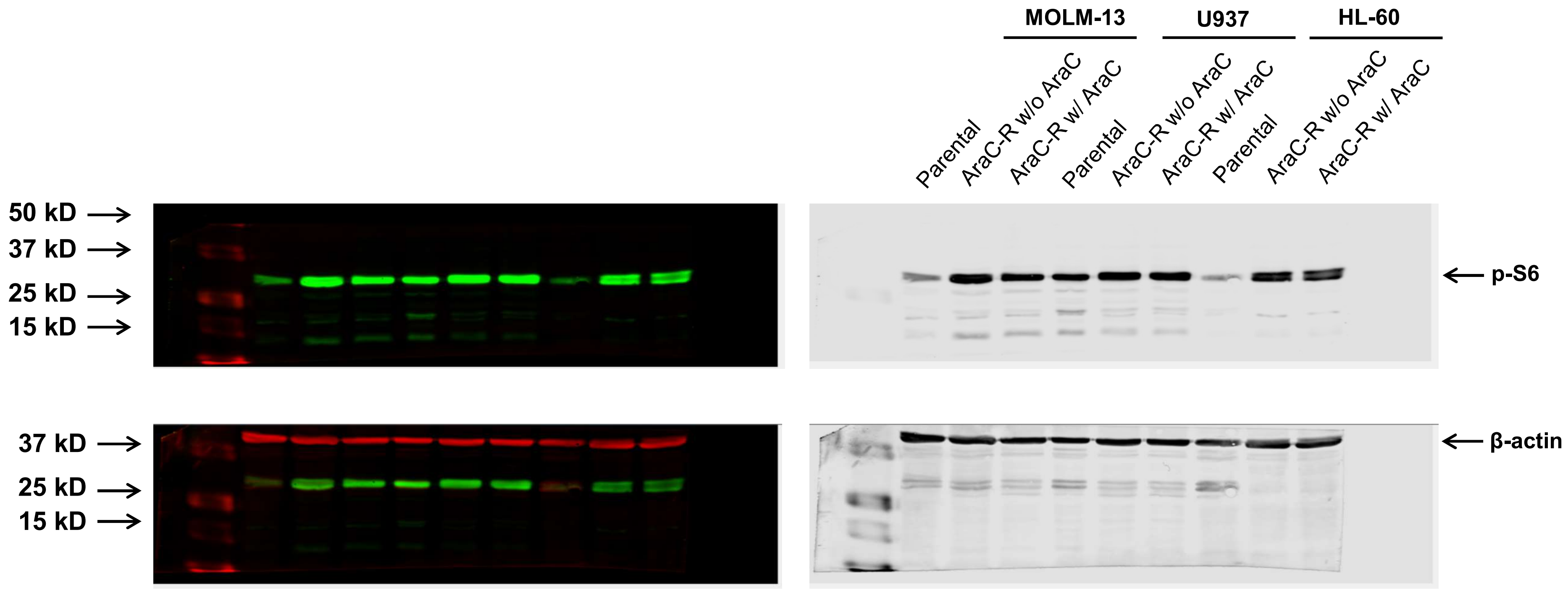

MOLM-13
U937
HL-60
Parental
AraC-R w/o AraC
AraC-R w/ AraC
Parental
AraC-R w/o AraC
AraC-R w/ AraC
Parental
AraC-R w/o AraC
AraC-R w/ AraC
50 kD
37 kD
25 kD
15 kD
p-S6
37 kD
25 kD
15 kD
β-actin

Figure 1C

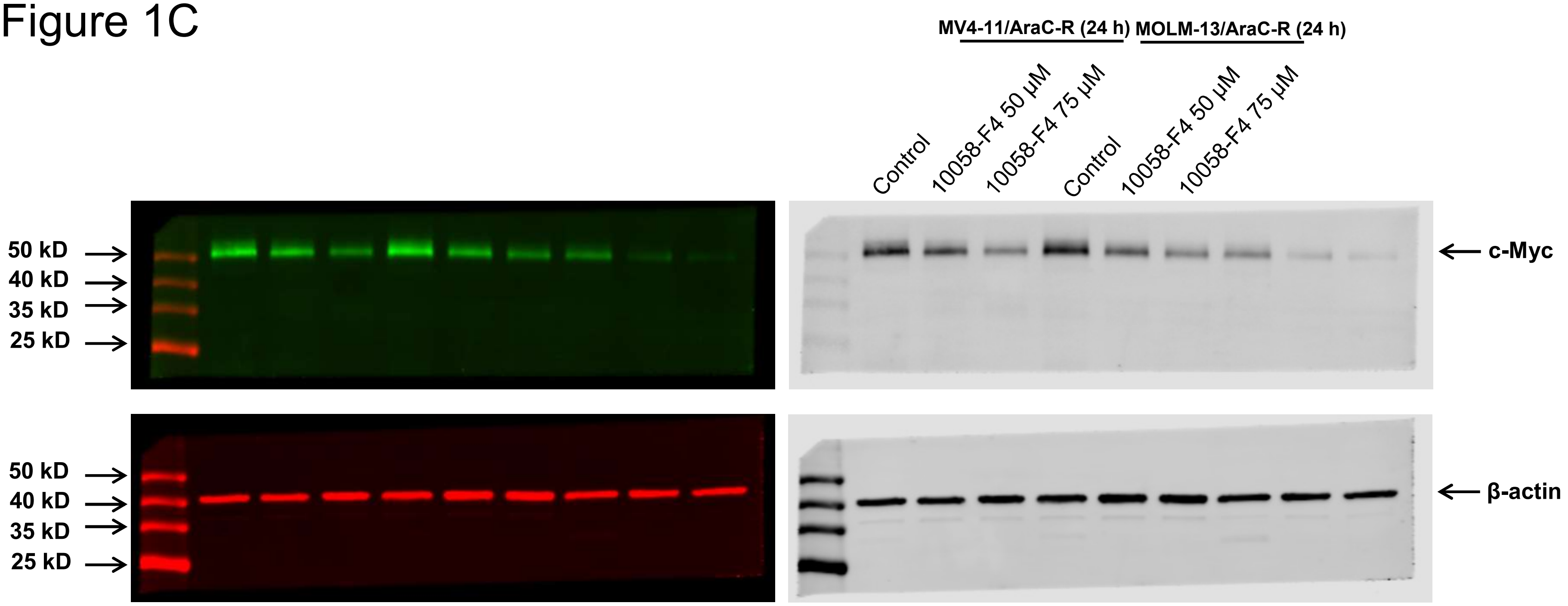

MV4-11/AraC-R (24 h)
MOLM-13/AraC-R (24 h)
Control
10058-F4 50 μM
10058-F4 75 μM
Control
10058-F4 50 μM
10058-F4 75 μM
50 kD
40 kD
35 kD
25 kD
c-Myc
50 kD
40 kD
35 kD
25 kD
β-actin

Figure 1C

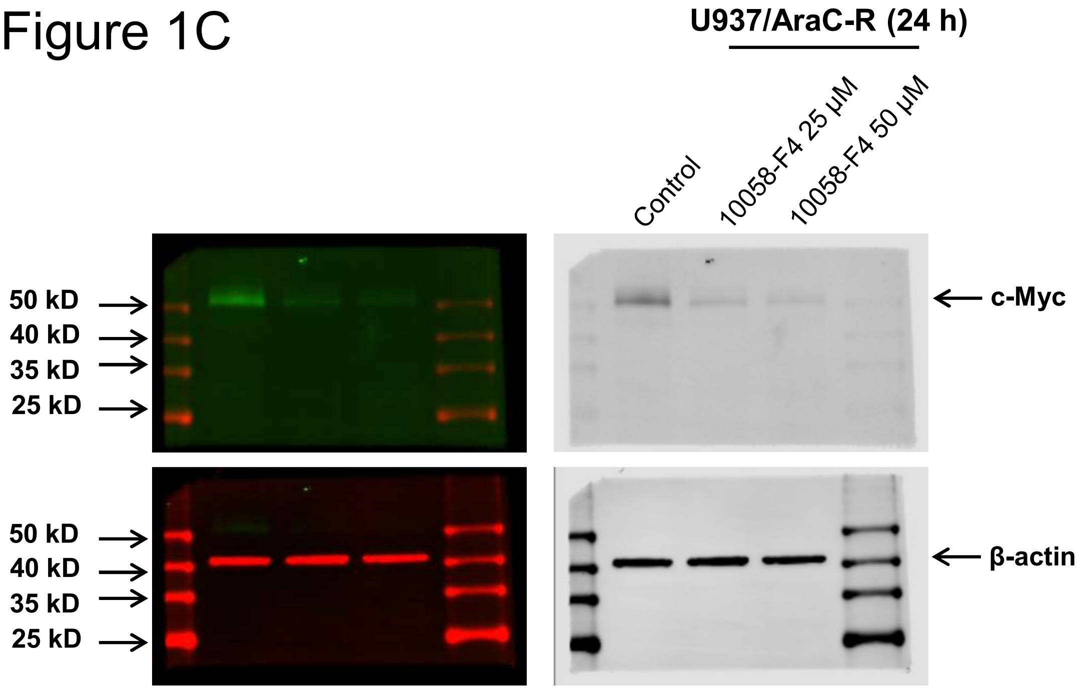

U937/AraC-R (24 h)
Control
10058-F4 25 μM
10058-F4 50 μM
50 kD
40 kD
35 kD
25 kD
c-Myc
50 kD
40 kD
35 kD
25 kD
β-actin

Figure 1E

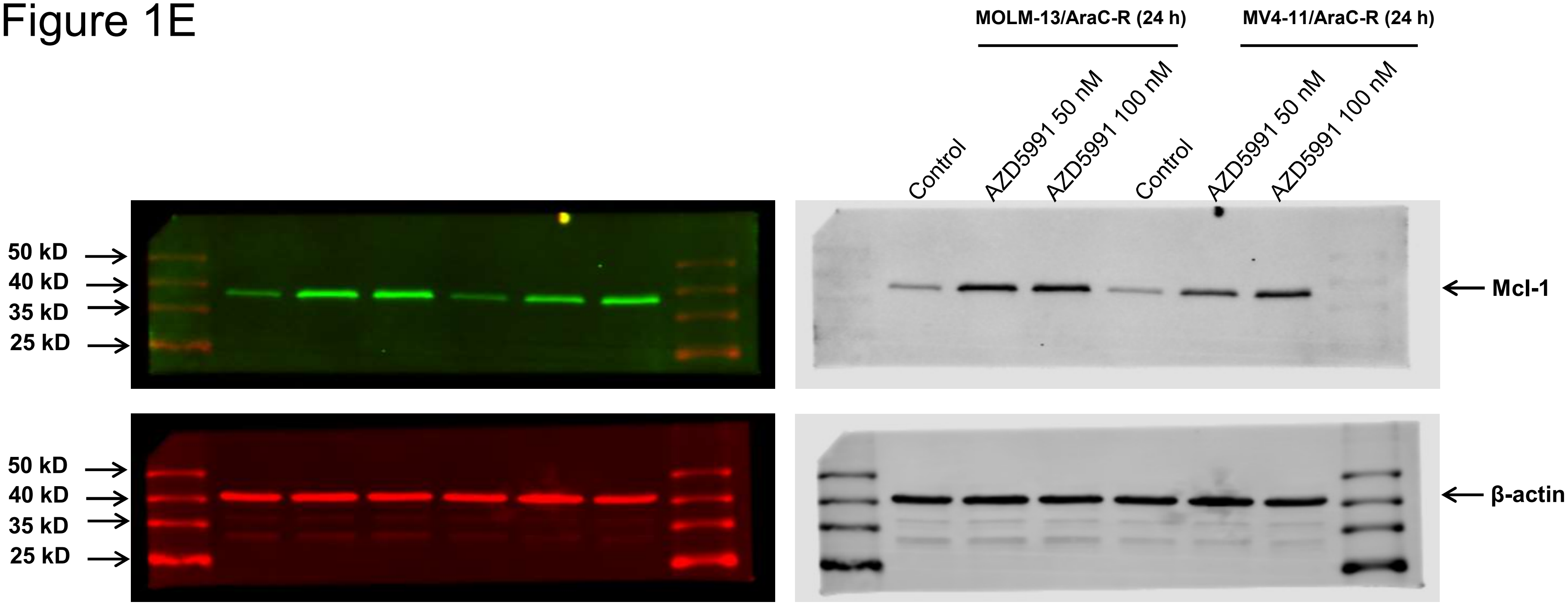

MOLM-13/AraC-R (24 h)
MV4-11/AraC-R (24 h)
Control
AZD5991 50 nM
AZD5991 100 nM
Control
AZD5991 50 nM
AZD5991 100 nM
50 kD
40 kD
35 kD
25 kD
Mcl-1
50 kD
40 kD
35 kD
25 kD
β-actin

Figure 1E

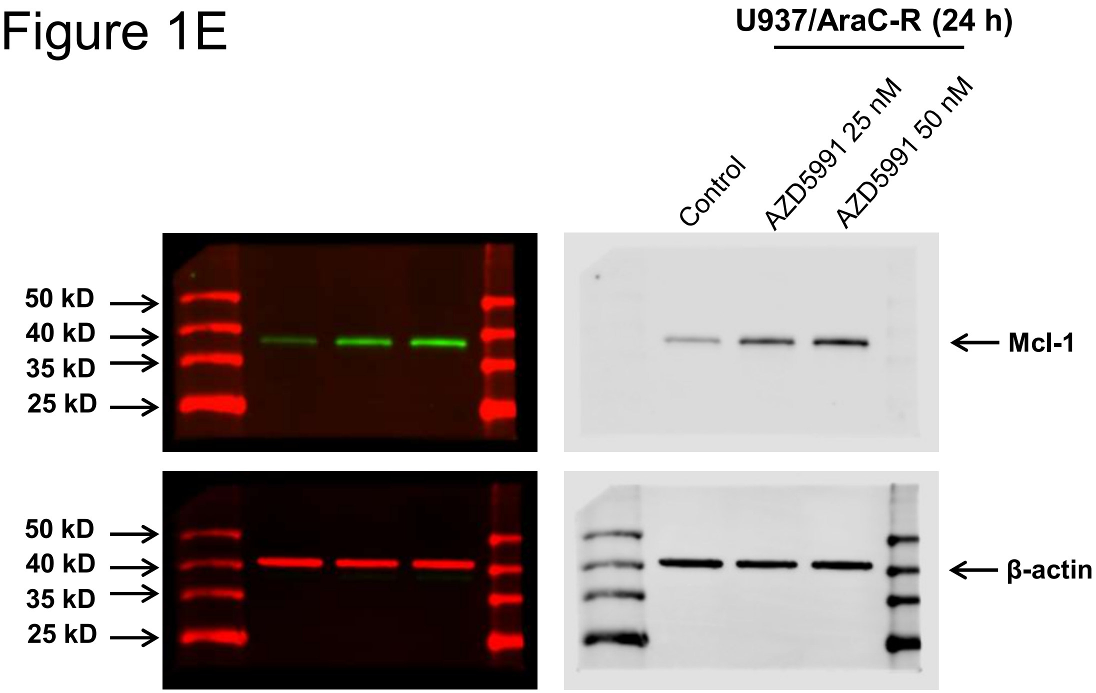
U937/AraC-R (24 h)
Control
AZD5991 25 nM
AZD5991 50 nM
50 kD
40 kD
35 kD
25 kD
Mcl-1
50 kD
40 kD
35 kD
25 kD
β-actin

Figure 1G

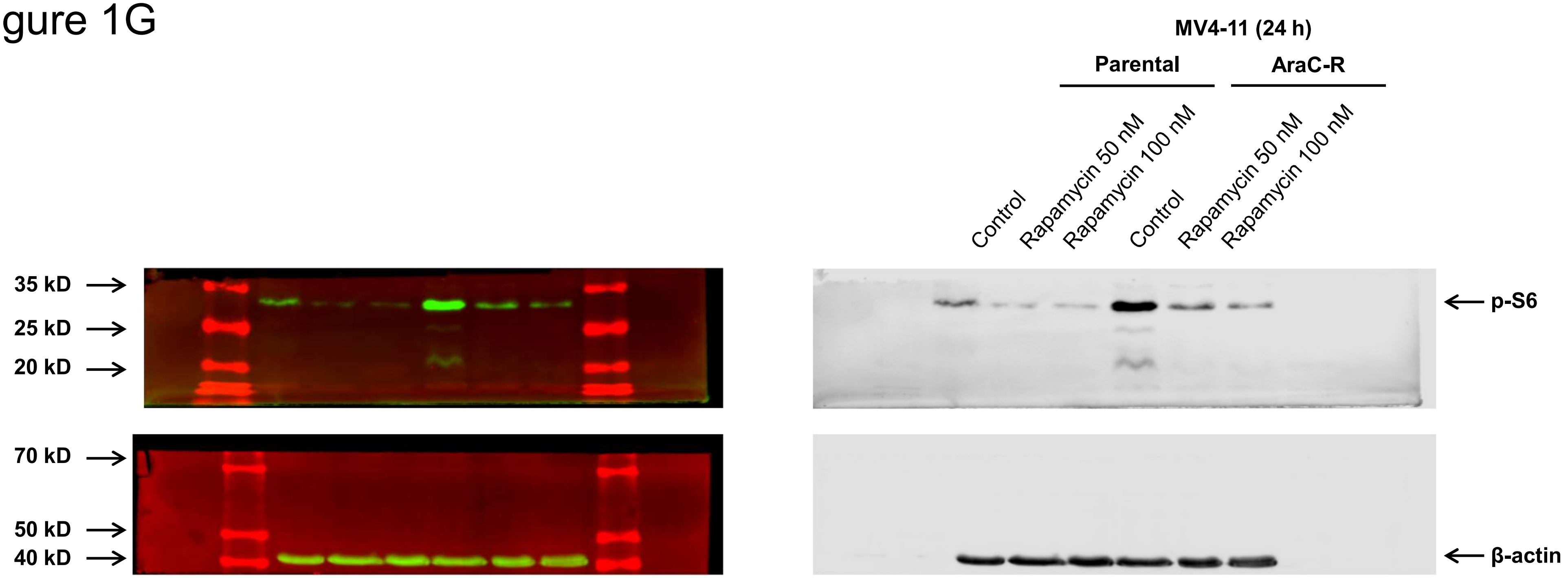

MV4-11 (24 h)
Parental
AraC-R
Control
Rapamycin 50 nM
Rapamycin 100 nM
Control
Rapamycin 50 nM
Rapamycin 100 nM
35 kD
25 kD
20 kD
70 kD
50 kD
40 kD
p-S6
β-actin

Figure 1G

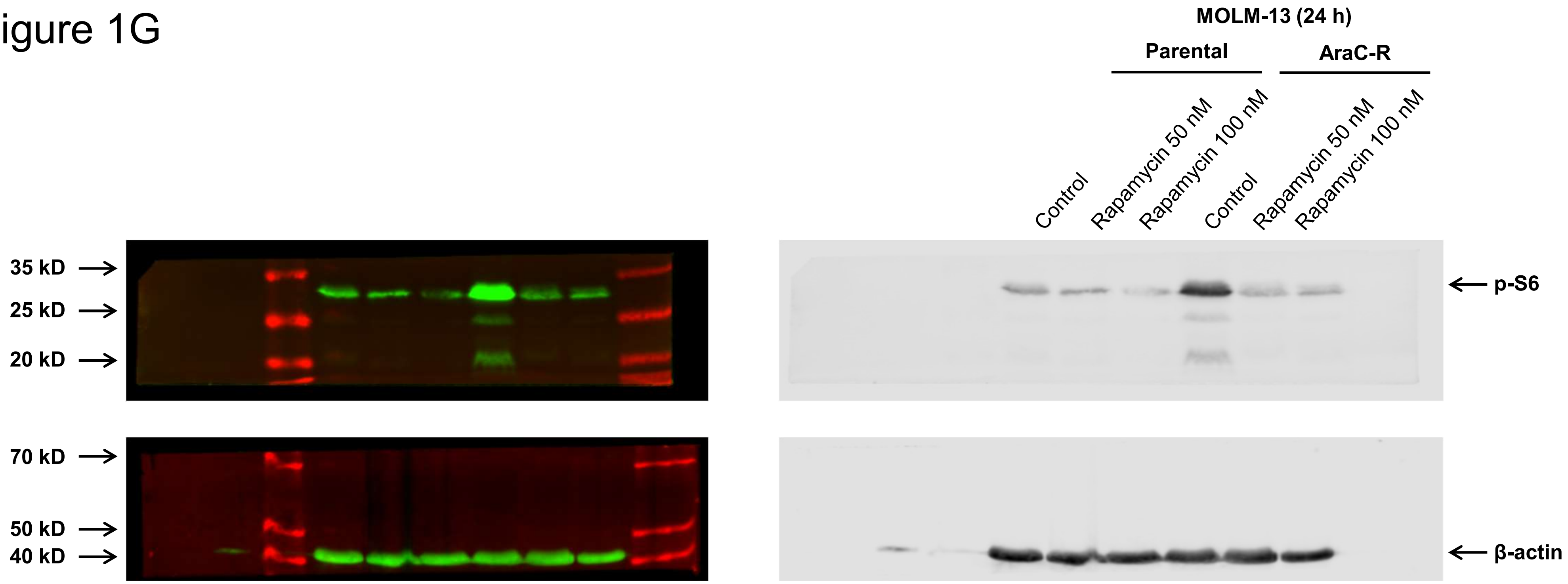
MOLM-13 (24 h)
Parental
AraC-R
Control
Rapamycin 50 nM
Rapamycin 100 nM
Control
Rapamycin 50 nM
Rapamycin 100 nM
35 kD
25 kD
20 kD
70 kD
50 kD
40 kD
p-S6
β-actin

Figure 1G

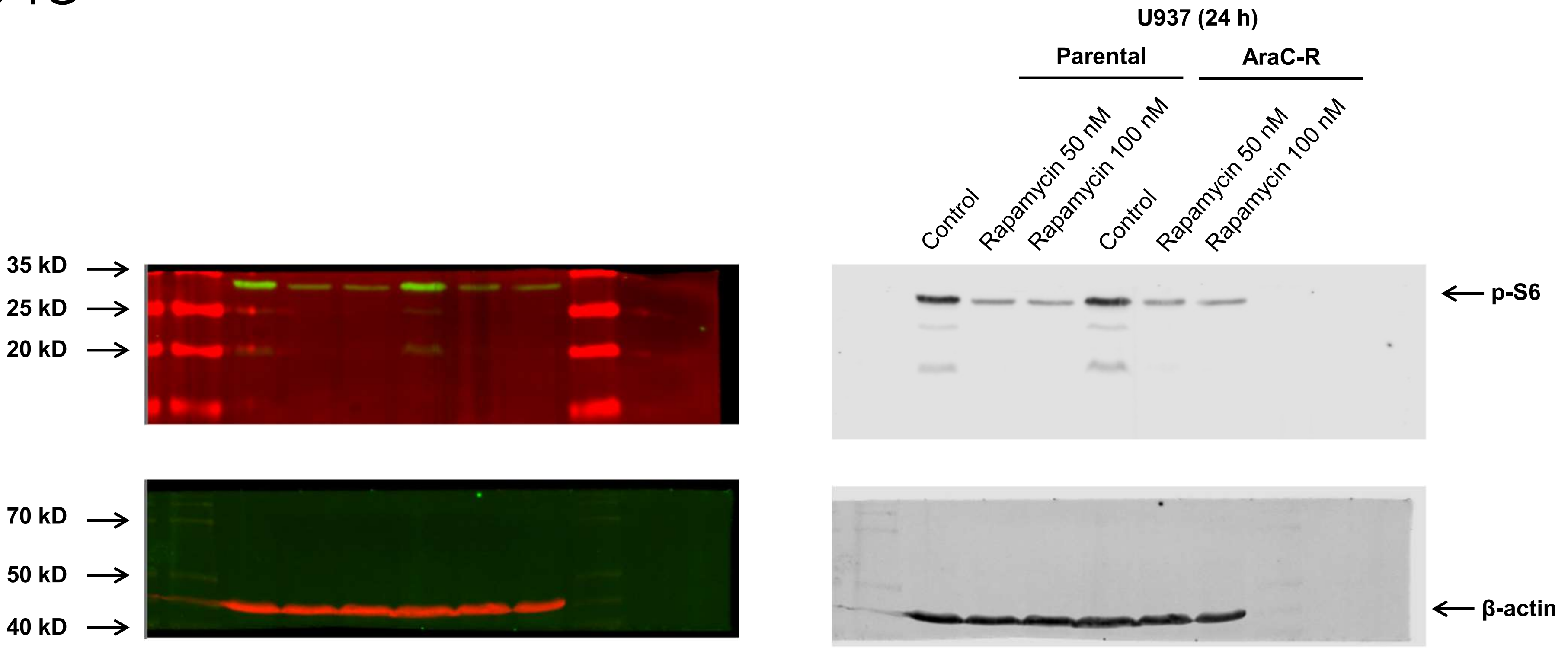
U937 (24 h)
Parental
AraC-R
Control
Rapamycin 50 nM
Rapamycin 100 nM
Control
Rapamycin 50 nM
Rapamycin 100 nM
35 kD
25 kD
20 kD
70 kD
50 kD
40 kD
p-S6
β-actin

Figure 2A

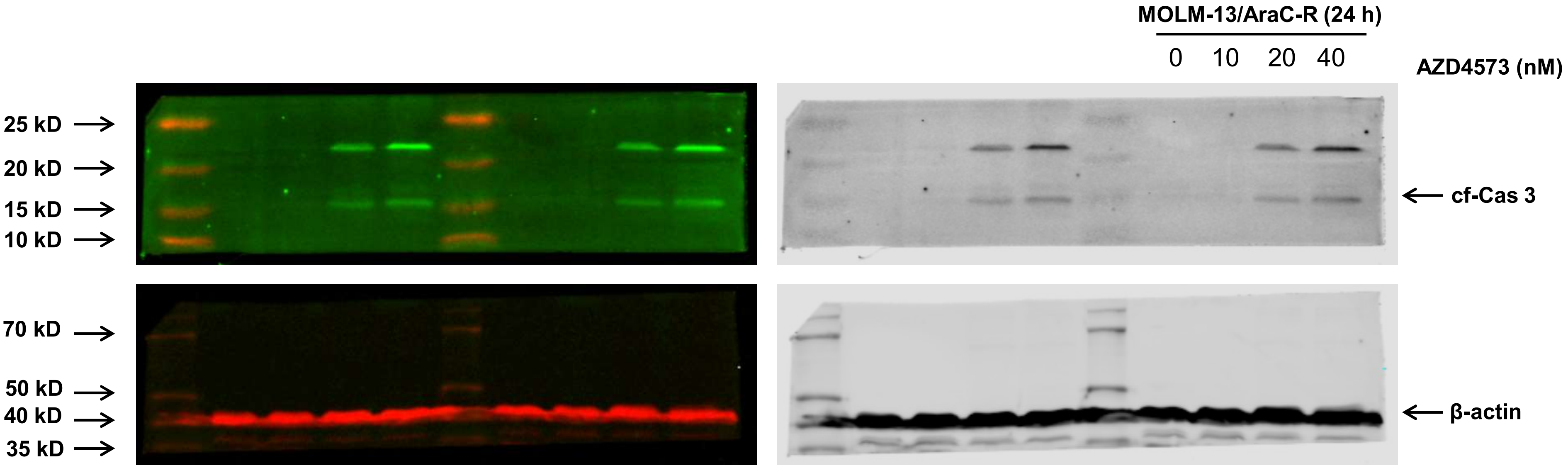

MOLM-13/AraC-R (24 h)
0
10
20
40
AZD4573 (nM)
25 kD
20 kD
15 kD
10 kD
cf-Cas 3
70 kD
50 kD
40 kD
35 kD
β-actin

Figure 2A

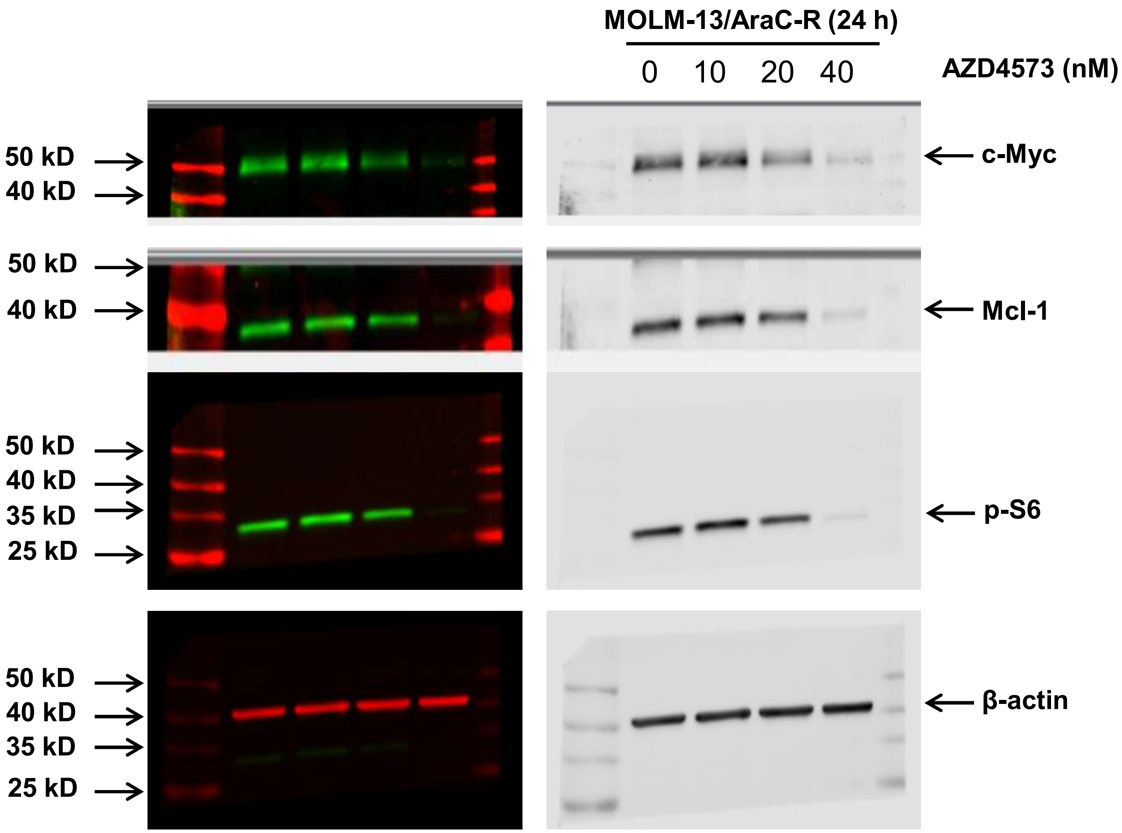
MOLM-13/AraC-R (24 h)
0 10 20 40
AZD4573 (nM)
50 kD
40 kD
c-Myc
50 kD
40 kD
Mcl-1
50 kD
40 kD
35 kD
25 kD
p-S6
50 kD
40 kD
35 kD
25 kD
β-actin

Figure 2A

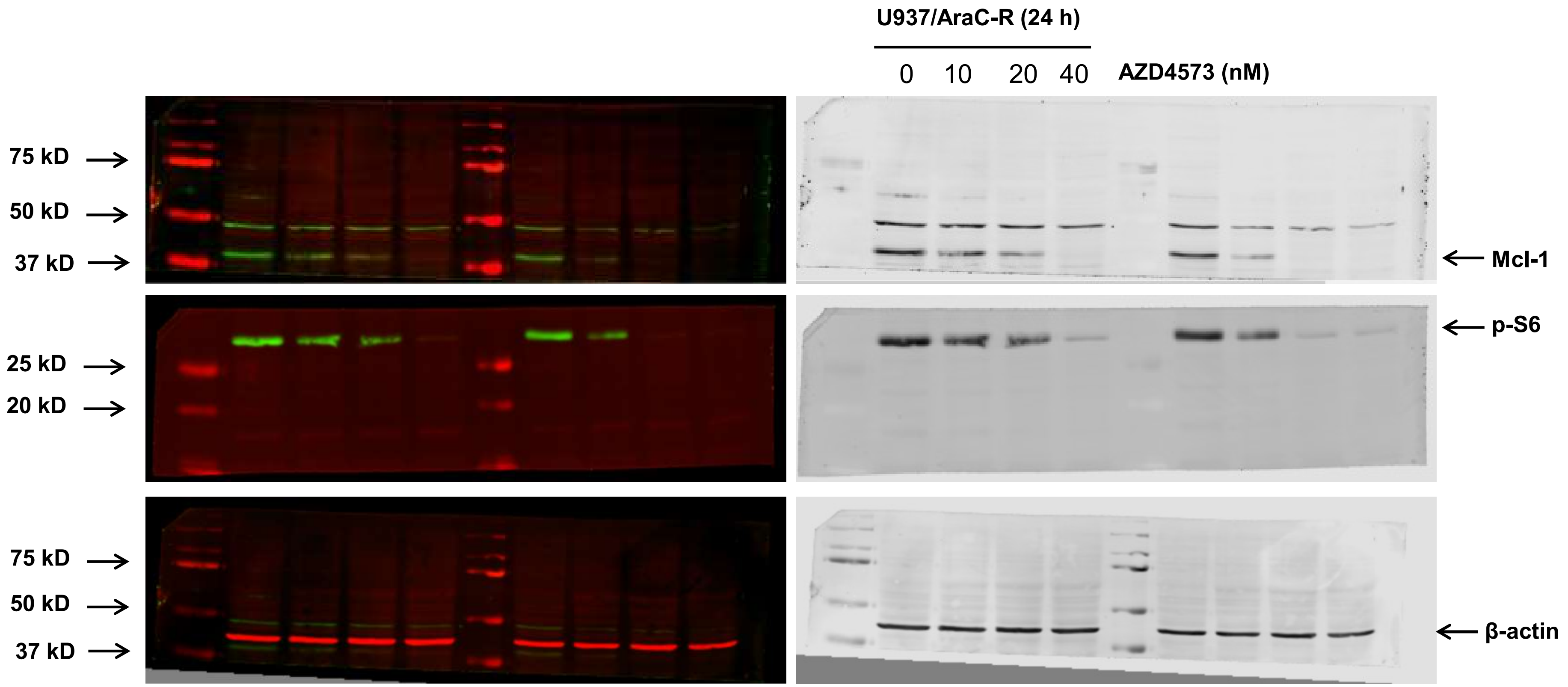

U937/AraC-R (24 h)
0 10 20 40 AZD4573 (nM)
75 kD
50 kD
37 kD
25 kD
20 kD
75 kD
50 kD
37 kD
Mcl-1
p-S6
β-actin

Figure 2A

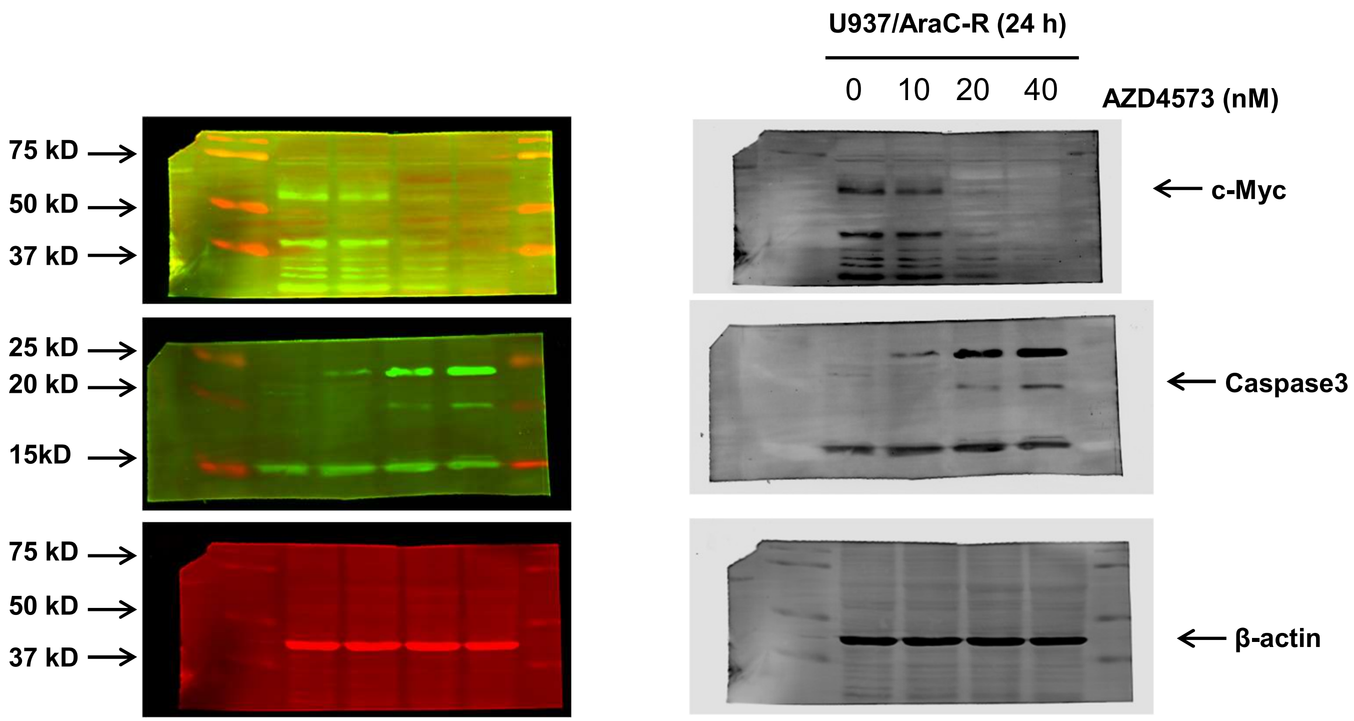

U937/AraC-R (24 h)
0 10 20 40 AZD4573 (nM)
75 kD
50 kD
37 kD
25 kD
20 kD
15kD
75 kD
50 kD
37 kD
c-Myc
Caspase3
β-actin

Figure 2A

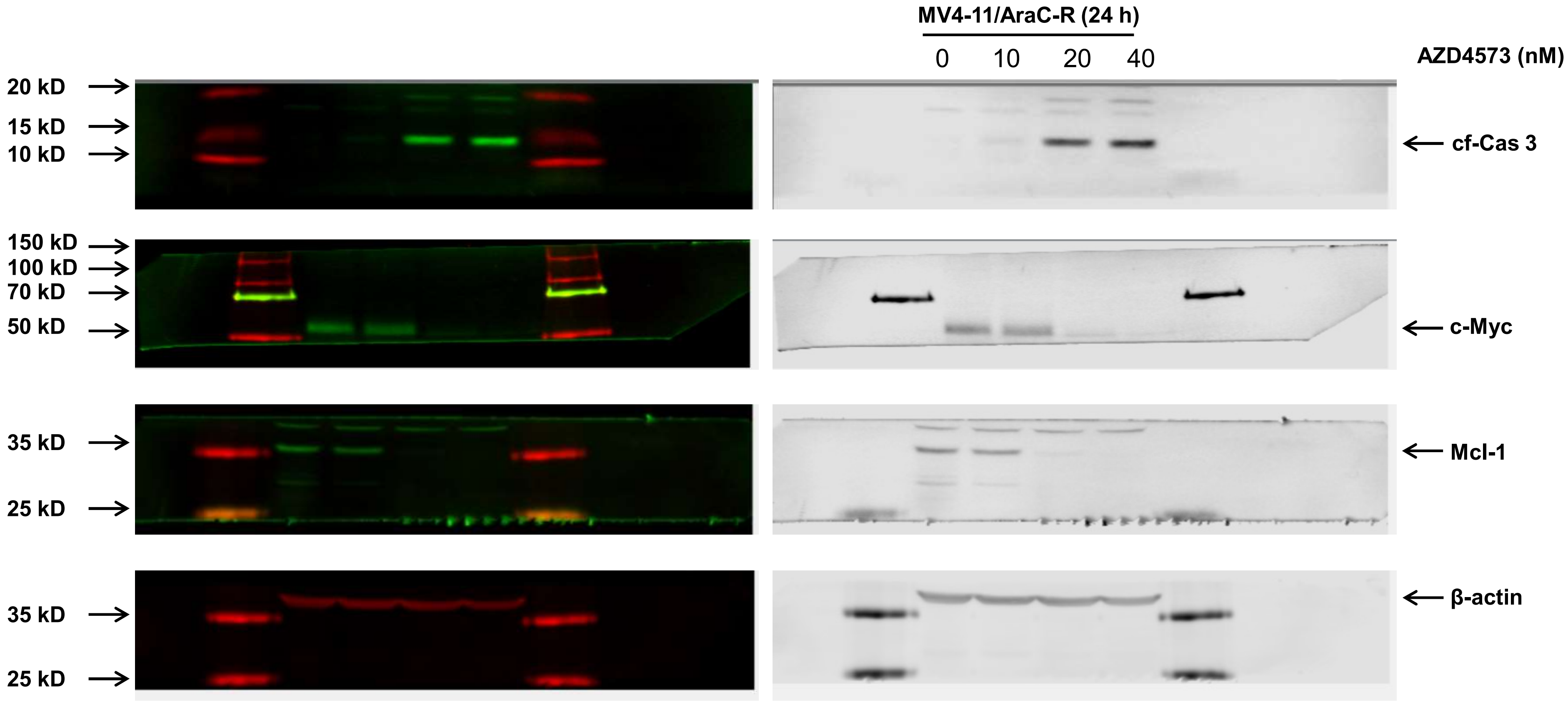
MV4-11/AraC-R (24 h)
0 10 20 40
AZD4573 (nM)
20 kD
15 kD
10 kD
cf-Cas 3
150 kD
100 kD
70 kD
50 kD
c-Myc
35 kD
25 kD
Mcl-1
35 kD
25 kD
β-actin

Figure 2A and 2C

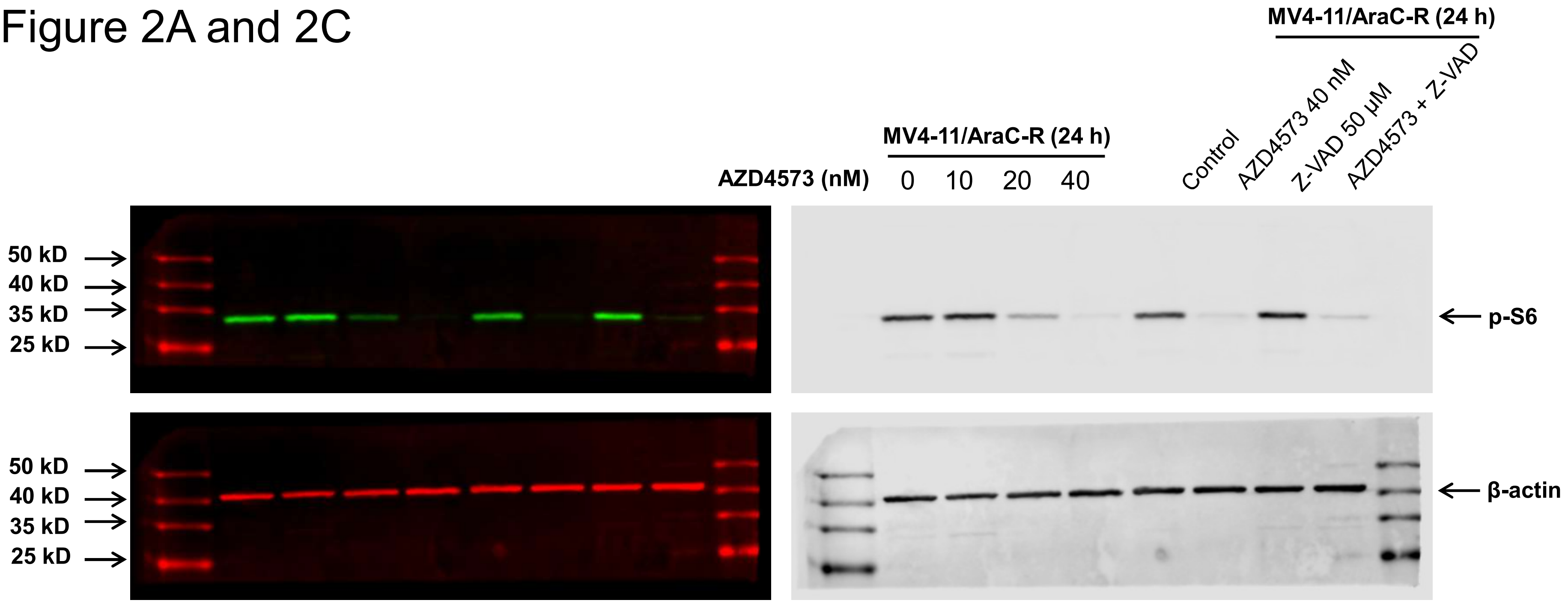

Figure 2B

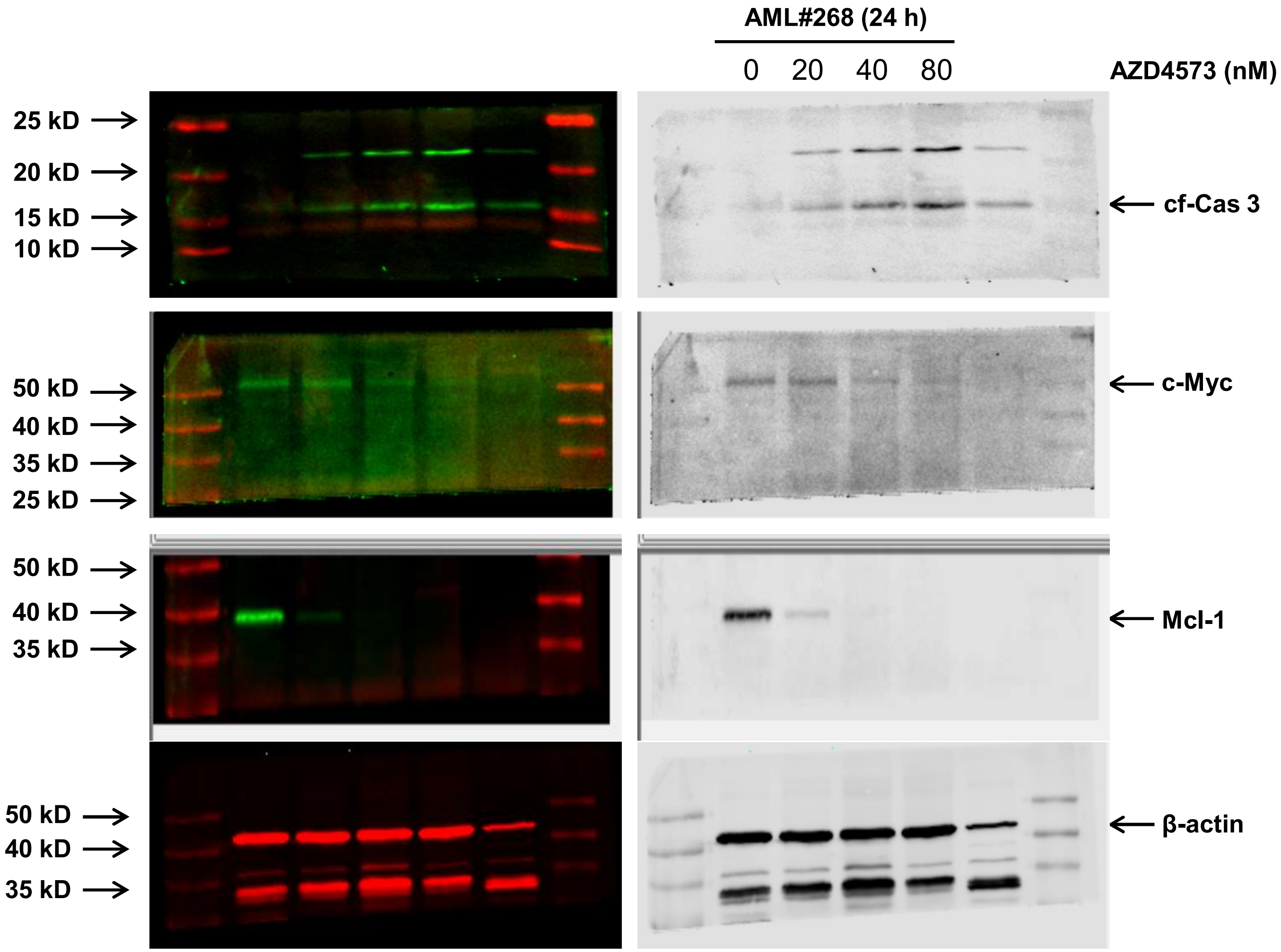
AML#268 (24 h)
0
20
40
80
AZD4573 (nM)
25 kD
20 kD
15 kD
10 kD
cf-Cas 3
50 kD
40 kD
35 kD
25 kD
c-Myc
50 kD
40 kD
35 kD
Mcl-1
50 kD
40 kD
35 kD
β-actin

Figure 2B

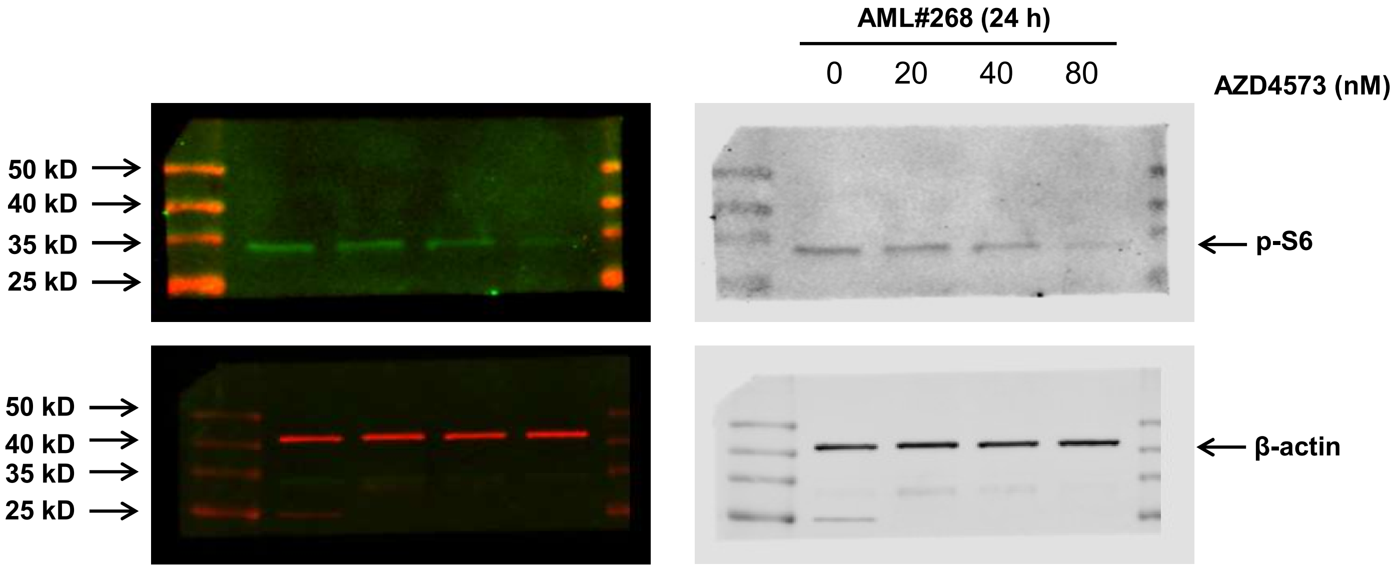

AML#268 (24 h)
0
20
40
80
AZD4573 (nM)
50 kD
40 kD
35 kD
25 kD
p-S6
50 kD
40 kD
35 kD
25 kD
β-actin

Figure 2B

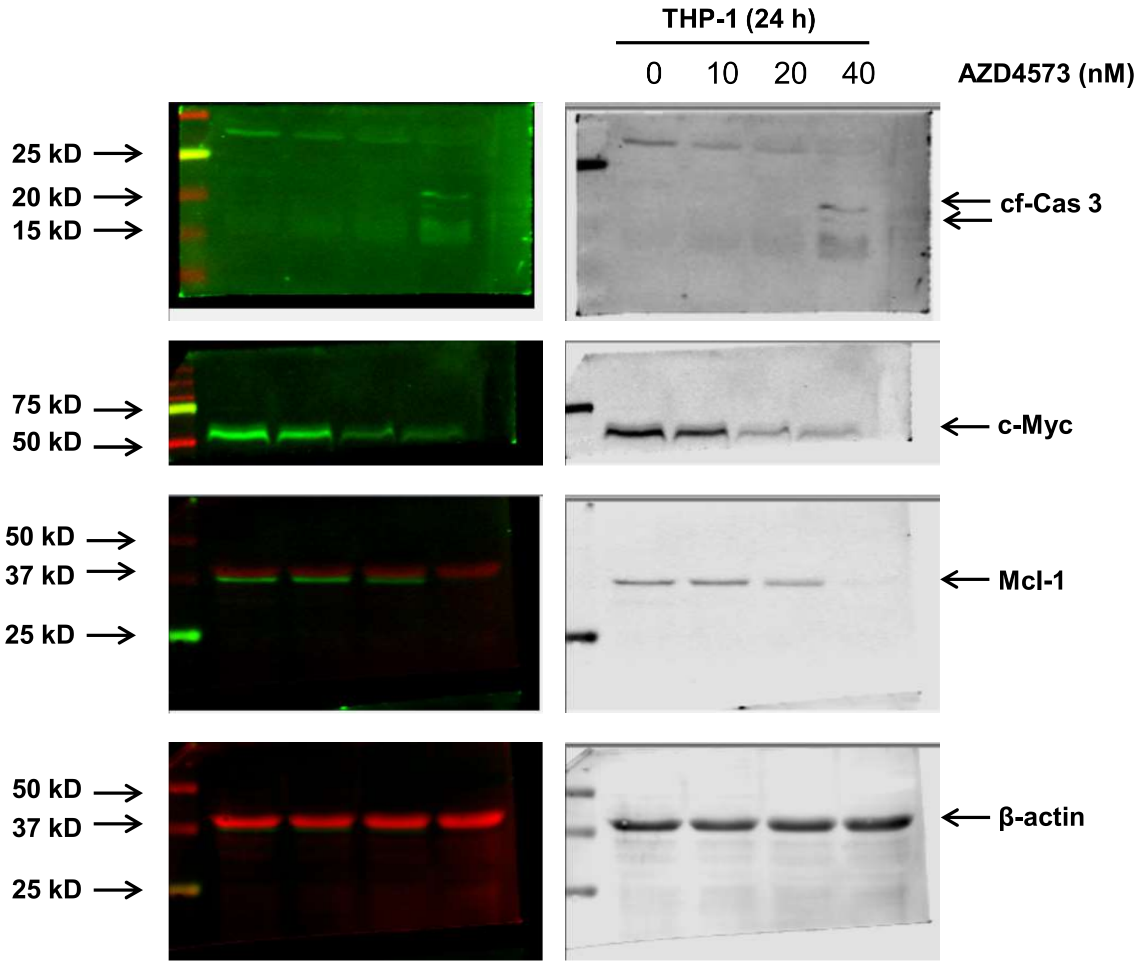

THP-1 (24 h)
0
10
20
40
AZD4573 (nM)
25 kD
20 kD
15 kD
cf-Cas 3
75 kD
50 kD
c-Myc
50 kD
37 kD
25 kD
Mcl-1
50 kD
37 kD
25 kD
β-actin

Figure 2B and 2C

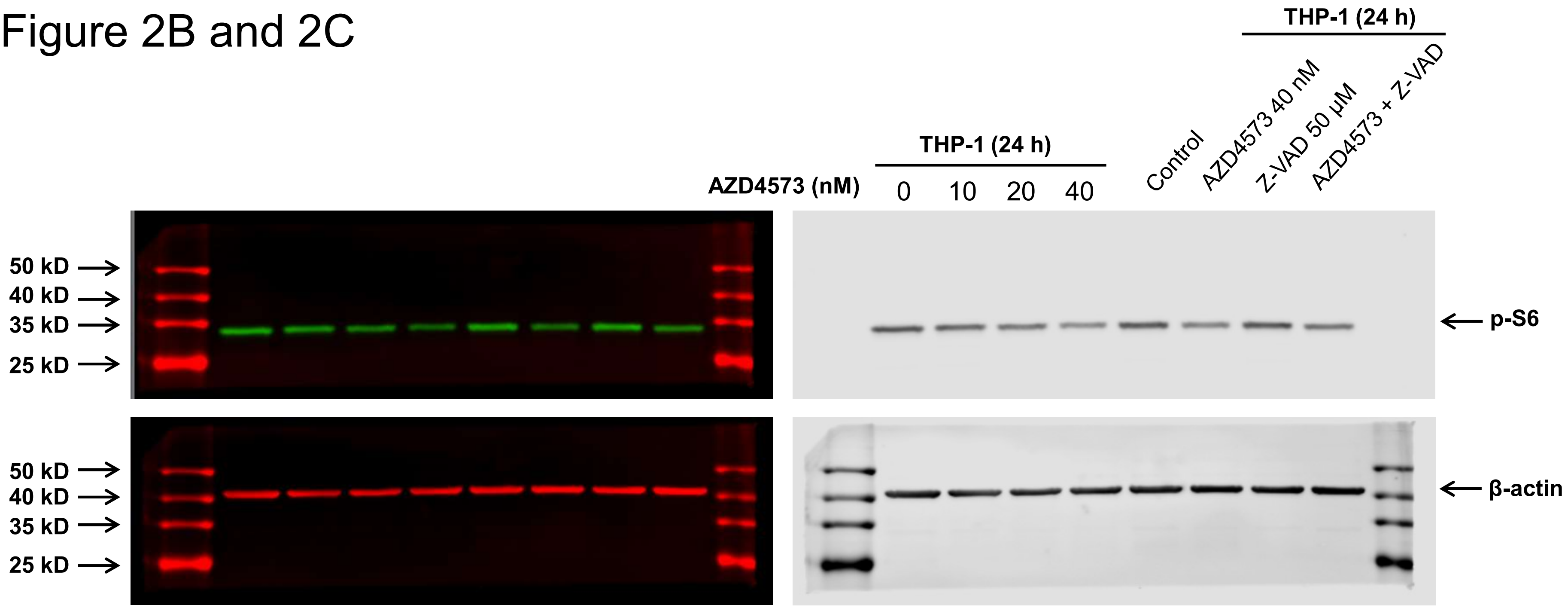

Figure 2C

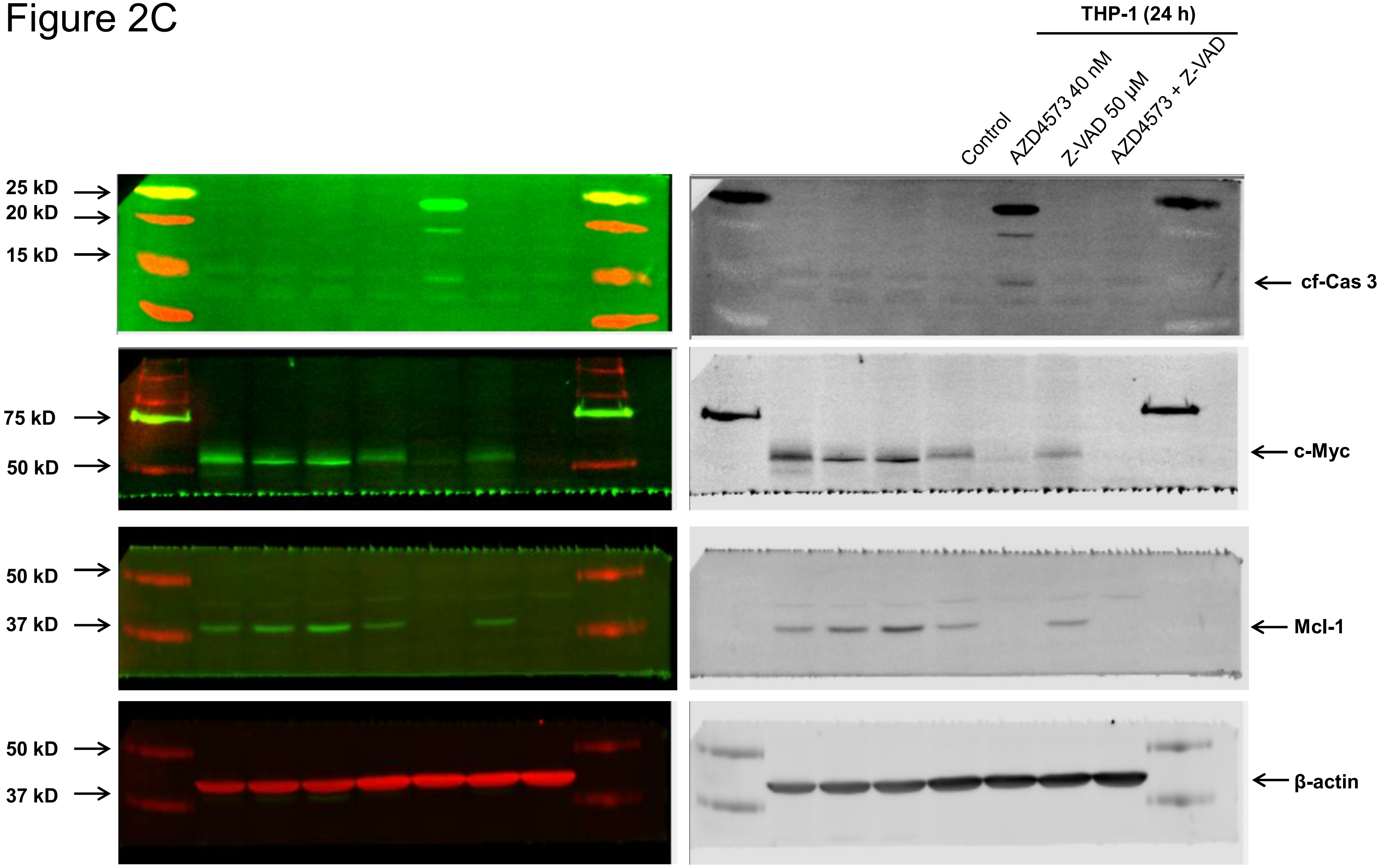
THP-1 (24 h)
Control
AZD4573 40 nM
Z-VAD 50 µM
AZD4573 + Z-VAD
25 kD
20 kD
15 kD
cf-Cas 3
75 kD
50 kD
c-Myc
50 kD
37 kD
Mcl-1
50 kD
37 kD
β-actin

Figure 2C

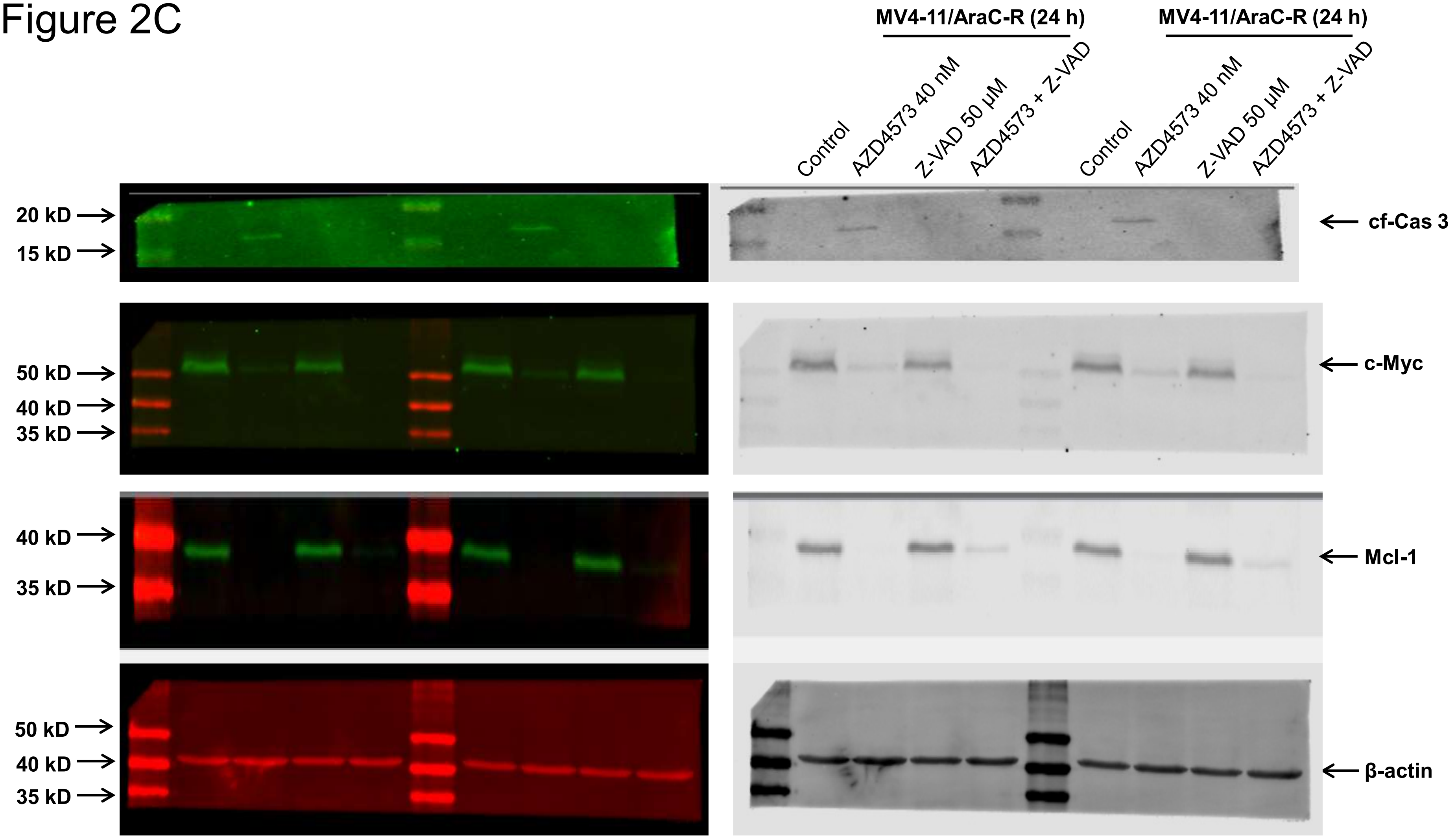

MV4-11/AraC-R (24 h)
MV4-11/AraC-R (24 h)
Control
AZD4573 40 nM
Z-VAD 50 μM
AZD4573 + Z-VAD
Control
AZD4573 40 nM
Z-VAD 50 μM
AZD4573 + Z-VAD
20 kD
15 kD
cf-Cas 3
50 kD
40 kD
35 kD
c-Myc
40 kD
35 kD
Mcl-1
50 kD
40 kD
35 kD
β-actin

Figure 2G

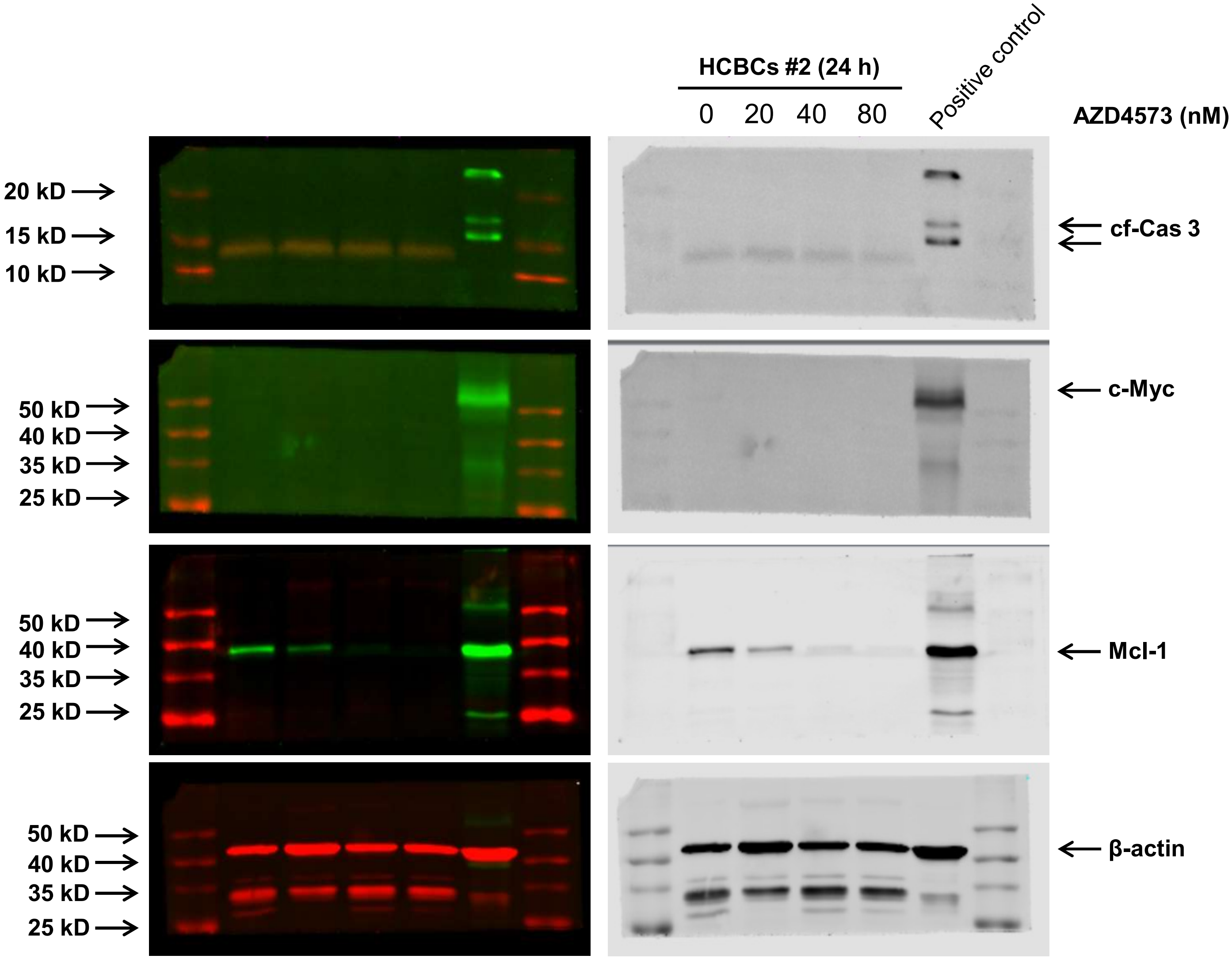

HCBCs #2 (24 h)
0
20
40
80
Positive control
AZD4573 (nM)
20 kD
15 kD
10 kD
cf-Cas 3
50 kD
40 kD
35 kD
25 kD
c-Myc
50 kD
40 kD
35 kD
25 kD
Mcl-1
50 kD
40 kD
35 kD
25 kD
β-actin

Figure 2G

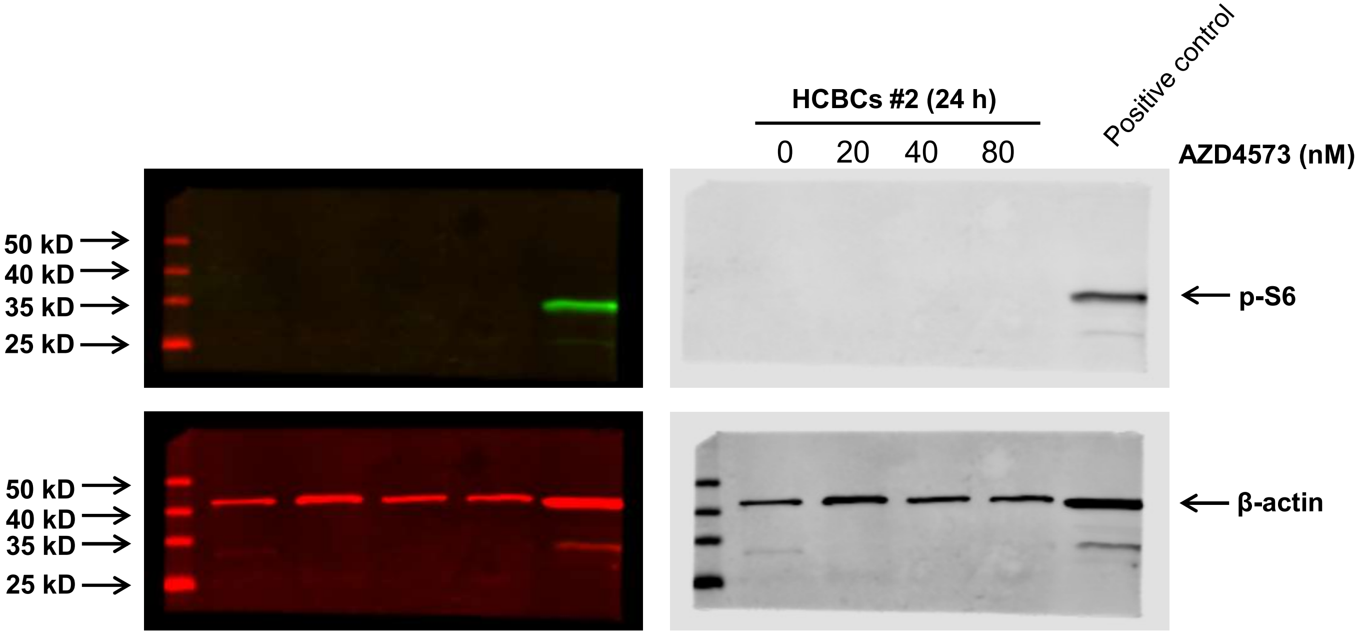

HCBCs #2 (24 h)
Positive control
0
20
40
80
AZD4573 (nM)
50 kD
40 kD
35 kD
25 kD
p-S6
50 kD
40 kD
35 kD
25 kD
β-actin

Figure 2J

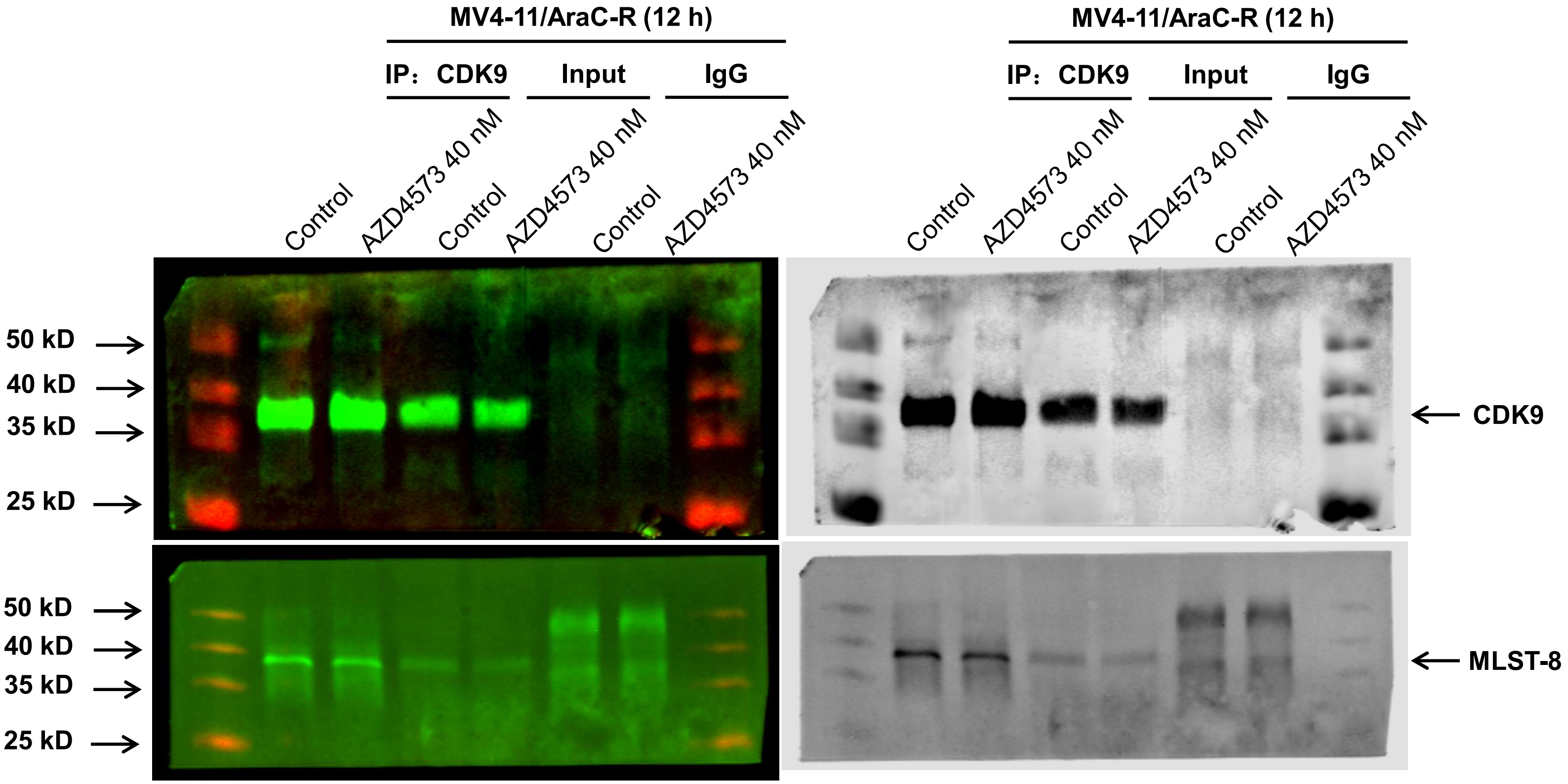
MV4-11/AraC-R (12 h)
IP：CDK9
Input
IgG
Control
AZD4573 40 nM
Control
AZD4573 40 nM
Control
AZD4573 40 nM
50 kD
40 kD
35 kD
25 kD
50 kD
40 kD
35 kD
25 kD
MV4-11/AraC-R (12 h)
IP：CDK9
Input
IgG
Control
AZD4573 40 nM
Control
AZD4573 40 nM
Control
AZD4573 40 nM
CDK9
MLST-8

Figure 2J

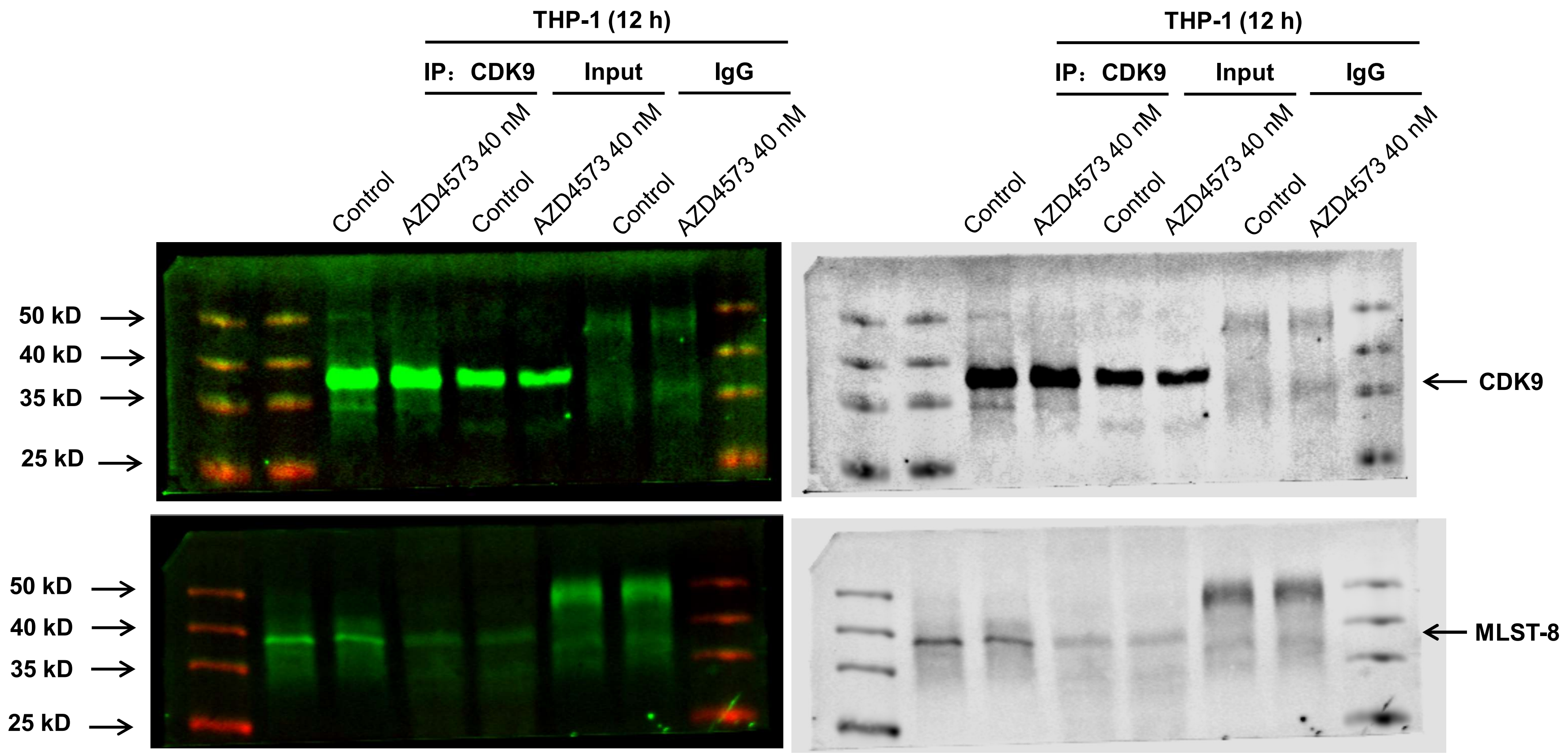

THP-1 (12 h)
IP：CDK9
Input
IgG
Control
AZD4573 40 nM
Control
AZD4573 40 nM
Control
AZD4573 40 nM
50 kD
40 kD
35 kD
25 kD
THP-1 (12 h)
IP：CDK9
Input
IgG
Control
AZD4573 40 nM
Control
AZD4573 40 nM
Control
AZD4573 40 nM
CDK9
50 kD
40 kD
35 kD
25 kD
MLST-8

Figure 4D

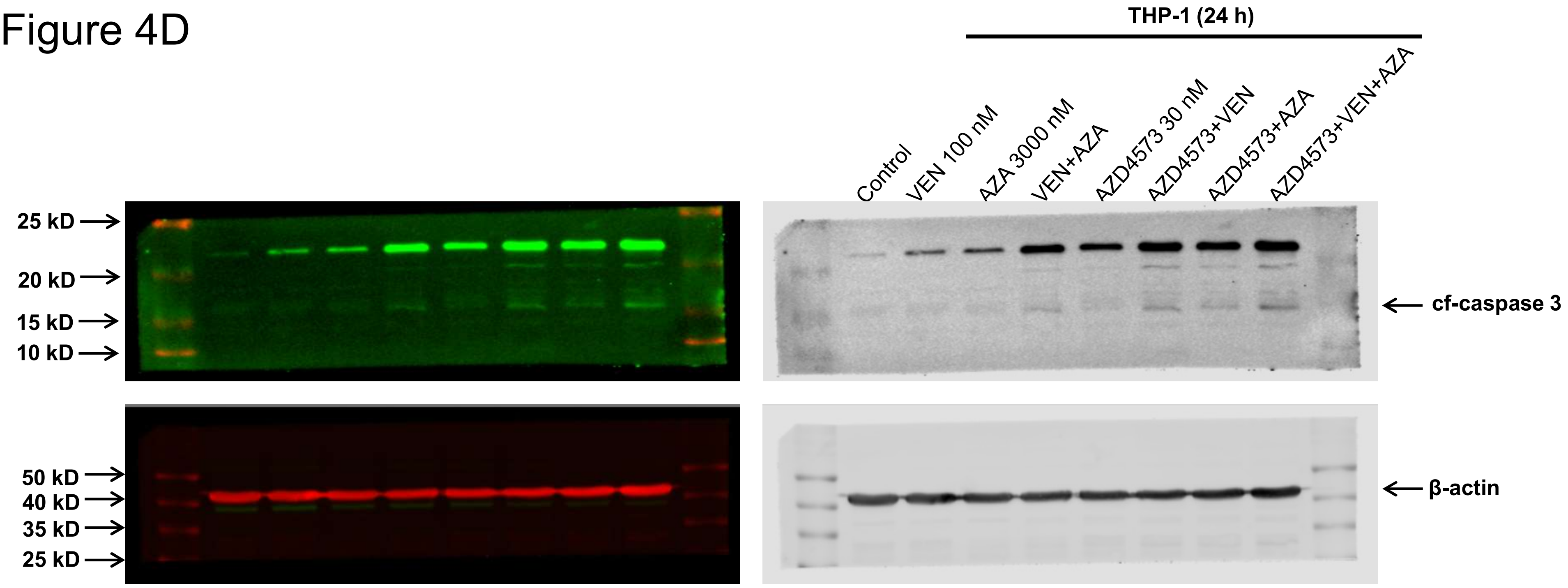

THP-1 (24 h)
Control
VEN 100 nM
AZA 3000 nM
VEN+AZA
AZD4573 30 nM
AZD4573+VEN
AZD4573+AZA
AZD4573+VEN+AZA
25 kD
20 kD
15 kD
10 kD
cf-caspase 3
50 kD
40 kD
35 kD
25 kD
β-actin

Figure 4D

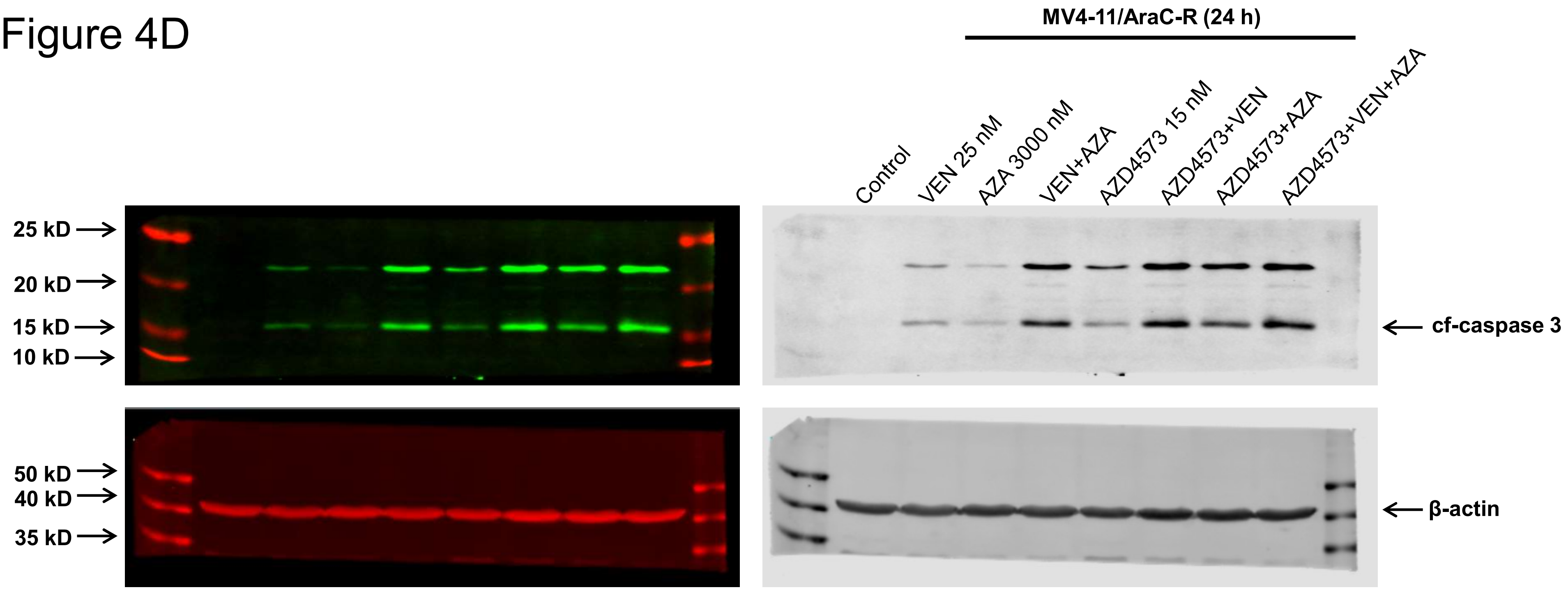

MV4-11/AraC-R (24 h)
Control
VEN 25 nM
AZA 3000 nM
VEN+AZA
AZD4573 15 nM
AZD4573+VEN
AZD4573+AZA
AZD4573+VEN+AZA
25 kD
20 kD
15 kD
10 kD
cf-caspase 3
50 kD
40 kD
35 kD
β-actin

Figure 4D

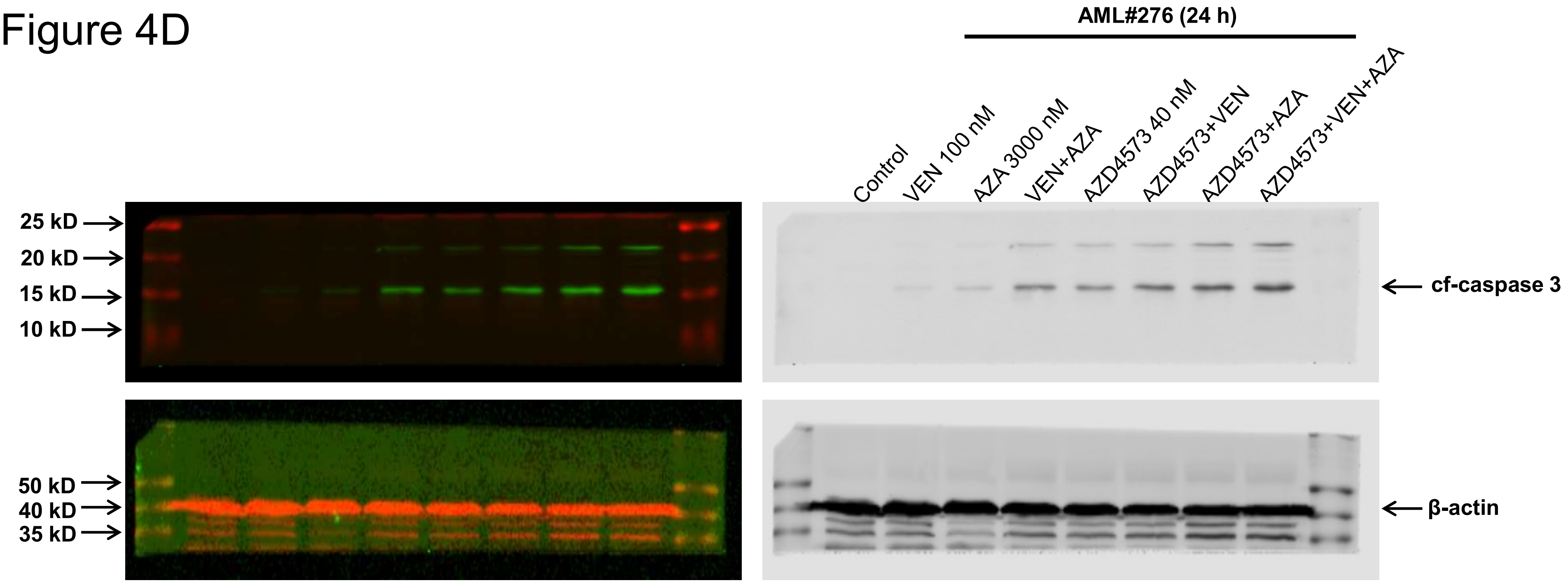
AML#276 (24 h)
Control
VEN 100 nM
AZA 3000 nM
VEN+AZA
AZD4573 40 nM
AZD4573+VEN
AZD4573+AZA
AZD4573+VEN+AZA
25 kD
20 kD
15 kD
10 kD
cf-caspase 3
50 kD
40 kD
35 kD
β-actin

Figure 5A

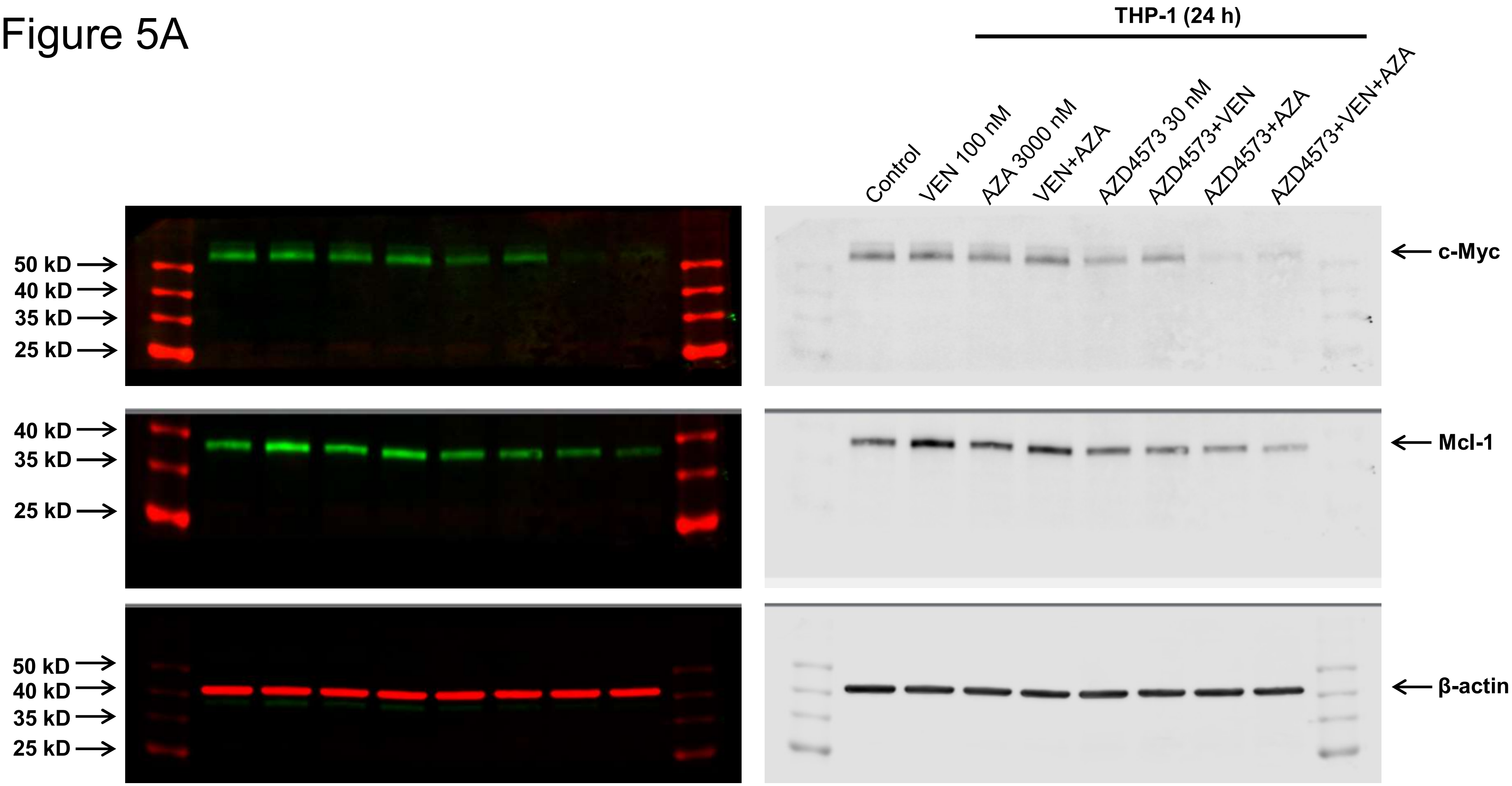

THP-1 (24 h)
Control
VEN 100 nM
AZA 3000 nM
VEN+AZA
AZD4573 30 nM
AZD4573+VEN
AZD4573+AZA
AZD4573+VEN+AZA
50 kD
40 kD
35 kD
25 kD
c-Myc
40 kD
35 kD
25 kD
Mcl-1
50 kD
40 kD
35 kD
25 kD
β-actin

Figure 5A

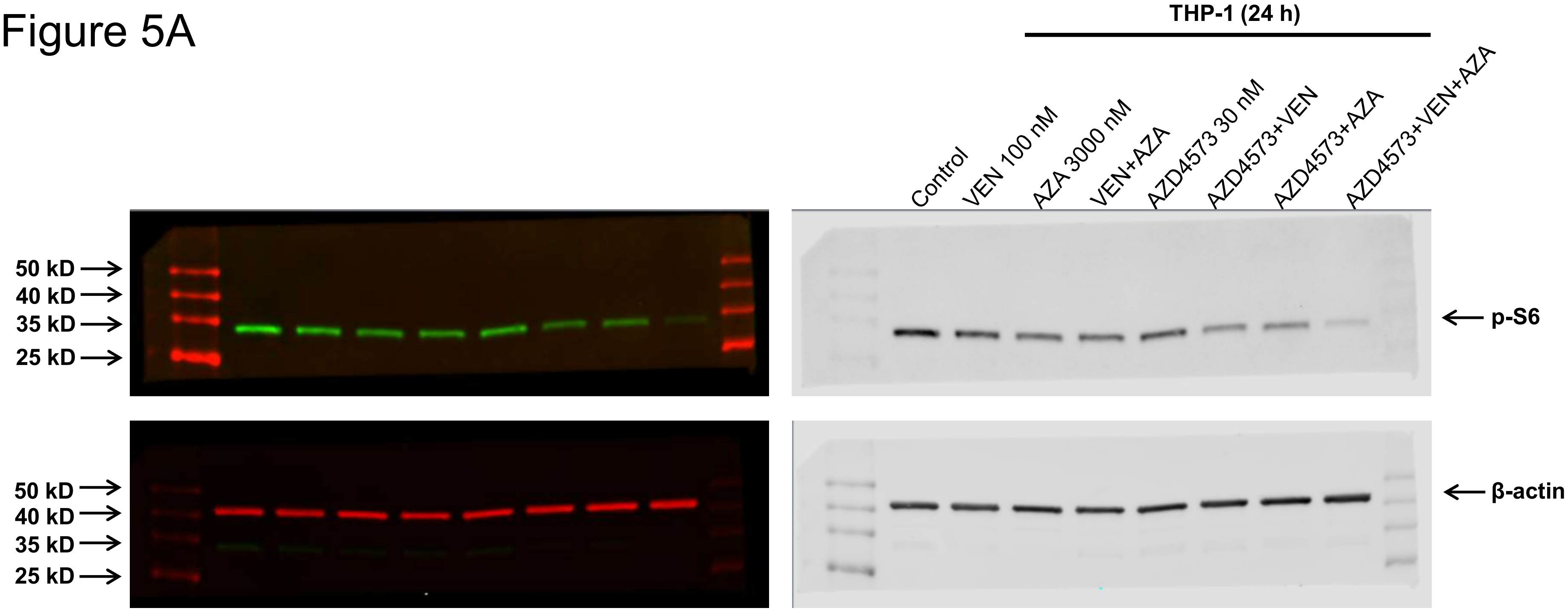
THP-1 (24 h)
Control
VEN 100 nM
AZA 3000 nM
VEN+AZA
AZD4573 30 nM
AZD4573+VEN
AZD4573+AZA
AZD4573+VEN+AZA
50 kD
40 kD
35 kD
25 kD
p-S6
50 kD
40 kD
35 kD
25 kD
β-actin

Figure 5A

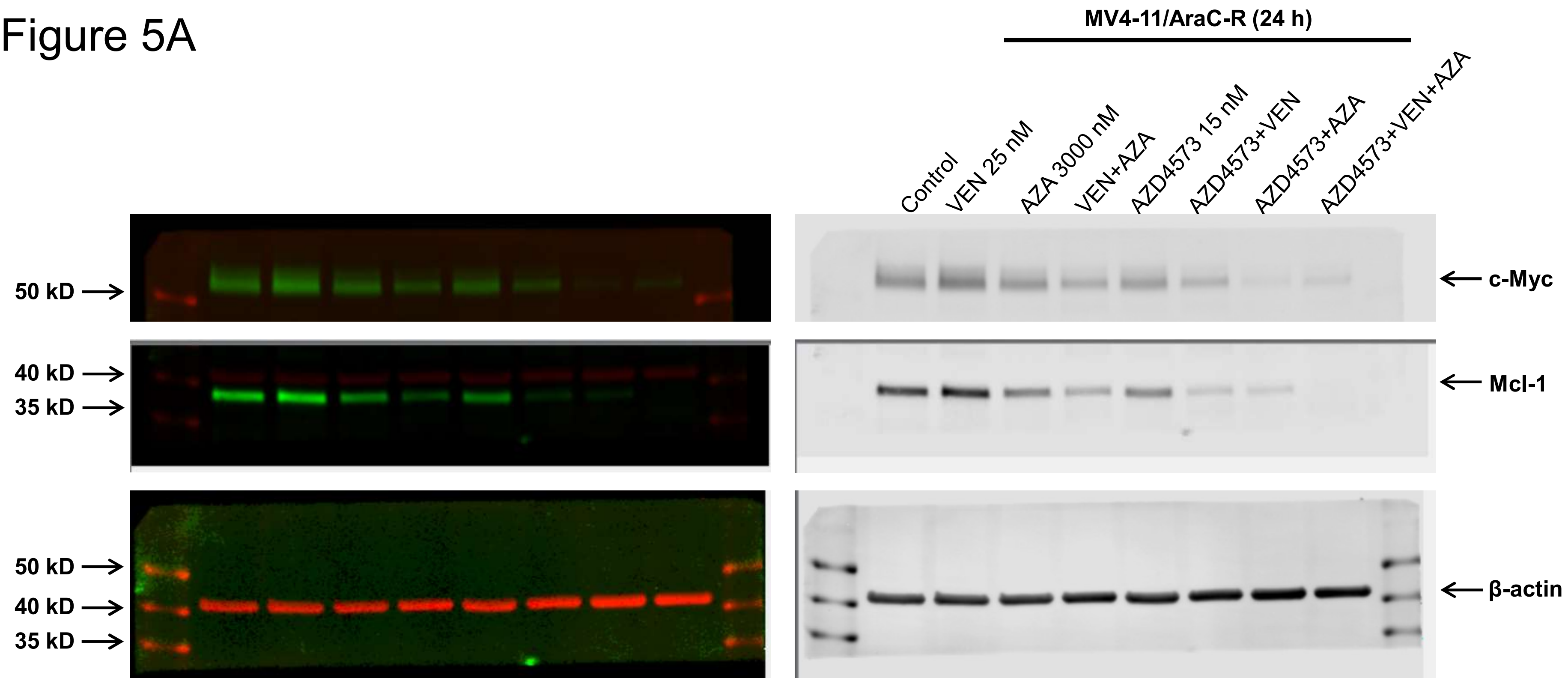

MV4-11/AraC-R (24 h)
Control
VEN 25 nM
AZA 3000 nM
VEN+AZA
AZD4573 15 nM
AZD4573+VEN
AZD4573+AZA
AZD4573+VEN+AZA
50 kD
40 kD
35 kD
50 kD
40 kD
35 kD
c-Myc
Mcl-1
β-actin

Figure 5A

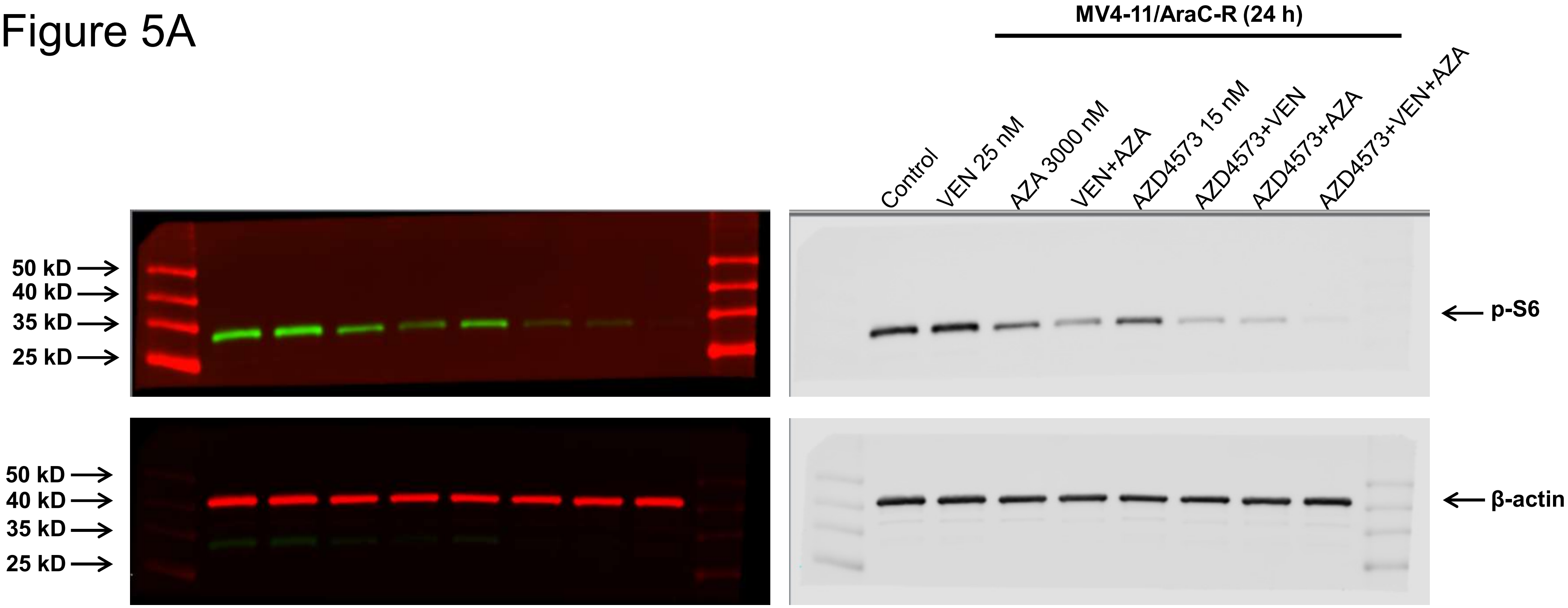

MV4-11/AraC-R (24 h)
Control
VEN 25 nM
AZA 3000 nM
VEN+AZA
AZD4573 15 nM
AZD4573+VEN
AZD4573+AZA
AZD4573+VEN+AZA
50 kD
40 kD
35 kD
25 kD
p-S6
50 kD
40 kD
35 kD
25 kD
β-actin

Figure 5A

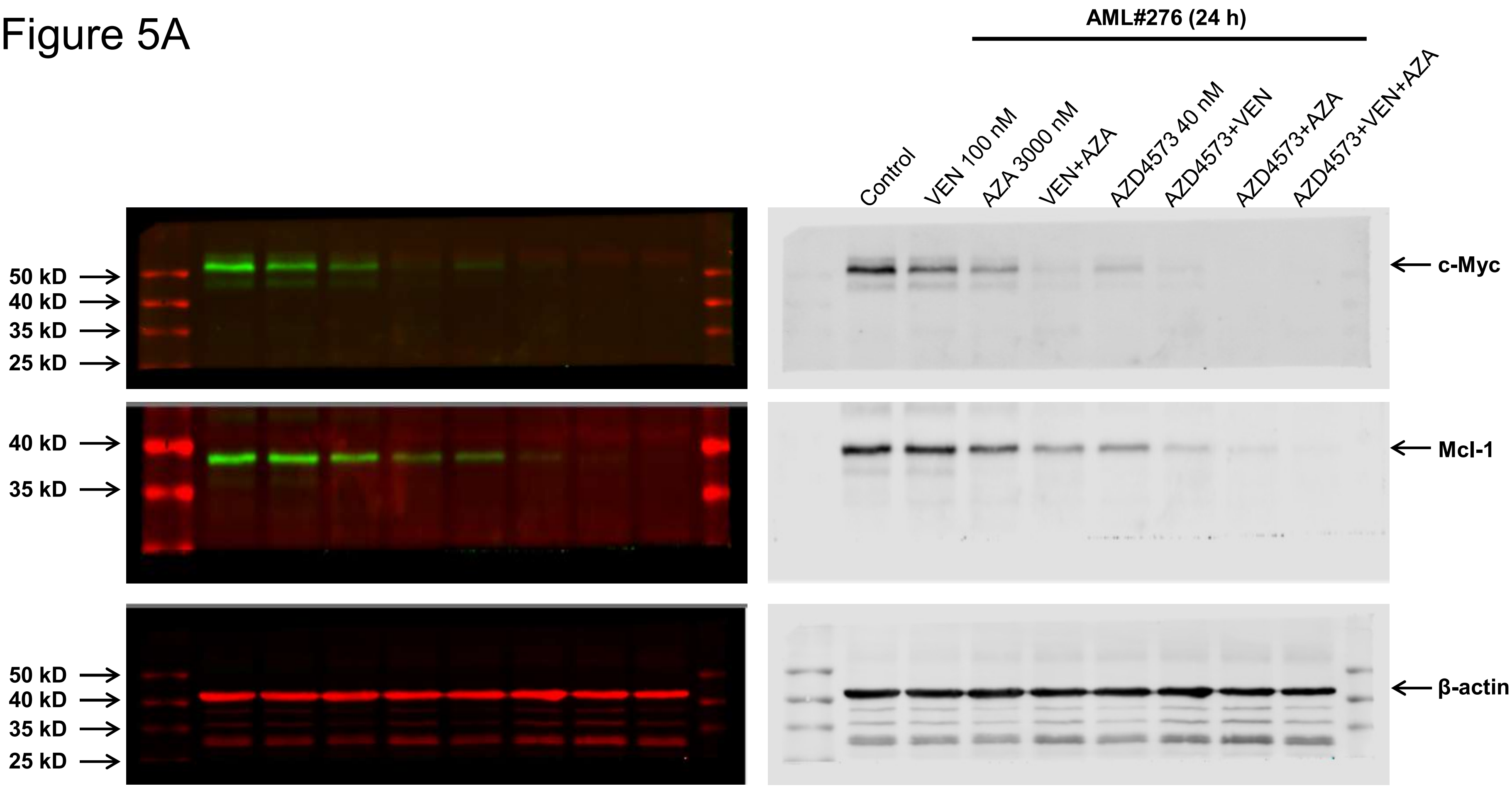
AML#276 (24 h)
Control
VEN 100 nM
AZA 3000 nM
VEN+AZA
AZD4573 40 nM
AZD4573+VEN
AZD4573+AZA
AZD4573+VEN+AZA
50 kD
40 kD
35 kD
25 kD
c-Myc
40 kD
35 kD
Mcl-1
50 kD
40 kD
35 kD
25 kD
β-actin

Figure 5A

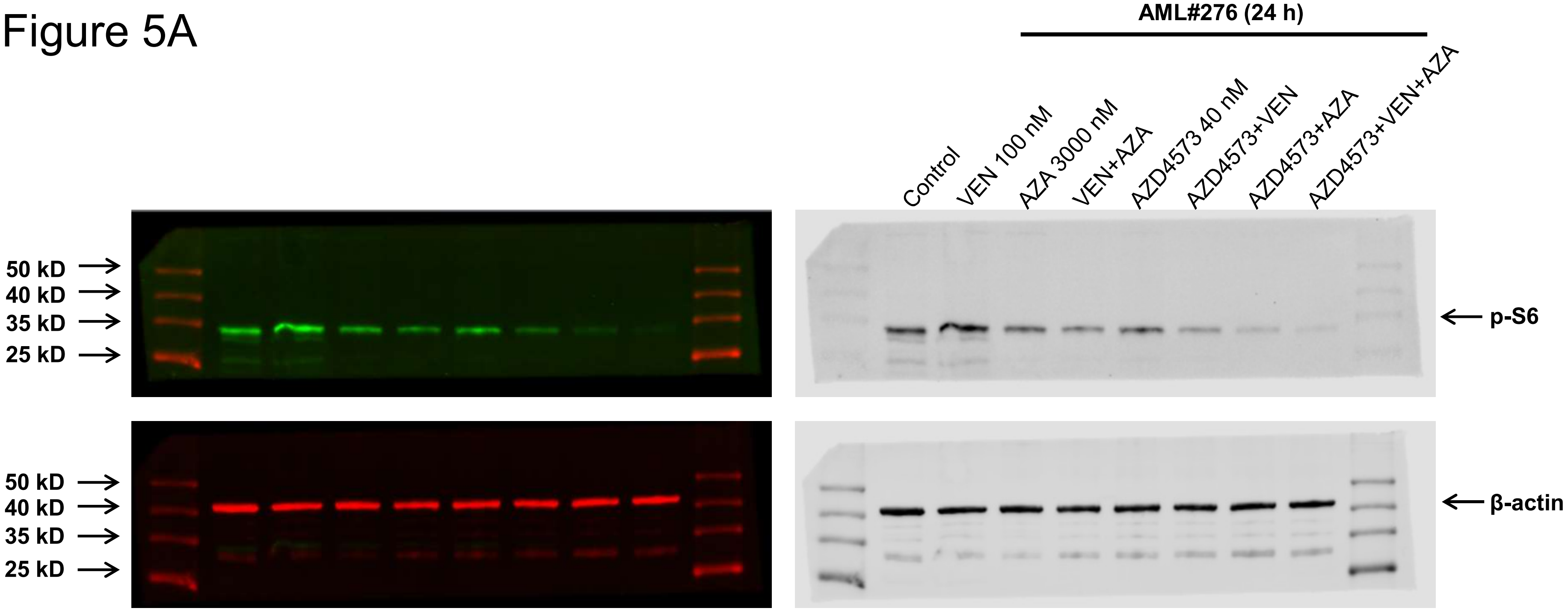
AML#276 (24 h)
Control
VEN 100 nM
AZA 3000 nM
VEN+AZA
AZD4573 40 nM
AZD4573+VEN
AZD4573+AZA
AZD4573+VEN+AZA
50 kD
40 kD
35 kD
25 kD
p-S6
50 kD
40 kD
35 kD
25 kD
β-actin

Figure 5B

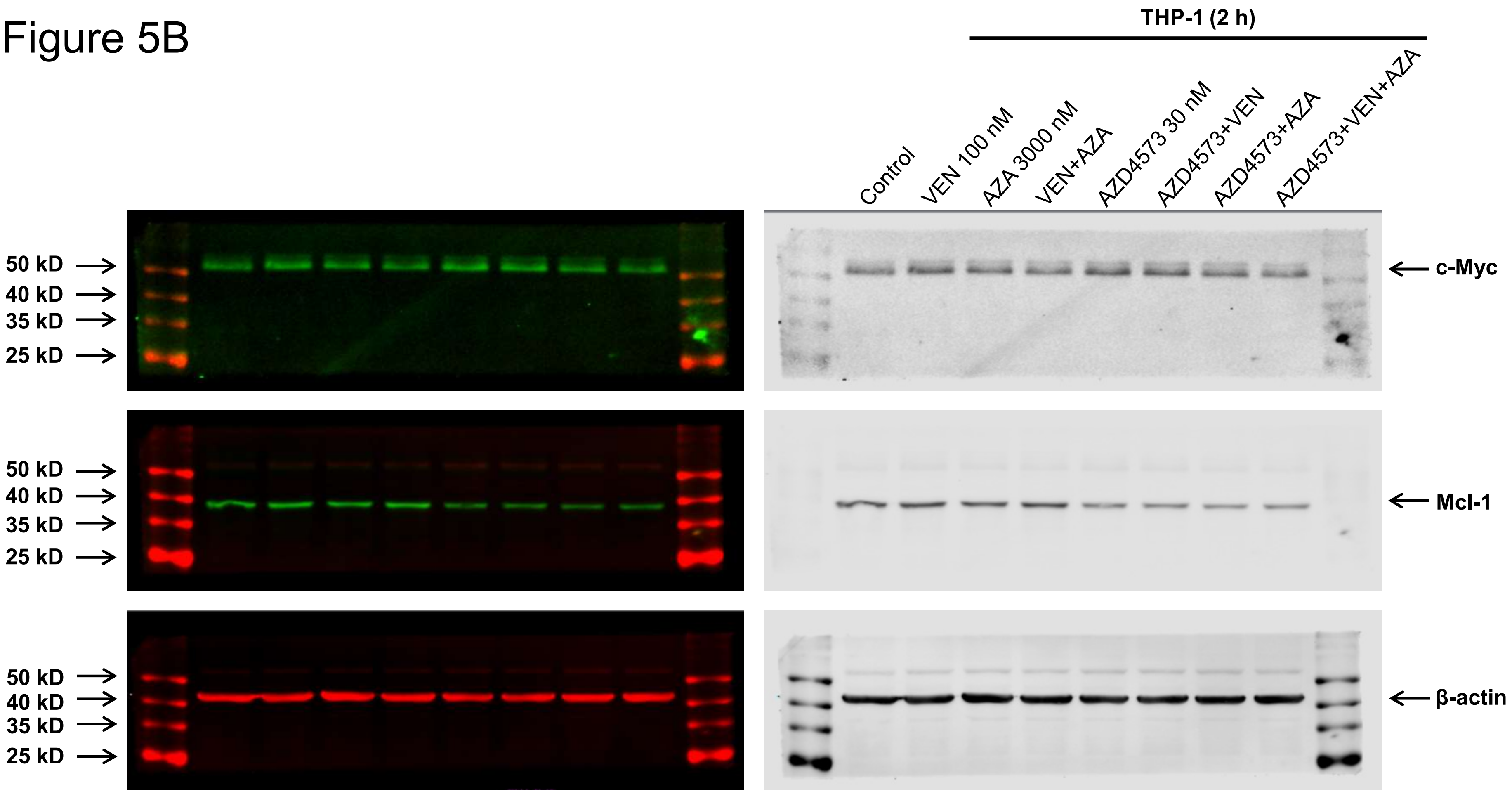
THP-1 (2 h)
Control
VEN 100 nM
AZA 3000 nM
VEN+AZA
AZD4573 30 nM
AZD4573+VEN
AZD4573+AZA
AZD4573+VEN+AZA
50 kD
40 kD
35 kD
25 kD
c-Myc
Mcl-1
β-actin

Figure 5B

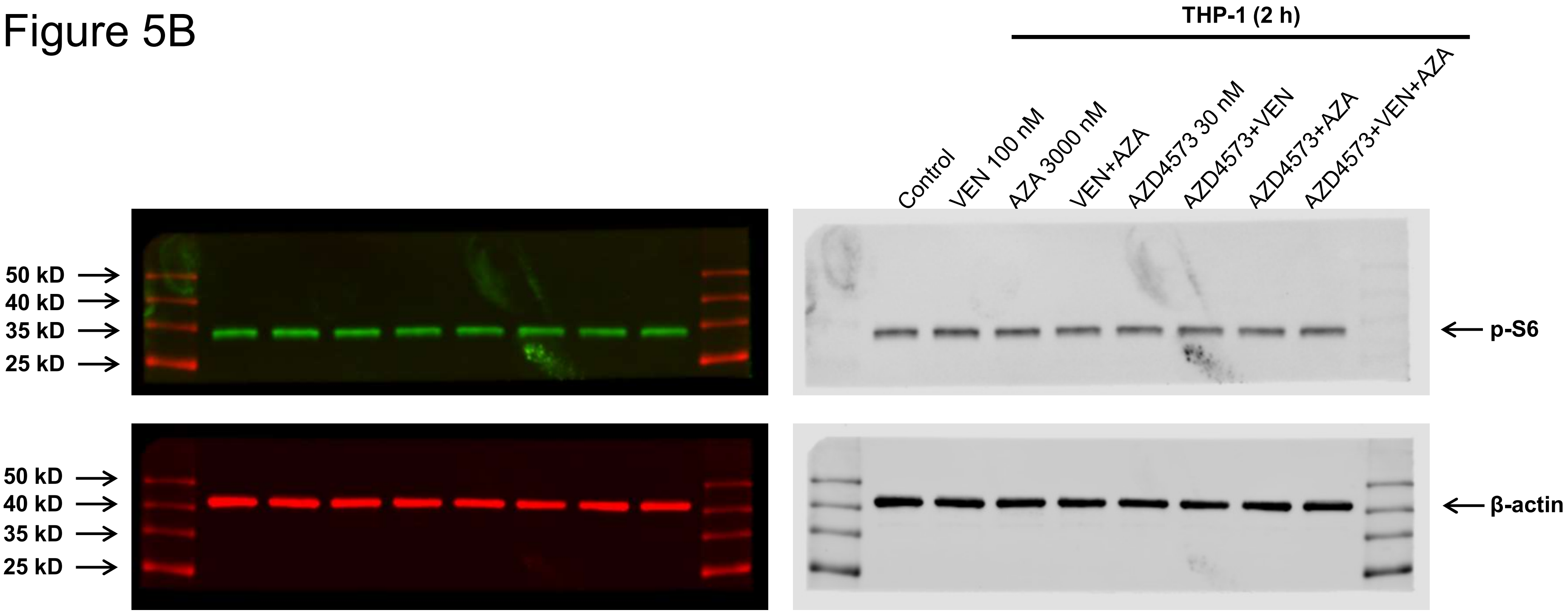
THP-1 (2 h)
Control
VEN 100 nM
AZA 3000 nM
VEN+AZA
AZD4573 30 nM
AZD4573+VEN
AZD4573+AZA
AZD4573+VEN+AZA
50 kD
40 kD
35 kD
25 kD
p-S6
50 kD
40 kD
35 kD
25 kD
β-actin

Figure 5B

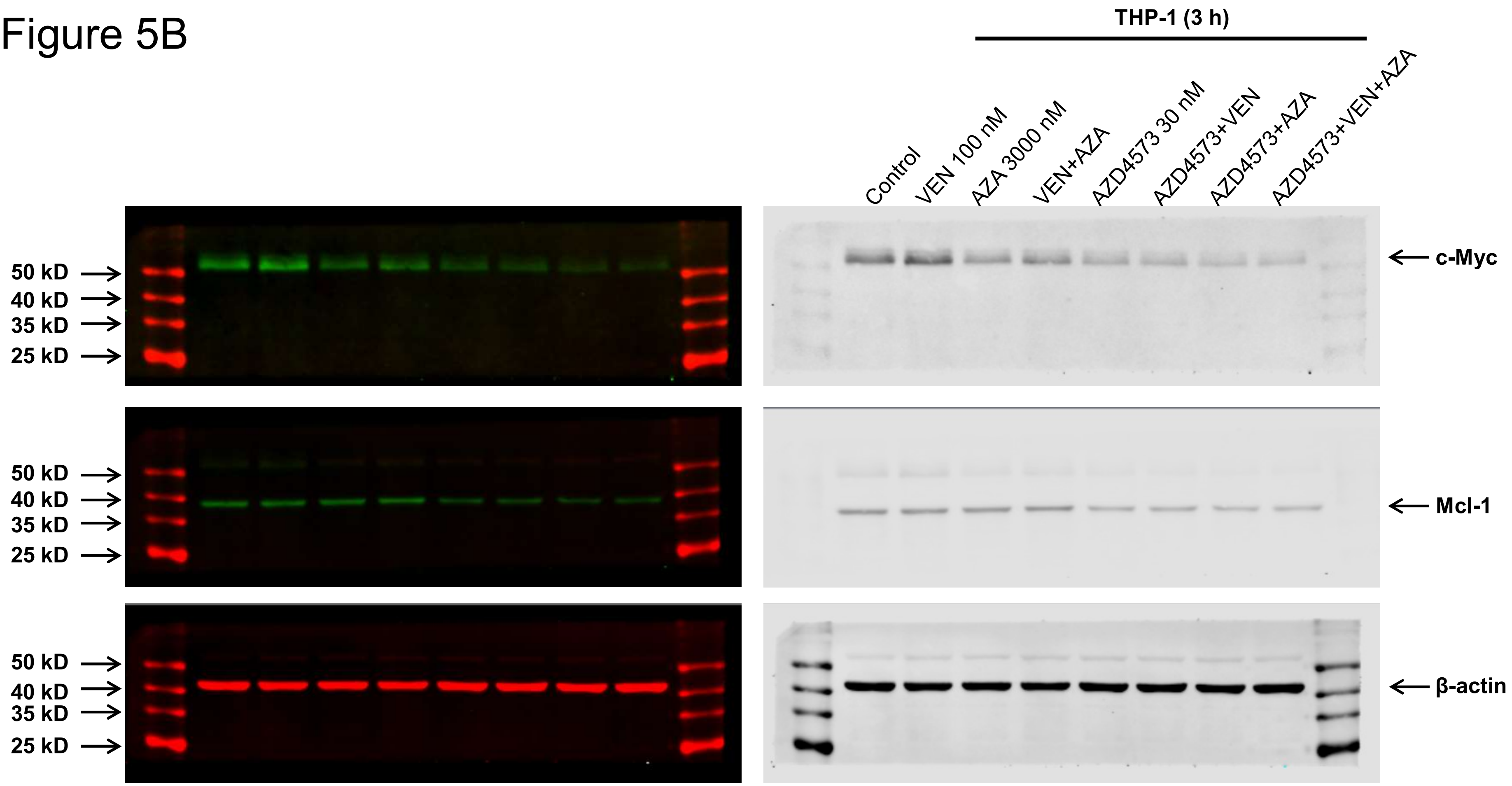

THP-1 (3 h)
Control
VEN 100 nM
AZA 3000 nM
VEN+AZA
AZD4573 30 nM
AZD4573+VEN
AZD4573+AZA
AZD4573+VEN+AZA
50 kD
40 kD
35 kD
25 kD
c-Myc
50 kD
40 kD
35 kD
25 kD
Mcl-1
50 kD
40 kD
35 kD
25 kD
β-actin

Figure 5B

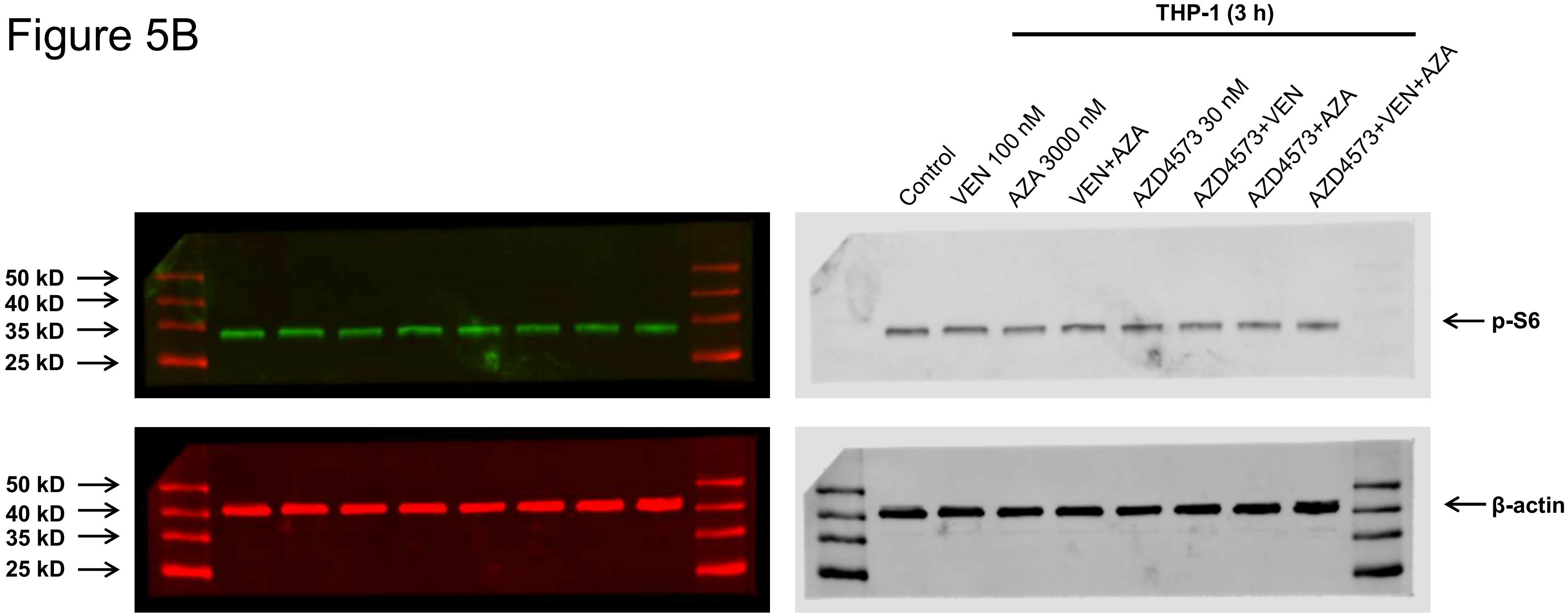

THP-1 (3 h)
Control
VEN 100 nM
AZA 3000 nM
VEN+AZA
AZD4573 30 nM
AZD4573+VEN
AZD4573+AZA
AZD4573+VEN+AZA
50 kD
40 kD
35 kD
25 kD
p-S6
β-actin

Figure 5B

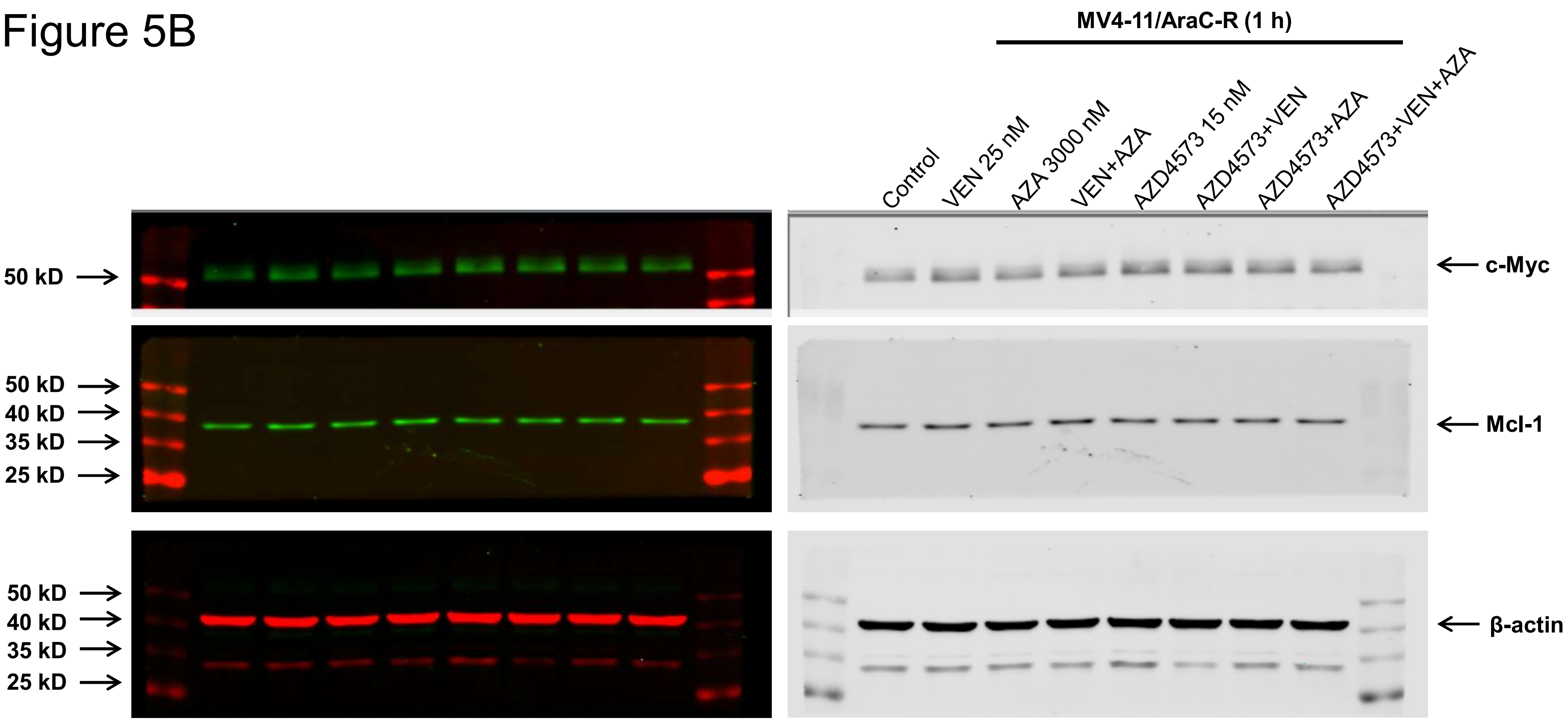
MV4-11/AraC-R (1 h)
Control
VEN 25 nM
AZA 3000 nM
VEN+AZA
AZD4573 15 nM
AZD4573+VEN
AZD4573+AZA
AZD4573+VEN+AZA
50 kD
c-Myc
50 kD
40 kD
35 kD
25 kD
Mcl-1
50 kD
40 kD
35 kD
25 kD
β-actin

Figure 5D

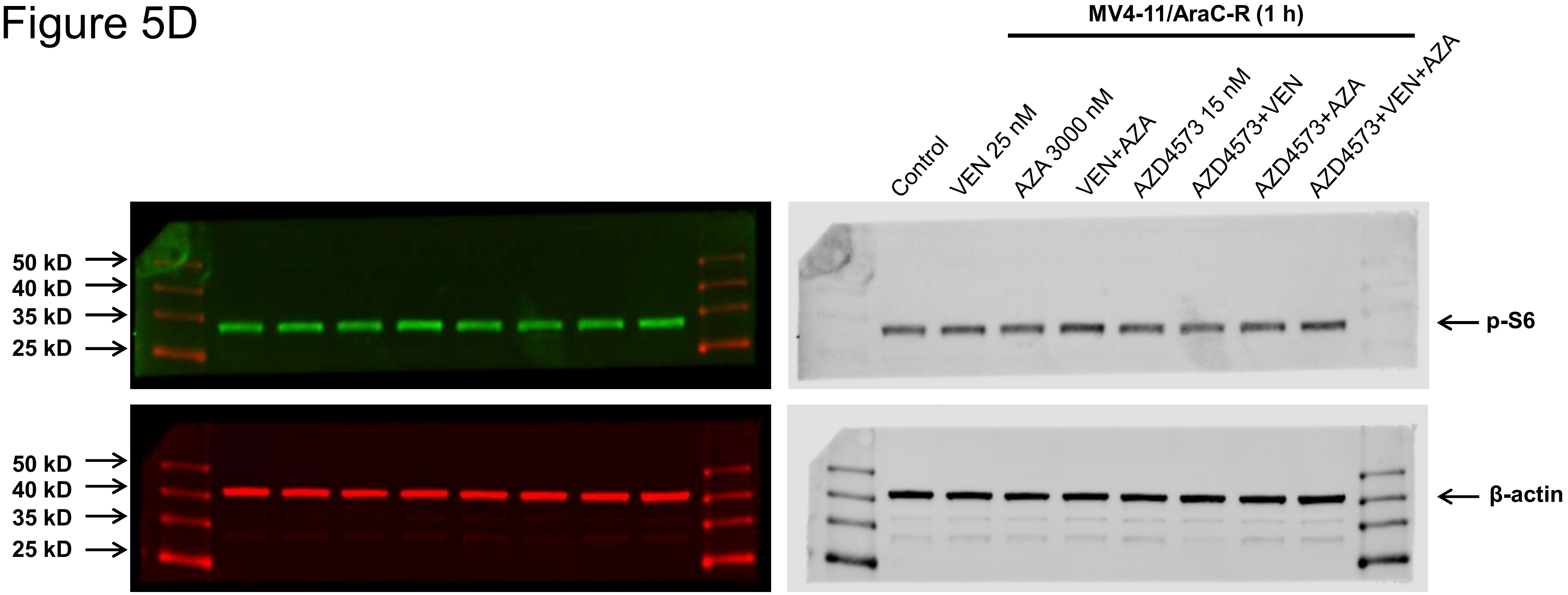
MV4-11/AraC-R (1 h)
Control
VEN 25 nM
AZA 3000 nM
VEN+AZA
AZD4573 15 nM
AZD4573+VEN
AZD4573+AZA
AZD4573+VEN+AZA
50 kD
40 kD
35 kD
25 kD
p-S6
50 kD
40 kD
35 kD
25 kD
β-actin

Figure 5D

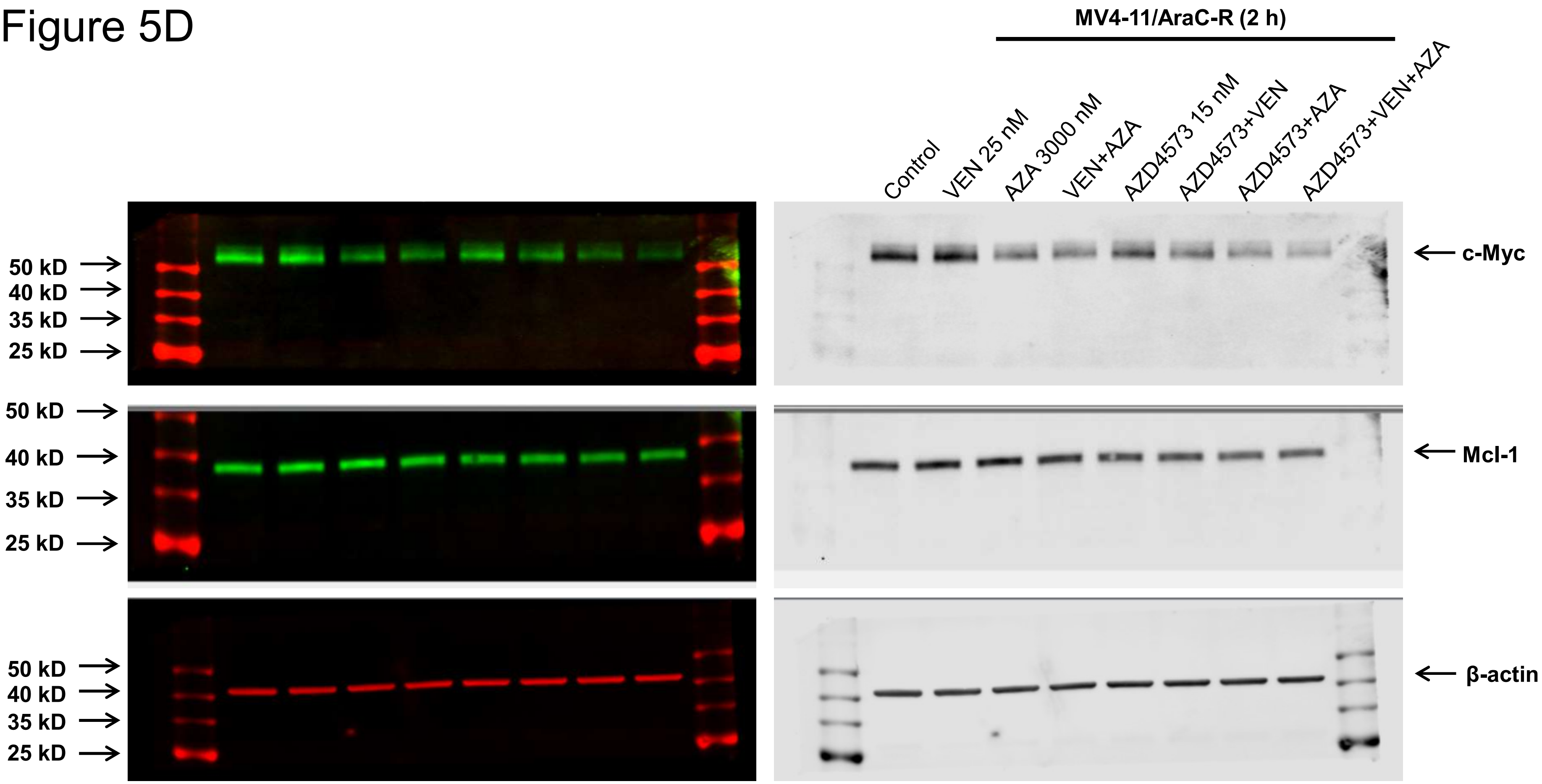

MV4-11/AraC-R (2 h)
Control
VEN 25 nM
AZA 3000 nM
VEN+AZA
AZD4573 15 nM
AZD4573+VEN
AZD4573+AZA
AZD4573+VEN+AZA
50 kD
40 kD
35 kD
25 kD
c-Myc
50 kD
40 kD
35 kD
25 kD
Mcl-1
50 kD
40 kD
35 kD
25 kD
β-actin

Figure 5D

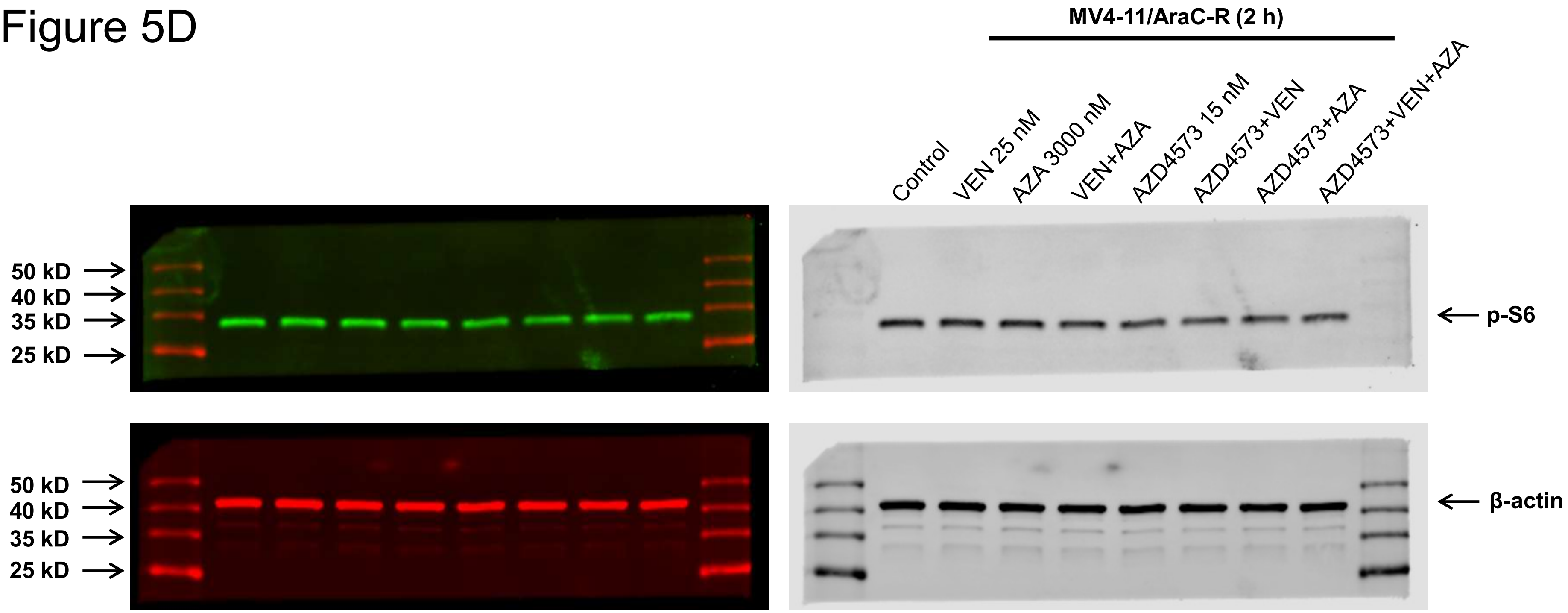
MV4-11/AraC-R (2 h)
Control
VEN 25 nM
AZA 3000 nM
VEN+AZA
AZD4573 15 nM
AZD4573+VEN
AZD4573+AZA
AZD4573+VEN+AZA
50 kD
40 kD
35 kD
25 kD
p-S6
50 kD
40 kD
35 kD
25 kD
β-actin

Figure 5D

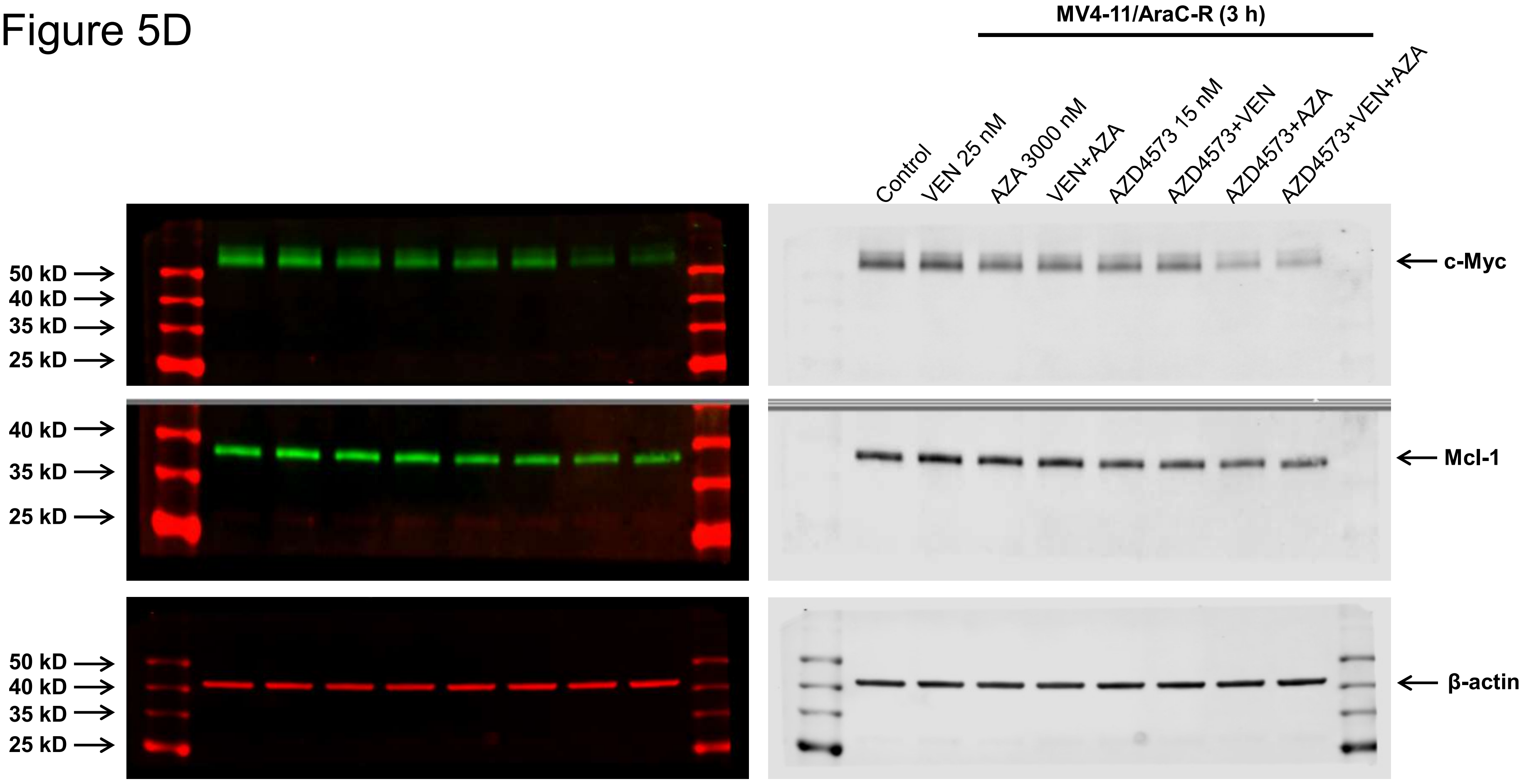
MV4-11/AraC-R (3 h)
Control
VEN 25 nM
AZA 3000 nM
VEN+AZA
AZD4573 15 nM
AZD4573+VEN
AZD4573+AZA
AZD4573+VEN+AZA
50 kD
40 kD
35 kD
25 kD
c-Myc
40 kD
35 kD
25 kD
Mcl-1
50 kD
40 kD
35 kD
25 kD
β-actin

Figure 5D

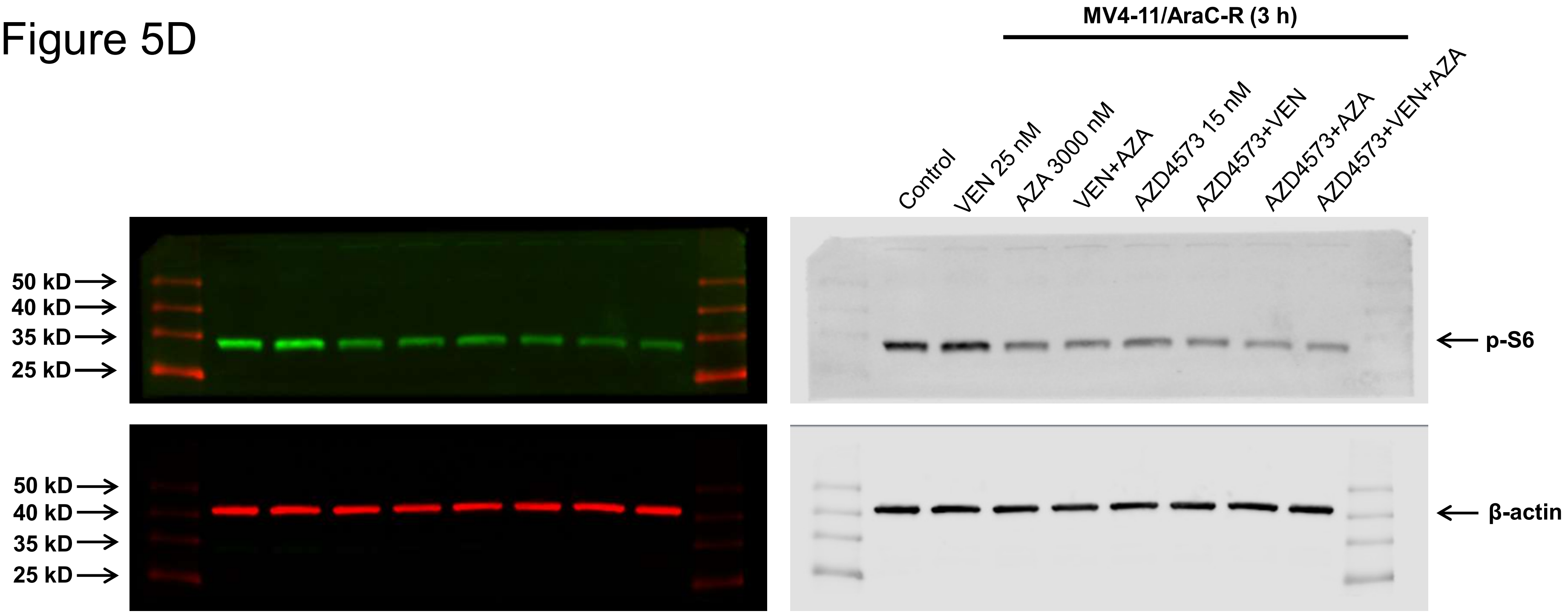

MV4-11/AraC-R (3 h)
Control
VEN 25 nM
AZA 3000 nM
VEN+AZA
AZD4573 15 nM
AZD4573+VEN
AZD4573+AZA
AZD4573+VEN+AZA
50 kD
40 kD
35 kD
25 kD
p-S6
50 kD
40 kD
35 kD
25 kD
β-actin

Figure 5F

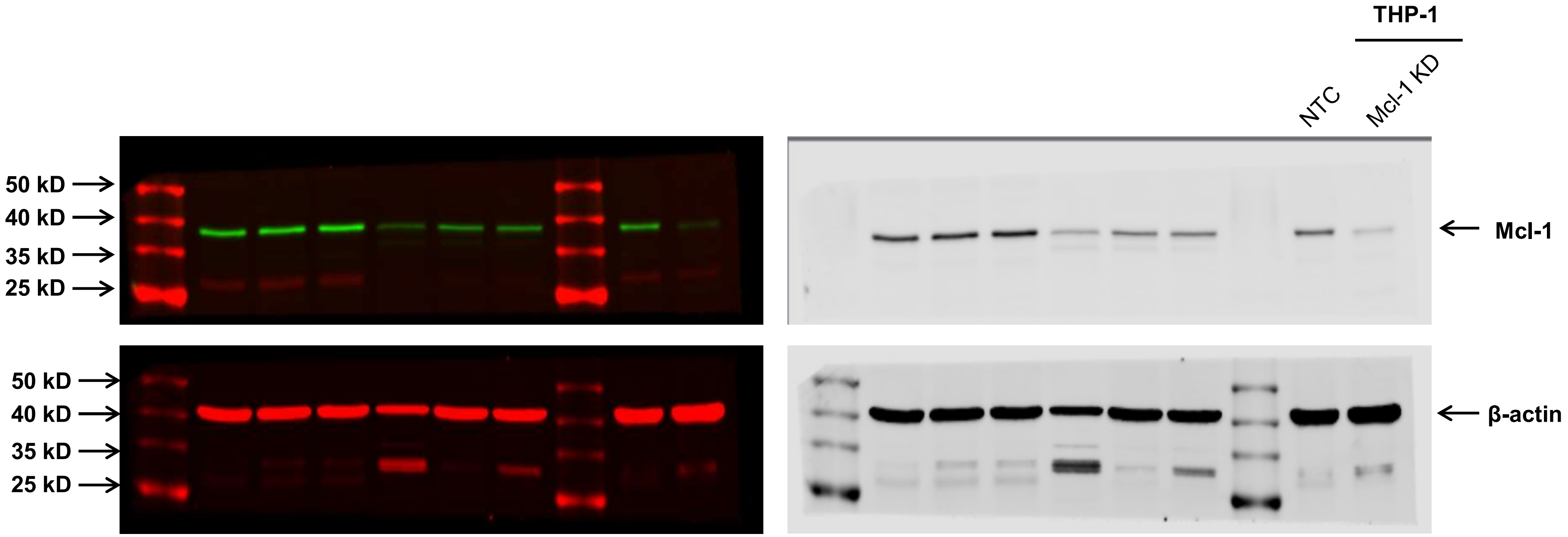

THP-1
NTC
Mcl-1 KD
50 kD
40 kD
35 kD
25 kD
Mcl-1
50 kD
40 kD
35 kD
25 kD
β-actin

Figure 5G

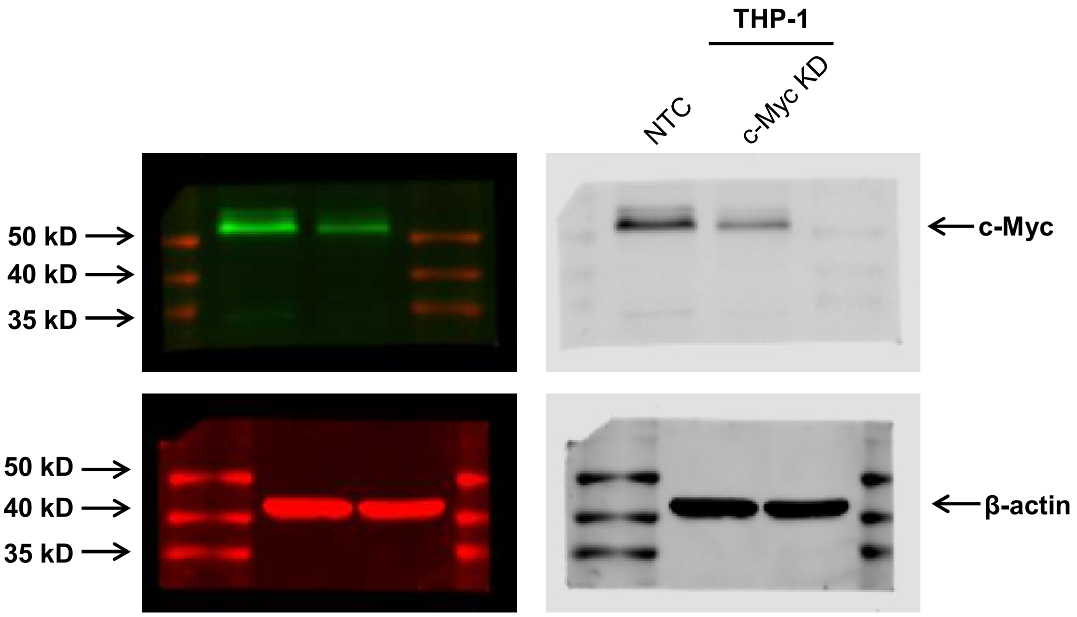

Figure 5H

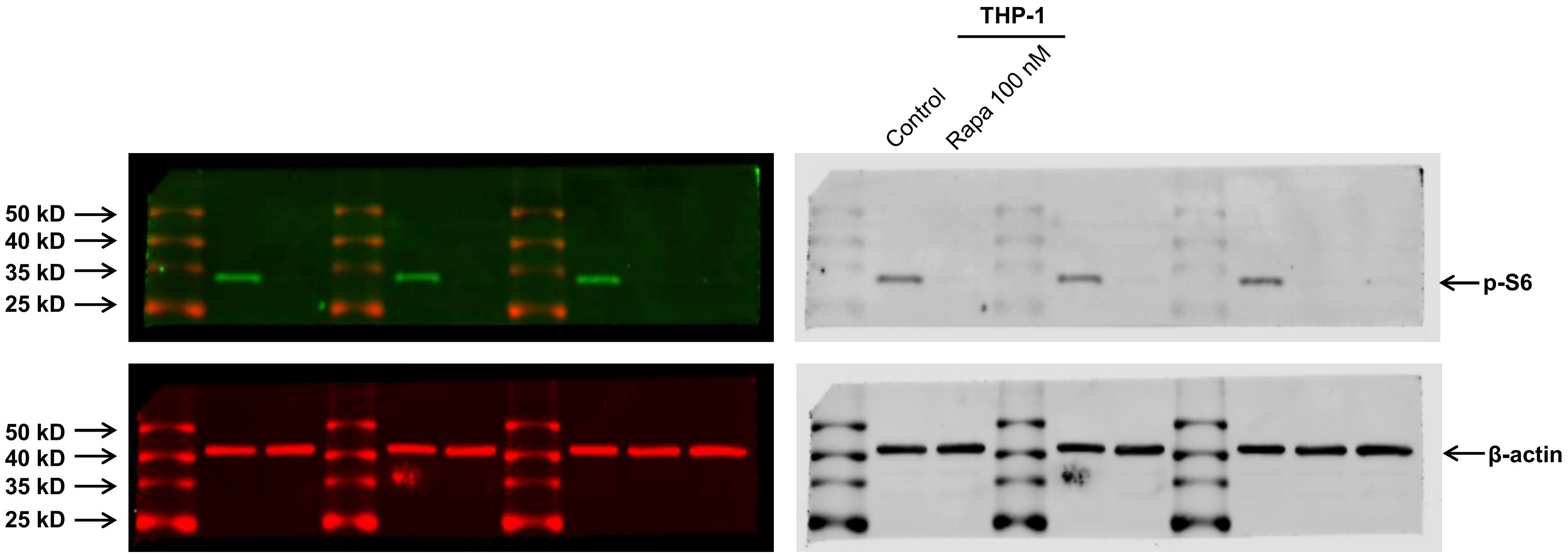
THP-1
Control
Rapa 100 nM
50 kD
40 kD
35 kD
25 kD
p-S6
50 kD
40 kD
35 kD
25 kD
β-actin

Figure 5I

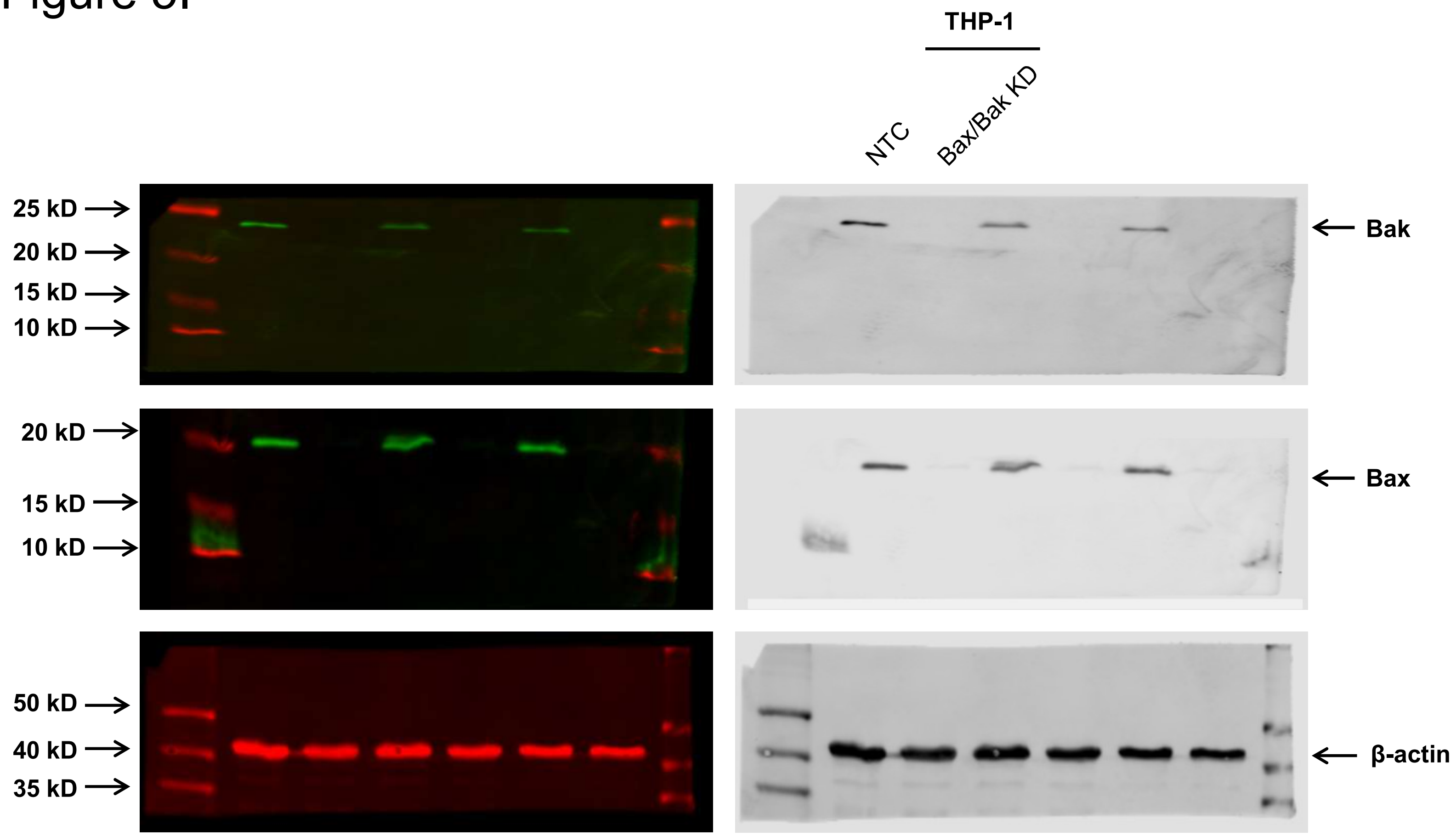

THP-1
NTC
Bax/Bak KD
25 kD
20 kD
15 kD
10 kD
Bak
20 kD
15 kD
10 kD
Bax
50 kD
40 kD
35 kD
β-actin

Figure S2A

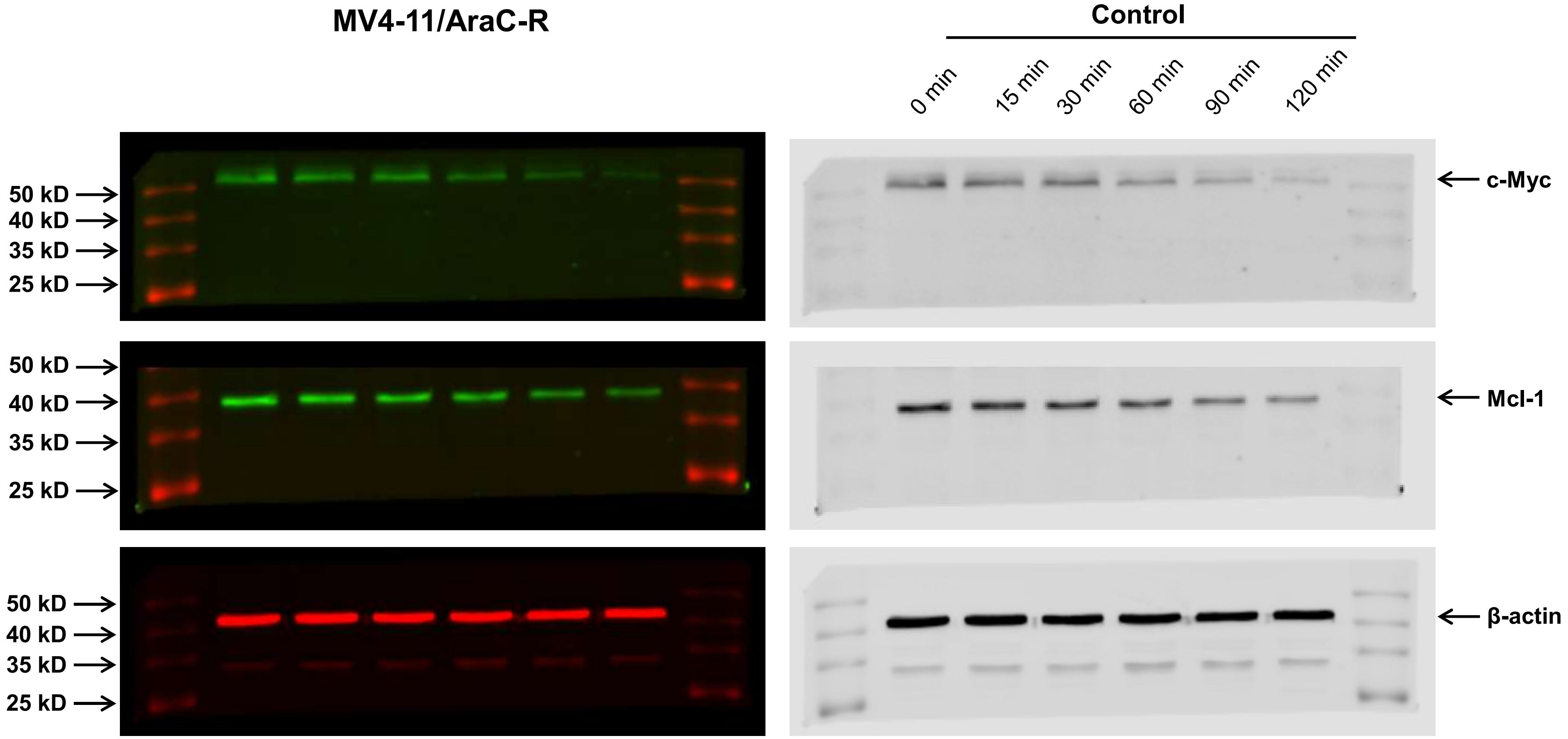
MV4-11/AraC-R
Control
0 min
15 min
30 min
60 min
90 min
120 min
50 kD
40 kD
35 kD
25 kD
c-Myc
Mcl-1
β-actin

Figure S2A

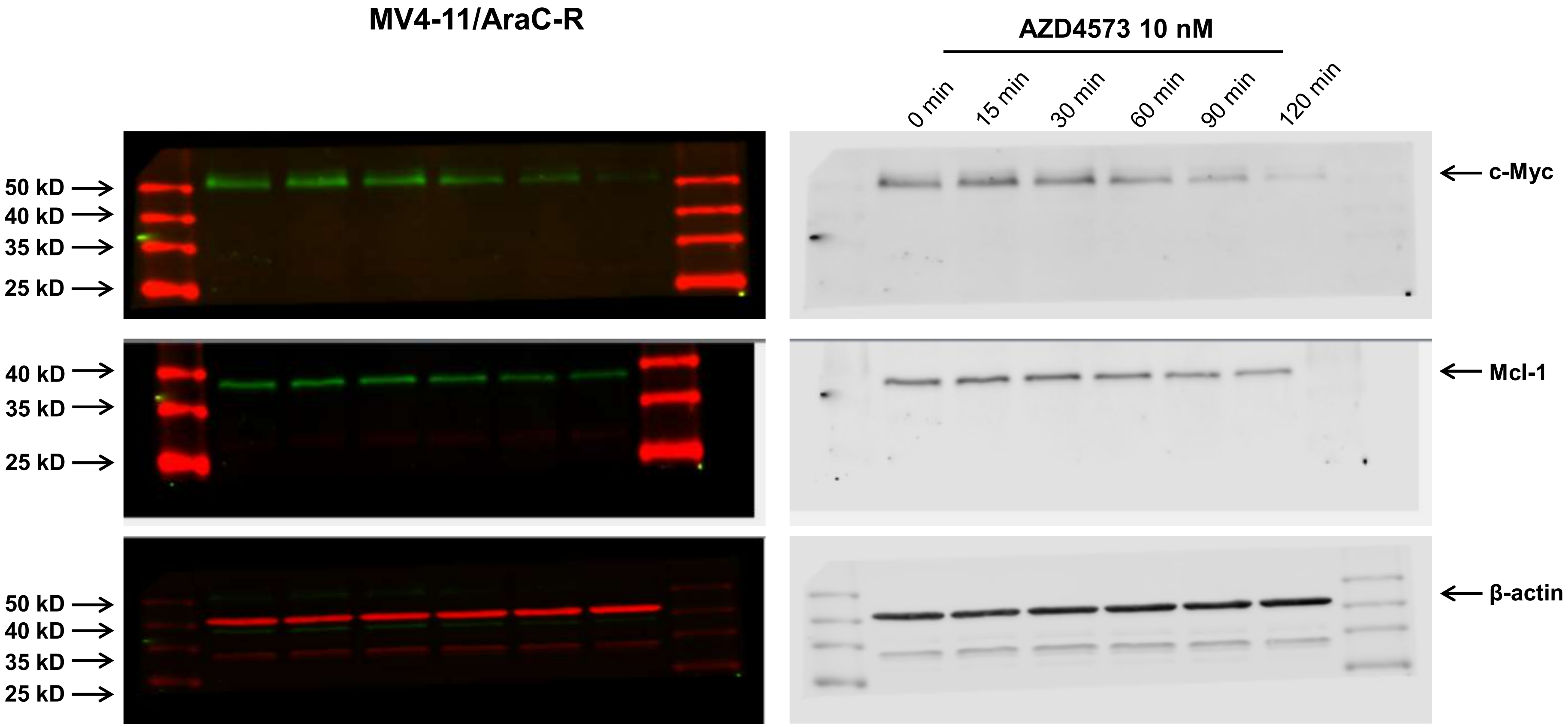

MV4-11/AraC-R
AZD4573 10 nM
0 min
15 min
30 min
60 min
90 min
120 min
50 kD
40 kD
35 kD
25 kD
c-Myc
40 kD
35 kD
25 kD
Mcl-1
50 kD
40 kD
35 kD
25 kD
β-actin

Figure S2B

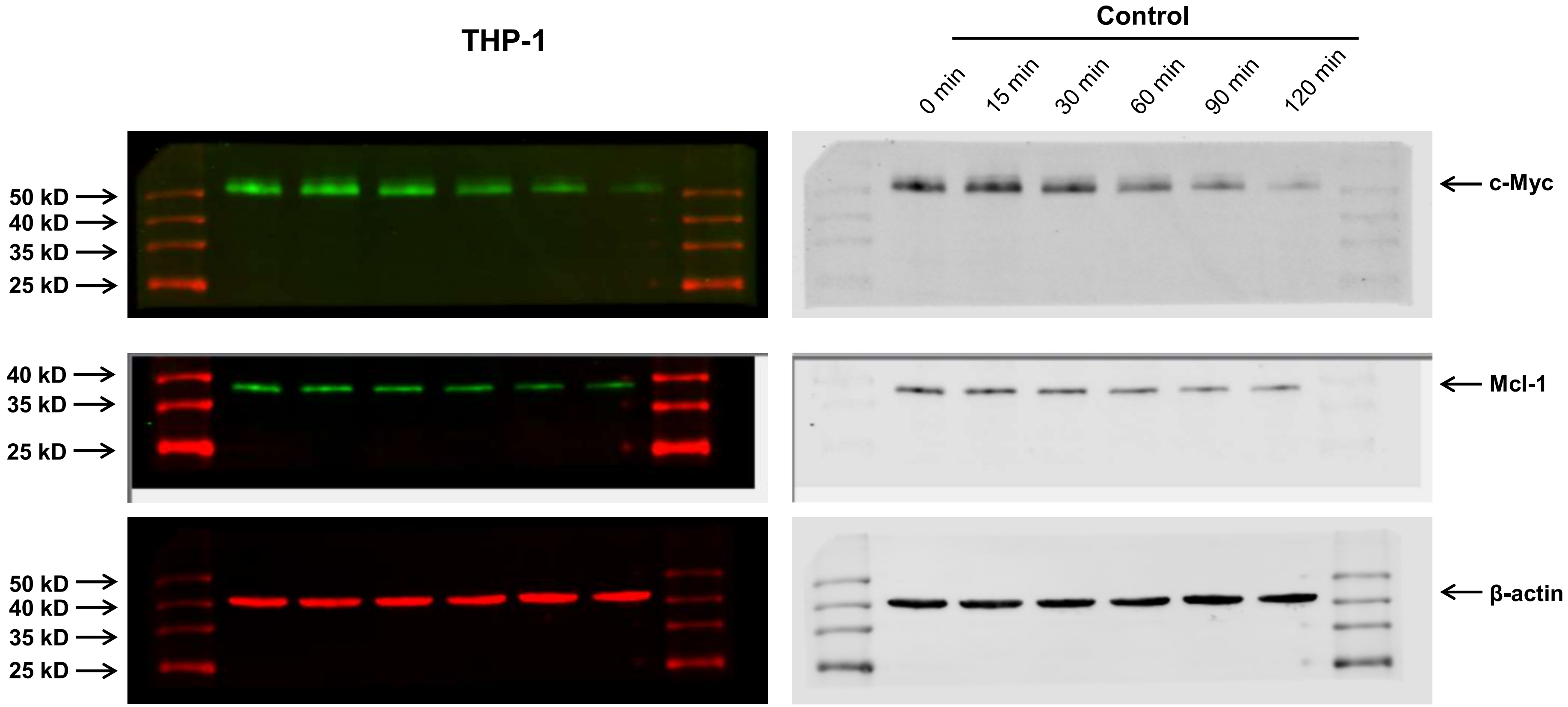
THP-1
Control
0 min
15 min
30 min
60 min
90 min
120 min
50 kD
40 kD
35 kD
25 kD
40 kD
35 kD
25 kD
50 kD
40 kD
35 kD
25 kD
c-Myc
Mcl-1
β-actin

Figure S2B

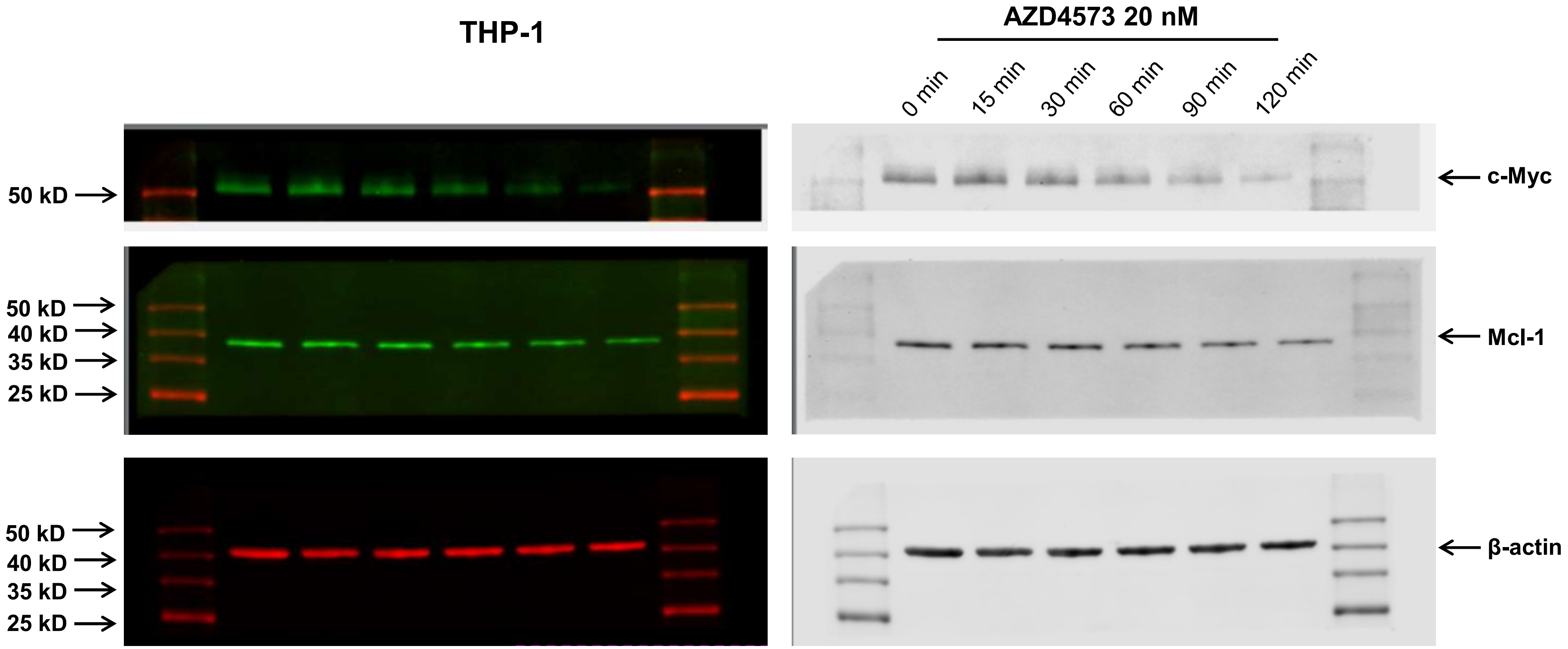
THP-1
AZD4573 20 nM
0 min
15 min
30 min
60 min
90 min
120 min
50 kD
50 kD
40 kD
35 kD
25 kD
50 kD
40 kD
35 kD
25 kD
c-Myc
Mcl-1
β-actin
